# Myonuclear permanence in skeletal muscle memory: a systematic review and meta‐analysis of human and animal studies

**DOI:** 10.1002/jcsm.13043

**Published:** 2022-08-12

**Authors:** Masoud Rahmati, John J. McCarthy, Fatemeh Malakoutinia

**Affiliations:** ^1^ Department of Physical Education and Sport Sciences, Faculty of Literature and Human Sciences Lorestan University Khorramabad Iran; ^2^ Department of Physiology University of Kentucky Lexington KY USA; ^3^ Center for Muscle Biology University of Kentucky Lexington KY USA

**Keywords:** Muscle memory, Myonuclei, Satellite cell, Hypertrophy, Ageing, Meta‐analysis

## Abstract

One aspect of skeletal muscle memory is the ability of a previously trained muscle to hypertrophy more rapidly following a period of detraining. Although the molecular basis of muscle memory remains to be fully elucidated, one potential mechanism thought to mediate muscle memory is the permanent retention of myonuclei acquired during the initial phase of hypertrophic growth. However, myonuclear permanence is debated and would benefit from a meta‐analysis to clarify the current state of the field for this important aspect of skeletal muscle plasticity. The objective of this study was to perform a meta‐analysis to assess the permanence of myonuclei associated with changes in physical activity and ageing. When available, the abundance of satellite cells (SCs) was also considered given their potential influence on changes in myonuclear abundance. One hundred forty‐seven peer‐reviewed articles were identified for inclusion across five separate meta‐analyses; (1–2) human and rodent studies assessed muscle response to hypertrophy; (3–4) human and rodent studies assessed muscle response to atrophy; and (5) human studies assessed muscle response with ageing. Skeletal muscle hypertrophy was associated with higher myonuclear content that was retained in rodents, but not humans, with atrophy (SMD = −0.60, 95% CI −1.71 to 0.51, *P* = 0.29, and MD = 83.46, 95% CI −649.41 to 816.32, *P* = 0.82; respectively). Myonuclear and SC content were both lower following atrophy in humans (MD = −11, 95% CI −0.19 to −0.03, *P* = 0.005, and SMD = −0.49, 95% CI −0.77 to −0.22, *P* = 0.0005; respectively), although the response in rodents was affected by the type of muscle under consideration and the mode of atrophy. Whereas rodent myonuclei were found to be more permanent regardless of the mode of atrophy, atrophy of ≥30% was associated with a reduction in myonuclear content (SMD = −1.02, 95% CI −1.53 to −0.51, *P* = 0.0001). In humans, sarcopenia was accompanied by a lower myonuclear and SC content (MD = 0.47, 95% CI 0.09 to 0.85, *P* = 0.02, and SMD = 0.78, 95% CI 0.37–1.19, *P* = 0.0002; respectively). The major finding from the present meta‐analysis is that myonuclei are not permanent but are lost during periods of atrophy and with ageing. These findings do not support the concept of skeletal muscle memory based on the permanence of myonuclei and suggest other mechanisms, such as epigenetics, may have a more important role in mediating this aspect of skeletal muscle plasticity.

## Introduction

Skeletal muscle fibres are some of the largest cells in the body and uniquely multinucleated with more than one hundred myonuclei per mm length of fibre.^1^ In order to maximize the distance between neighbouring nuclei, all nuclei within the syncytium are evenly positioned, adjacent to the plasma membrane.^2^ More interestingly, skeletal muscle is an extraordinary tissue with the ability to respond to intrinsic and extrinsic stimuli by changing its size.^3^ Myonuclei have an important role in skeletal muscle size adaptation through the production of transcripts that support the synthesis of proteins for use in the immediate vicinity surrounding each nucleus.^4^

In response to exercise, new myonuclei can be acquired by myofibres as the result of fusion by muscle stem cells (known as satellite cells), which are normally in a quiescent state and become activated upon exposure to external stimuli, such as exercise or injury. Once activated, satellite cells (SCs) proliferate, differentiate into myogenic progenitor cells, and subsequently fuse to existing myofibres, providing additional nuclei to the growing myofibres.^5^, ^6^, ^7^ Studies have provided evidence showing that each nucleus within a myofibre oversees a given amount of cytoplasm, which is referred to as the myonuclear domain.^3^, ^4^ The notion of a myonuclear domain is based on the concept that each nucleus has a limited capacity to control transcriptional characteristics over a finite volume of cytoplasm.^4^, ^8^ Further, other studies have suggested the size of the myonuclear domain may not be as fixed as is often indicated.^9^, ^10^

Skeletal muscle possesses the remarkable ability to ‘recall’ a previous hypertrophic state upon resumption of training following a period of detraining, a phenomenon that has been called ‘muscle memory’.^11^, ^12^, ^13^ Scientists first attributed the phenomenon of muscle memory to motor learning via the central nervous system.^14^ The findings from more recent studies have proposed that muscle memory is related to the abundance of myonuclei, with the new myonuclei added during the initial hypertrophy being permanent, thereby providing enhanced transcriptional output in response to training following a bout of detraining.^15^, ^16^ It has been hypothesized that the retention of the hyper‐nucleated condition might be responsible for the accelerated regeneration and return of myofibre size and function even after a prolonged period of inactivity in previously trained skeletal muscle.^16^ Current available evidence regarding muscle memory is quite conflicting with some reports confirming myonuclear permanence,^15^, ^16^, ^17^, ^18^ although other studies showing myonuclei could be lost during detraining.^19^, ^20^, ^21^, ^22^ Some studies have reported that myonuclear content in skeletal muscle is not permanent and undergoes apoptosis with atrophy in response to hindlimb suspension,^23^, ^24^ denervation,^25^, ^26^ exposure to microgravity,^27^ and immobilization.^28^ Moreover, recent studies in both rodents^21^ and humans^22^, ^29^, ^30^ have shown that myonuclei acquired during hypertrophy are not permanent following long‐term inactivity with myonuclear abundance returning to previously untrained state.

To the best of our knowledge, no systematic review and meta‐analysis has yet assessed whether hypertrophy‐induced myonuclear accretion is maintained after exercise cessation or inactivity in both humans and rodents. The aim of this systematic review and meta‐analysis was to assess myonuclear and SC content in skeletal muscle that underwent hypertrophy or atrophy in both humans and rodents. Finally, the long‐term myonuclear permanence in human was assessed by the inclusion of ageing studies in the meta‐analyses.

## Methods

The present preclinical and clinical review was registered in the International Prospective Register of Systematic Reviews (PROSPERO) with the registration number: CRD42020152068 and was performed in accordance with PRISMA guidelines.^31^

### Research question

In the present systematic review and meta‐analysis, we sought to answer the following questions: (i) Is hypertrophy‐induced myonuclear accretion maintained after exercise cessation in either humans and/or rodents? (ii) Does myonuclear content and/or SC abundance change during atrophy in either humans or rodents? (iii) Is there any difference in myonuclear content and/or SC abundance between elderly and young adults?

### Data sources and searches

A systematic literature search for relevant studies was carried out using the following databases: CINAHL, MEDLINE, CENTRAL, PEDro, ProQuest, and Scopus, from the earliest record of each database up to February 2022. Search terms included a combination of the following keywords related to muscle memory: ‘muscle memory’ and ‘memory’; related to muscle CSA: ‘muscle hypertrophy’, ‘muscle atrophy’, ‘myonuclei’, ‘myonuclear domain’, ‘satellite cell’, and ‘muscle stem cell’; related to training: ‘resistance exercise’, ‘resistance training’, ‘strength training’, ‘power training’, ‘endurance exercise’, and ‘endurance training’; related to atrophy stimuli: ‘loading’, ‘unloading’, ‘hindlimb suspension’, ‘suspension’, ‘leg immobilization’, ‘immobilization’, ‘step reduction’, ‘denervation’, ‘spinal cord injury’ and ‘spinal cord transaction’; and related to human ageing: ‘sarcopenia’, ‘human Aging’, ‘aging’, and ‘elderly’.

### Study selection

**Figure 1:**
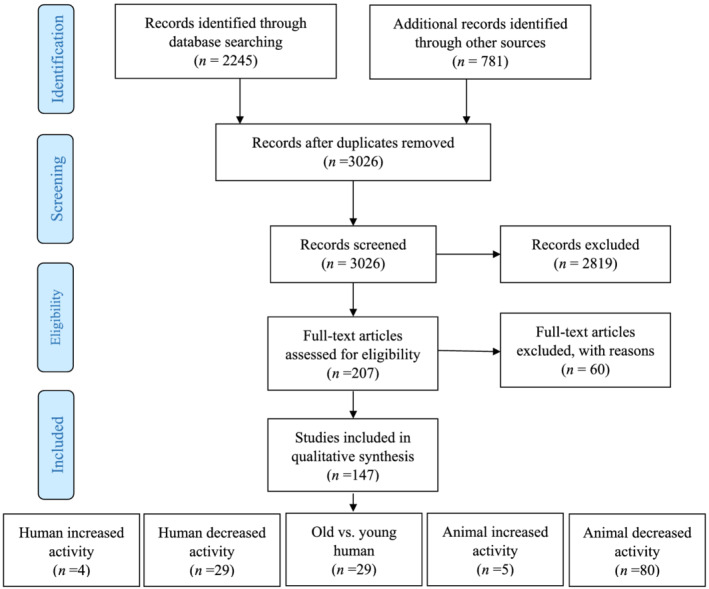
PRISMA flow diagram of study selection.

We included all studies involving human and animal models independent of sex, age, and intervention (except steroid administration) that evaluated satellite cell or myonuclear abundance. In terms of study design, both controlled and uncontrolled clinical trials were included in the systematic review and meta‐analysis (*Figure* *1*).

### Quality assessment

We assessed potential study bias using Physiotherapy Evidence Database (PEDro) scale for human studies by two independent researchers.^32^ All included human studies presented a score of ≤5.0. We also used the Systematic Review Centre for Laboratory Animal Experimentation (SYRCLE) tool for assessing the risk of bias in animal studies.^33^ The results of quality assessments in both human and animal studies are outlined in *Figure* *S1A* *–* *S1E*.

### Data extraction

Two reviewers independently (MR and FM) extracted all related information with disagreements between reviewers resolved by discussion. The included information was collected and organized into *Tables* *1*, *2*, *3*. Information was extracted on study design characteristics (rodent species, sex, age, hypertrophy or atrophy model, etc.), type of intervention (training or atrophy duration), and outcome data (myonuclear content and satellite cell abundance). Included studies were grouped according to the following experiments: human subjects experienced hypertrophy, human subjects experienced atrophy, comparison of old vs. young people, animal models experienced hypertrophy, and animal models experienced atrophy.

**Table 1:** The effect of hypertrophy or atrophy on myonuclear content and domain size and satellite cell content in humans

Author	Participants (number, sex)	Age	Muscle	Hypertrophy/Atrophy model	Training/Atrophy duration	Detraining duration	Muscle fibre size	Myonuclear content	Myonuclear domain	SC content
Kadi *et al*. (2004)^36^	Young (15, M)	24 ± 1	VL	Resistance training	12 wk	12 wk	**Training:** Mixed: ↑ **Detraining:** Mixed: ↓	**Training:** Mixed: ↔ **Detraining:** Mixed: ↔	NM	**Training:** Mixed: ↑ **Detraining:** Mixed: ↓
Psilander *et al*. (2019)^a^, ^29^	Young (10, W & 9, M)	25 ± 1	VL	Resistance training	10 wk	20 wk	**Training:** Mixed, I, *II*: ↔ **Detraining:** Mixed, I, *II*: ↔	**Training:** Mixed, I, *II*: ↔ **Detraining:** Mixed, I, *II*: ↔	**Training:** Mixed, I: ↔; *II*: ↔ **Detraining:** Mixed, I, *II*: ↔	NM
Snijders *et al*. (2019)^30^	Old (53, M/W)	70 ± 6	VL	Resistance training	24 wk	48 wk	**Training:** Mixed, *II*: ↑; I: ↔ **Detraining:** Mixed, *II*: ↓; I: ↔	**Training:** Mixed, I: ↔; *II*: ↑ **Detraining:** Mixed, I: ↔; *II*: ↓	**Training:** Mixed, I, II: ↔ **Detraining:** Mixed, I, II: ↔	**Training:** Mixed, I: ↔; *II*: ↑ **Detraining:** Mixed, I: ↔; *II*: ↓
Blocquiaux *et al*. (2020)^35^	Old (30, M)	66 ± 5	VL	Resistance training	12 wk	12 wk	**Training:** I, II: ↔ **Detraining:** I, II: ↔	**Training:** I, II: ↔ **Detraining:** I: ↓; *II*: ↔	**Training:** I, II: ↔ **Detraining:** I, II: ↔	**Training:** I, II: ↔ **Detraining:** I, II: ↔
Carlson *et al*. (2009)^38^	Young (11, M); Old (9,M)	22 ± 2; 71 ± 3	VL	Leg immobilization	2 wk	NA	***Young and Old***: Mixed: ↓	NM	NM	***Young and Old***: Mixed: ↔
Dirks *et al*. (2014a)^39^	Old (12, M)	69 ± 1	VL	Leg immobilization	5 d	NA	Mixed: ↓; *I, II*: ↔	*I, II*: ↔	*I, II*: ↓	*I, II*: ↔
Snijders *et al*. (2014)^40^	Young (12, M)	24 ± 1	VL	Leg immobilization	2 wk	NA	*I, II*: ↓	*I, II*: ↔	*I, II*: ↔	*I, II*: ↔
Dirks *et al*. (2014b)^41^	Young (12, M)	23 ± 1	VL	Leg immobilization	5 d	NA	Mixed: ↓; *I, II*: ↔	*I, II*: ↔	*I, II*: ↔	*I, II*: ↔
Suetta *et al*. (2013)^42^	Young (11, M); Old (9,M)	25 ± 4; 67 ± 6	VL	Leg immobilization	2 wk	NA	***Young:** I, II*: ↓; ***Old**: I*: ↔*, II*: ↓	NM	NM	***Young:** I, II*: ↑; ***Old**: I, II*: ↔
Ohira *et al*. (1999)^43^	Young (13, M)	33 ± 3	Soleus	Bed rest	2 and 4 mos	NA	Mixed: 2mon: ↔, 4mon: ↓	Mixed: 2 and 4 mos: ↔	Mixed: 2 mos: ↔, 4 mos: ↓	NM
Brooks *et al*. (2010)^44^	Young (7, M)	40 ± 15	VL	Bed rest	28 d	NA	Mixed: ↓	Mixed: ↔	NM	Mixed: ↔
Arentson‐Lantz *et al*. (2016)^19^	Young (7, M/W)	51 ± 1	VL	Bed rest	2 wk	NA	Mixed, I, II: ↓	Mixed, I, II: ↓	Mixed: ↓	Mixed, I, II: ↓
Reidy *et al*. (2017)^45^	Old (9, M/W)	69 ± 2	VL	Bed rest	5 d	NA	Mixed, I: ↓; *II*: ↔	Mixed, I, II: ↔	Mixed, I, II: ↔	Mixed, I: ↓; *II*: ↔
Reidy *et al*. (2018)^46^	Young (14, M/W); Old (9, M/W)	23 ± 1; 66 ± 1	VL	Bed rest	5 d	NA	***Young and Old*:** Mixed, *I, II*: ↔	NM	NM	***Young and Old*:** Mixed, *I, II*: ↔
Moore *et al*. (2018)^47^	Old (14, M)	71 ± 5	VL	Step reduction	14 d	NA	*I*: ↔, *II*: ↓	*I, II*: ↔	*I, II*: ↓	*I*: ↔, *II*: ↓
Reidy *et al*. (2019)^48^	Old (12, M)	70 ± 2	VL	Step reduction	7 and 14 d	NA	*7 and 14 d in* Mixed: ↔	*7 and 14 d in* Mixed, I, II: ↔	NM	*7 and 14 d in I*: ↑; *II*: ↔
Smith *et al*. (2013)^49^	Young (8, M/W); CP (8, M/W)	16 ± 2; 11 ± 4	VL	CP	NA	NA	NM	NM	NM	Mixed: ↓
Dayanidhi *et al*. (2015)^50^	Children (6, M)	13 ± 3	Gracilis	CP	NA	NA	Mixed: ↓	Mixed: ↔	Mixed: ↔	Mixed: ↓
Von Walden *et al*. (2018)^51^	Children and adolescents (22, M/W)	15 ± 7	VL	CP and brain injury	NR	NA	Mixed: ↑	NM	NM	Mixed: ↓
Eliason *et al*. (2009)^52^	Old (12, M/W); Moderate COPD (12, M/W); Severe COPD (11, M/W)	62 ± 6.6	Tibial anterior	COPD	NR	NA	Moderate COPD: *I, II*: ↔ Severe COPD: I: ↔; II: ↓	NM	NM	Moderate COPD: Mixed: ↔ Severe COPD: Mixed: ↔
Menon *et al*. (2012)^53^	Old (7, M/W); COPD (12, M/W)	67 ± 2	VL	COPD	NR	NA	*I, II*: ↔	NM	NM	Mixed: ↔
Thériault *et al*. (2012)^54^	Old (12, M/W); Moderate COPD (12, M/W); Severe COPD (11, M/W)	67 ± 3; 64 ± 2 70 ± 2	VL	COPD	NR	NA	Moderate COPD: *I*: ↔*, II*: ↓ Severe COPD: I, II: ↓	NM	NM	Moderate COPD: Mixed: ↔ Severe COPD: Mixed: ↔
Sancho‐Muñoz *et al*. (2021)^55^	Old (13, M/W); Non SAR (19, M/W); SAR (26, M/W)	66 ± 5; 65 ± 7 62 ± 8	VL	COPD	NR	NA	Non SAR: *I*: ↓*, II*: ↔ SAR: I, II: ↓	NM	NM	Non SAR *in* Mixed: ↔ SAR *in* Mixed: ↔
Noehren *et al*. (2016)^56^	Young (10, M/W)	23 ± 5	VL	ACL injury	12 wk	NA	I: ↔; II: ↓	NM	NM	Mixed: ↓
Fry *et al*. (2017)^57^	Young (10, M/W)	23 ± 5	VL	ACL injury	8 wk	NA	NM	NM	NM	Mixed, I, II: ↓
Parstorfer *et al*. (2021) ^58^	Young (1, W; 15, M)	26 ± 4	VL	ACL injury	12 wk	NA	*I, II*: ↔	NM	NM	Mixed, I, II: ↓
Day *et al*. (1995)^b^, ^20^	Young (5, M/W)	40 ± 7	VL	Space flight	11 d	NA	*I*: ↔; *II*: ↓	*I*: ↔; *II*: ↓	*I*: ↔; *II*: ↓	NM
Dirks *et al*. (2015)^59^	Old (6, M/W)	63 ± 6	VL	ICU patients	NA	NA	I, II: ↓	*I, II*: ↔	*I, II*: ↔	*I, II*: ↔
Kramer *et al*. (2017)^60^	Old (30, F)	80 ± 2	VL	Hip fracture	NR	NA	I, II: ↓	*I, II*: ↔	*I, II*: ↓	*I, II*: ↔
Farup *et al*. (2016)^61^	Young (32, NR)	46 ± 1	VL	Multiple sclerosis	NR	NA	Mixed, I, II: ↓	*I, II*: ↔	NM	*I, II*: ↔
Shao *et al*. (2020)^62^	Young (12, M/W)	14 ± 4	Bilateral thoracic multifidus	Idiopathic scoliosis	NR	NA	Mixed, I: ↓ *II*: ↔	Mixed, I, II: ↓	NM	Mixed, I, II: ↓
Verdijk *et al*. (2012)^63^	Young (8, M)	31 ± 3	VL	Spinal cord injury	9 years	NA	I, II: ↓	*I*: ↔;*II*: ↓	*I, II*: ↔	*I*: ↔; *II*: ↓
D'Souza *et al*. (2016)^64^	Young (11, M)	20 ± 2	VL	Type 1 diabetes	NR	NA	NM	NM	NM	Mixed: ↓

↑, significantly higher compared with control values; ↓, significantly lower compared with control values; ↔, no difference between experiment and control values; ACL, anterior cruciate ligament; COPD, chronic obstructive pulmonary patients; CP, cerebral palsy; I, Type I muscle fibres; II, Type II muscle fibres; M, men; M/W, men and women combined; Mixed, mixed muscle fibre type; NA, not applicable; NM, not measured; NR, not reported; SAR, sarcopenic patients; VL, vastus lateralis; W, women.

^a^ This study is performed in both muscle cross section and single muscle fibre.

^b^ This study is performed in single muscle fibre.

**Table 2:** The effect of ageing on myonuclear content and domain size and satellite cell content in humans

Author	Age, years (number)	Gender	Muscle	Muscle fibre size	Myonuclear content	Myonuclear domain	SC number
Vassilopoulos *et al*. (1977)^68^	12–30 (6) vs. 60–71 (6)	M/W	VL	Mixed: ↔	Mixed: ↔	NM	NM
Manta *et al*. (1987)^69^	17–30 (4) vs. >60 (7)	M/W	VL	Mixed: ↓	Mixed: ↔	Mixed: ↑	NM
Hikida *et al*. (1998)^70^	17–26 (7) vs. 59–71 (8)	M	VL	Mixed, *I, II*,: ↓	Mixed: ↔	NM	Mixed: ↔
Roth *et al*. (2000)^71^	22–28 (7) vs. 66–72 (8) 25–27 (7) vs. 64–71 (7)	M W	VL	NM	NM	NM	Mixed: ↔ Mixed: ↔
Renault *et al*. (2002)^72^	22–24 (6) vs. 70–78 (6)	M/W	Biceps Masseter	NM	Mixed: ↔ Mixed: ↔	NM	Mixed: ↓ Mixed: ↓
Sajko *et al*. (2002)^73^	24–38 (4) vs. 67–73 (6)	M	VL	NM	Mixed: ↓	NM	Mixed: ↓
Kadi *et al*. (2004)^74^	23–29 (15) vs. 70–78 (13) 20–26 (16) vs. 73–79 (14)	M W	VL	NM	Mixed: ↑ Mixed: ↑	NM	Mixed: ↓ Mixed: ↓
Sajko *et al*. (2004)^75^	26–30 (6) vs. 69–71 (6)	M	VL	NM	NM	NM	Mixed: ↓
Dreyer *et al*. (2006)^76^	21–35 (10) vs. >60 (9)	M	VL	*I*: ↔, *II*: ↓	Mixed: ↔	NM	Mixed: ↔
Petrella *et al*. (2006)^77^	20–35 (15) vs. 60–75 (13) 20–35 (16) vs. 60–75 (14)	M W	VL	Mixed: ↔ Mixed: ↔	Mixed: ↔ Mixed: ↔	Mixed: ↔ Mixed: ↔	Mixed: ↔ Mixed: ↔
Mohamed *et al*. (2007)^78^	24–50 (7) vs 65–81 (9)	NR	Triceps	Mixed: ↓	NM	NM	Mixed: ↓
Verdijk *et al*. (2007)^79^	19–21 (8) vs. 69–71 (8)	M	VL	*I*: ↔, *II*: ↓	*I, II*: ↑	*I, II*: ↓	*I*: ↔, *II*: ↓
Cristea *et al*. (2010)^a^, ^80^	21–32 (6) vs. 72–96 (9) 24–32 (6) vs. 65–96 (9)	M W	VL	*I*: ↑, *II*: ↓ *I*: ↑, *II*: ↓	*I*: ↑, *II*: ↔ *I*: ↑, *II*: ↔	*I*: ↑, *II*: ↓ *I, II*: ↔	NM
McKay *et al*. (2012)^81^	18–24 (9) vs. 66–74 (9)	M	VL	*I*: ↔, *II*: ↓	NM	*I*: ↔, *II*: ↓	Mixed, II: ↓, *I*: ↔
Verdijk *et al*. (2012)^63^	28–34 (8) vs. 73–77 (8)	M	VL	*I*: ↔, *II*: ↓	*I*: ↔, *II*: ↓	*I*: ↔, *II*: ↔	*I*: ↔, *II*: ↓
Walker *et al*. (2012)^82^	25–29 (5) vs. 68–72 (6) 25–29 (5) vs. 68–72 (6)	M W	VL	*I*: ↔, *II*: ↓ *I*: ↔, *II*: ↓	Mixed: ↔ Mixed: ↔	Mixed: ↓ Mixed: ↔	Mixed: ↔ Mixed: ↓
Suetta *et al*. (2013)^42^	21–30 (11) vs. 61–74 (9)	M	VL	*I, II*: ↔	NM	NM	*I, II*: ↔
McKay *et al*. (2014)^83^	21–27 (12) vs. 62–70 (12)	M	VL	*I, II*: ↔	*I, II*: ↔	*I, II*: ↔	*I, II*: ↔
Snijders *et al*. (2014)^65^	21–23 (10) vs. 72–74 (10)	M	VL	*I*: ↔, *II*: ↓	NM	NM	*I*: ↔, *II*: ↓
Verdijk *et al*. (2014)^84^	18–49 (50) vs. ≥70 (49)	M	VL	*I*: ↔, *II*: ↓	*I*: ↔, *II*: ↓	*I, II*: ↔	*I*: ↔, *II*: ↓
Verdijk *et al*. (2016)^85^	24–28 (14) vs. 71–73 (16)	M	VL	*I*: ↔, *II*: ↓	*I, II*: ↓	NM	*I*: ↔, *II*: ↓
Nederveen *et al*. (2016)^86^	21–24 (23) vs. 63–71 (22)	M	VL	*I*: ↔, *II*: ↓	NM	NM	*I*: ↔, *II*: ↓
Kramer *et al*. (2017)^60^	18–25 (15) vs. ≥65 (15)	W	VL	*I*: ↔, *II*: ↓	*I*: ↔, *II*: ↓	*I*: ↑, *II*: ↔	*I, II*: ↔
Kelly *et al*. (2018)^87^	22–30 (27) vs. 62–70 (91)	M	VL	*I*: ↔, *II*: ↓	*I, II*: ↔	*I*: ↔, *II*: ↓	*I, II*: ↔
Reidy *et al*. (2018)^46^	18–35 (14) vs. 60–75 (9)	M/W	VL	Mixed: ↔	NM	NM	Mixed, *I, II*: ↔
Karlsen *et al*. (2019)^88^	19–23 (9) vs. 70–84 (18)	M	VL	*I*: ↔, *II*: ↓	*I*: ↔, *II*: ↓	*I*: ↔, *II*: ↓	*I*: ↔, *II*: ↓
Naro *et al*. (2019)^b^, ^89^	22–28 (6) vs. 81–96 (6)	M/W	VL	*I, II*: ↔	*I, II*: ↔	*I, II*: ↔	NM
Karlsen *et al*. (2020)^90^	22–28 (7) vs. 63–71 (19)	M	VL	*I*: ↔, *II*: ↓	NM	NM	Mixed, I: ↔, *II*: ↓
Perez *et al*. (2021)^91^	20–24 (6) vs. 65–78 (11)	M/W	VL	NM	NM	NM	Mixed: ↓

↑, significantly higher compared with control values; ↓, significantly lower compared with control values; ↔, no difference between experiment and control values; I, Type I muscle fibres; II, Type II muscle fibres; M, men; M/W, men and women combined; Mixed, mixed muscle fibre type; NM, not measured; VL, vastus lateralis; W, women.

^a^ This study is performed in single muscle fibre.

^b^ This study is performed in both muscle cross‐section and single muscle fibre.

**Table 3:** The effect of hypertrophy or atrophy on myonuclear content and domain size and satellite cell content in rodents

Author	Specie (sex)	Muscle	Hypertrophy/atrophy model	Training/atrophy duration	Detraining duration	Myonuclear content	Satellite cell number
Bruusgaard *et al*. (2010)^a^, ^16^	NMRI mice (F)	EDL	Synergist ablation	14 d	2/8 wk denervation	***In vivo*** Training: ↑, Detraining: ↔ ***Ex vivo*** Training: ↑, Detraining: ↔	NM
Lee *et al*. (2018)^a^, ^18^	Sprague–Dawley rats (F)	FHL	Weight loaded‐ladder climbing	8 wk	20 wk	Training: ↑, Detraining: ↔	NM
Dungan *et al*. (2019)^b^, ^21^	C57BL/6J mice (F)	Plan	Weighted wheel running	8 wk	12 wk	**Single muscle fibre** Training: *↑*, Detraining: ↓ **Muscle cross section** Training: *↑, D*etraining: ↓	**Muscle cross section** Training: *↑* Detraining: *↔*
Murach *et al*. (2020)^b^, ^22^	C57BL/6J mice (F)	Sol, Gas, Plan	Weighted wheel running	8 wk	24 wk	**Single muscle fibre** Training: Sol, Gas: *↑* Detraining: Sol: ↔, Gas: ↓ **Muscle cross section** Training: Sol: *↑,* Gas: *↔* Detraining: Sol, Gas: *↔*	NM
Eftestøl *et al*. (2021)^92^	Sprague–Dawley rats (M)	Sol	Climbing	5 wk	10 wk	Training: *↑* Detraining: *↑*	NM
Hyatt *et al*. (2003)^S1^	Sprague–Dawley rats (F)	MG, TA	Denervation Spinal cord transection	3, 14, 28 d	NA	NM	**Denervation** 3 d in MG, TA: *↔* 14, 28 d in MG, TA: *↑* **Spinal cord transection** 3, 14, 28 d in MG: *↔* 3, 28 d in TA: *↔* 14 d in TA: *↑*
Kasper *et al*. (1996a)^a^ ^S69^	Sprague–Dawley rats (F)	Gas, TA	Suspension Space flight	5.4 d	NA	**Suspension:** Gas, TA: *↔* **Space flight:** Gas, TA: *↑*	NM
Bruusgaard *et al*. (2008)^b^, ^17^	NMRI mice (F)	EDL, Sol	Suspension Denervation TTX blockade	3, 7, 14, and 21 d	NA	**Single muscle fibre** **Suspension**: 14 d in EDL: *↔* **Denervation**: 7, 14, and 21 d in EDL: *↔,* 14 and 21 d in Sol: *↔* **TTX blockade**: 14 and 21 d in EDL: *↔* **Muscle cross‐section** **Denervation**: 3, 7, 14, and 21 d in EDL& Sol: *↔*	NM
Ontell (1974)^a^ ^S2^	Wistar rats (M)	EDL	Denervation	2 and 3 wk	NA	2 and 3 wk: *↔*	2 and 3 wk: *↑*
Cardasis & Cooper (1975)^a^ ^S3^	Princeton‐Rockefeller mice (M)	Gas	Denervation	1, 2, 3, 7, 14, 18, and 28 d	NA	1, 2, 3, 7, 14, 18, and 28 d: *↔*	NM
Snow (1983)^S4^	C57BL/6 mice (M/F)	EDL, Sol	Denervation	3, 7, 14, 23, 30, 45, and 65 d	NA	NM	3, 7, 14, 23, and 65 d in EDL & Sol: ↔, 30d in EDL: ↑, 30 and 45 d in Sol: *↑*
Maltin *et al*. (1992)^S5^	Hooded Lister rats (M)	Sol	Denervation	4 d	NA	↓	↓
Irintchev *et al*. (1994)^S6^	CBA/J and Balb/c mice	Sol	Denervation	5 and 7 d	NA	NM	5 and 7 d: *↑*
Allen *et al*. (1995)^a^ ^S7^	**Cats** (F)	Sol	Denervation	6 mos	NA	Sol: *↔*	NM
Viguie *et al*. (1997)^a^ ^S8^	WI/HicksCar rats (M)	EDL	Denervation	2, 4, and 7 mos	NA	2, 4, and 7 mos: ↓	2 mos: *↑* 4 mos: *↔* 7 mos: ↓
Dupont‐Versteegden *et al*. (1999)^25^	Sprague–Dawley rats (F)	Sol	Denervation	10 d	NA	↓	NM
Milanic *et al*. (1999)^a^ ^S9^	Wistar rats (F)	Sternomastoideus	Denervation	4 and 7 d	NA	4 and 7 d: *↔*	NM
Dupont‐Versteegden *et al*. (2000)^S10^	Sprague–Dawley rats (F)	Plan, Sol	Denervation	8 wk	NA	Plan: *↔,* Sol: ↓	NM
Schmalbruch *et al*. (2000)^S11^	Wistar rats (M)	EDL, Sol	Denervation	10 wk	NA	EDL: ↓, Sol: ↓	NM
Dedkov *et al*. (2001)^S12^	WI/HicksCar rats (M)	EDL	Denervation	25 mos	NA	↓	↓
Nnodim (2001)^a^ ^S13^	WI/HicksCar rats (M)	Levator ani	Denervation	8 wk	NA	*↔*	*↑*
Wada *et al*. (2002)^a^ ^S14^	ICR mice (M)	Plan	Denervation	5, 10, and 120 d	NA	3 weeks old (5, 10 d): ↓ 4 months old (10, 120 d): *↔*	NM
Dedkov *et al*. (2003)^S15^	Young and old WI/HicksCar rats (M)	TA	Denervation	2 mos	NA	NM	Young: *↑,* Old: *↑*
Roy *et al*. (2005)^a^ ^S16^	Sprague–Dawley rats (F)	MG, TA	Denervation	4 and 60 d	NA	4 d in MG: *↔* 60 d in MG: ↓ 4 and 60 d in TA: *↔*	NM
Zhong *et al*. (2005)^a^ ^S17^	Sprague–Dawley rats (F)	Sol	Denervation	4 and 60 d	NA	4 d: ↓ 60 d: *↔*	NM
Aravamudan *et al*. (2006)^a^ ^S18^	Sprague–Dawley rats (M)	Diaphragm	Denervation	14 d	NA	*↔*	NM
Van Der Merr *et al*. (2011)^S19^	Wistar rats (M)	Gas	Denervation	1, 2, and 4 wk	NA	**5‐month‐age rats** 1, 2, 4 wk: *↔* **25‐month‐age rats** 1, 2, and 4 wk: *↔*	**5‐month‐age rats** 1 and 2 wk: *↔,* 4 wk: *↑* **25‐month‐age rats** 1 wk: *↑,* 2 and 4 wk: *↔*
Liu *et al*. (2015)^S20^	C57BL/6 mice (M)	TA	Denervation	6 wk	NA	NM	*↑*
Aguera *et al*. (2019)^S21^	Wistar rats (M)	Sol	Denervation	10 d	NA	NM	*↑*
Choi *et al*. (2020)^a^ ^S22^	TA	Denervation	Denervation	7 d	NA	NM	↓
Hansson *et al*. (2020)^a^, ^1^	NMRI mice (F)	EDL	Denervation	14 d	NA	*↔*	NM
Xing *et al*. (2020)^S23^	Sprague–Dawley rats (M)	Gas	Denervation	2, 4, and 6 wk	NA	2, 4, and 6 wk: ↓	2, 4, and 6 wk: *↔*
Wong *et al*. (2021)^S24^	C57BL/6 mice (M)	TA	Denervation	3,6, and 12 mos	NA	NM	3 and 6 mos: *↑,* 12 mos: *↔*
Darr *et al*. (1989)^b^ ^S26^	Sprague Dawley rats (M)	EDL, Sol	Suspension	3, 10, 20, and 30 d	NA	**Single muscle fibre** 3 d in Sol: *↔* 10, 20, 30 d in Sol: ↓ 3, 10, and 30 d in EDL: *↔* 20 d in EDL: ↓ **Muscle cross‐section** 3 d in Sol: *↔* 10, 20, 30 d in Sol: ↓ 3, 10, 20, and 30 d in EDL: *↔*	**Single muscle fibre** 3,10, 20, and 30 d in Sol: ↓ 3 d in EDL: ↓ 10, 20, and 30 d in EDL: *↔* **Muscle cross‐section** 3 d in Sol, EDL: ↓ 10, 20, and 30 d in Sol: *↔* 10, 20, and 30 d in EDL:*↔*
Kasper *et al*. (1996b)^a^ ^S27^	Wistar rats (F)	Sol, Plan	Suspension	28 d	NA	Sol: *↑,* Plan: *↔*	NM
Allen *et al*. (1997)^a^ ^S28^	Sprague–Dawley rats (F)	Sol	Suspension	14 d	NA	↓	NM
Mozdziak *et al*. (2000)^a^ ^S29^	Sprague–Dawley rats (M)	Sol	Suspension	28 d	NA	↓	NM
Mitchell *et al*. (2001)^a^ ^S30^	BALB/c mice (F)	Sol	Suspension	14 d	NA	↓	NM
Yamazaki (2003)^S31^	Wistar rats (M)	Sol	Suspension	14 d	NA	↓	NM
Mitchell and Pavlath (2004)^S32^	C57BL/6 mice (F)	Sol	Suspension	14 d	NA	↓	NM
Leeuwenburgh *et al*. (2005)^24^	Fischer 344 Norway rats(M)	Sol	Suspension	14 d	NA	6 mos: ↓, 32 mos: *↔*	NM
Ferreira *et al*. (2006)^S33^	Charles River mice (M)	Gas	Suspension	6,12,24, 48, and 72 h and 1 wk	NA	NM	6, 12, 24, and 48 h: *↑* 72 h, 1 wk: ↓
Wang *et al*. (2006)^S34^	Wistar rats (M)	Sol	Suspension	16 d	NA	↓	↓
Kawano *et al*. (2007)^a^ ^S35^	Wistar rats (M)	Sol	Suspension	14 d	NA	↓	NM
Kawano *et al*. (2008)^a^ ^S36^	Wistar rats (M/F)	Sol	Suspension	3 mos	NA	↓	↓
Oishi *et al*. (2008)^b^ ^S37^	Wistar rats (M)	Sol	Suspension	14 d	NA	↓	NM
Tarakina *et al*. (2008)^S38^	Wistar rats (M)	Sol	Suspension	14 d	NA	↓	↓
Matsuba *et al*. (2009)^S39^	C57BL/6 mice (M)	Sol	Suspension	14, 28, and 42 d	NA	14, 28, and 42 d: *↔*	14, 28, and 42 d: ↓
Kartashkina *et al*. (2010)^S40^	Wistar rats (M)	Sol	Suspension	14 d	NA	↓	↓
Zhang *et al*. (2010)^S41^	Wistar rats (M)	EDL, Sol	Suspension	28 d	NA	EDL: *↔,* Sol: ↓	EDL, Sol: ↓
Kachaeva *et al*. (2011)^S42^	Wistar rats (M)	Sol	Suspension	14 d	NA	↓	↓
Ohira *et al*. (2011)^S43^	Wistar rats (M)	Adductor longus	Suspension	16 and 32 d	NA	16 and 32 d: ↓	16 and 32 d: *↔*
Teixeira *et al*. (2011)^S44^	Charles River mice (M)	Sol	Suspension	1, 2, 3, and 8 d	NA	1 d: ↔, 2, 3, and 8 d: ↓	NM
Bruusgaard *et al*. (2012)^15^	Wistar rats (F)	Sol	Suspension	2, 4, and 14 d	NA	*↔*	NM
Jackson *et al*. (2012)^a^ ^S25^	Pax7‐DTA mice (F)	Sol	Suspension	14 d	NA	*↔*	NM
Guo *et al*. (2012)^23^	BALB/c mice (M)	Sol	Suspension	14 d	NA	NM	↓
Lomonosova *et al*. (2012)^S45^	Wistar rats (M)	Sol	Suspension	14 d	NA	NM	↓
Zushi *et al*. (2012)^S46^	Wistar rats (M)	Sol	Suspension	14 d	NA	↓	NM
Itoh *et al*. (2014)^S47^	ICR mice (M)	Sol	Suspension	14 d	NA	↓	NM
Park *et al*. (2014)^S48^	C57BL/6 mice (F)	Sol	Suspension	7 d	NA	NM	*↔*
Babcock *et al*. (2015)^S49^	Wistar rats (M)	TA	Suspension	10 d	NA	↓	↓
Ohira *et al*. (2015)^a^ ^S50^	Osteopetrotic mice (M)	Sol	Suspension	10 d	NA	In +/+, +/*op and op*/*op*: ↓	In +/+, +/*op and op*/*op*: ↓
Nakanish *et al*. (2016)^S51^	Wistar rats (F)	Sol	Suspension	7 d	NA	↓	↓
Itoh *et al*. (2017)^S52^	ICR mice (M)	Sol	Suspension	14 d	NA	*↔*	NM
Anderson *et al*. (2018)^S53^	C57BL/6 mice (M/F)	Gas	Suspension	18 d	NA	NM	↓
Brooks *et al*. (2018)^66^	C57BL/6 mice (M/F)	Gas	Suspension	14 d	NA	↓	*↔*
Miller *et al*. (2018)^S54^	Norway–F344 rats (M)	Gas	Suspension	14 d	NA	*↔*	↓
Kneppers *et al*. (2019)^S55^	C57BL/6 mice (M)	Gas	Suspension	14 d	NA	↓	*↔*
Nakanishi *et al*. (2021)^S56^	Wistar rats (F)	Sol	Suspension	14 d	NA	NM	↓
Petrocelli *et al*. (2021)^S57^	C57BL/6 mice (M)	Gas, Sol	Suspension	14 d	NA	NM	Gas, Sol: *↔*
Smith *et al*. (2000)^S58^	Californian rabbits (F)	Sol	Immobilization	2 and 6 d	NA	2 d: *↔, 6 d*: ↓	NM
Wanek and Snow (2000)^S59^	Sprague–Dawley rats (M/F)	Sol	Immobilization	2, 4, and 8–10 wk	NA	NM	2 and 4 wk:↔, 8–10 wk: ↓
Ye *et al*. (2013)^S60^	C57BL/6 mice (F)	Sol	Immobilization	14 d	NA	NM	↓
Matsumoto *et al*. (2014)^S61^	Wistar rats (M)	Gas	Immobilization	4 wk	NA	↓	NM
Li *et al*. (2016)^28^	Wistar rats (M)	Sol	Immobilization	14 d	NA	NM	↓
Guitart *et al*. (2018)^S62^	C57BL/6 mice (F)	Gas, Sol	Immobilization	7 d	NA	NM	Gas: ↓, Sol: ↓
Usuki *et al*. (2019)^S63^	Wistar rats (M)	Sol	Immobilization	7 d	NA	NM	↓
Suzuki *et al*. (2020)^S64^	Sprague–Dawley rats (M)	Plan, Sol	Immobilization	7 d	NA	Plan, Sol: *↔*	NM
Zazula *et al*. (2020)^S65^	Wistar rats (M)	TA	Immobilization	7 d	NA	↓	NM
Honda *et al*. (2021)^S66^	Wistar rats (M)	Sol	Immobilization	14 d	NA	↓	NM
Allen *et al*. (1996)^a^ ^S67^	Sprague–Dawley rats (M)	Sol	Space flight	14 d	NA	↓	NM
Hikida *et al*. (1997)^S68^	Fisher 344 rats (M)	Sol	Space flight	10 d	NA	↓	NM
Sandonà *et al*. (2012)^67^	C57BL/10J mice (M)	EDL, Sol	Space flight	91 d	NA	EDL: *↔,* Sol: ↓	NM
Radugina *et al*. (2018)^27^	C57BL/6 mice (M)	Quadriceps	Space flight	30 d	NA	↓	NM
McClung *et al*. (2006)^S70^	Sprague–Dawley rats (F)	Diaphragm	Mechanical ventilation	12 h	NA	↓	NM

↑, significantly higher compared with control values; ↓, significantly lower compared with control values; ↔, no difference between experiment and control values; EDL, extensor digitorum longus; F, female; FHL, flexor hallucis longus; Gas, gastrocnemius muscle; ICR, Institute of Cancer Research (ICR) mice (Japan SLC, Shizuoka, Japan); M, male; M/F, male and female combined; MG, medial gastrocnemius; NA, not applicable; NM, not measured; Plan, plantaris muscle; Sol, soleus muscle; TA, tibialis anterior muscle.

^a^ This study is performed in single muscle fibre.

^b^ This study is performed in both muscle cross‐section and single muscle fibre.

### Data analysis

All data analyses were conducted using Review Manager Software (RevMan 5.3, Cochrane Collaboration, Copenhagen, Denmark) as previously described in detail by us.^34^ For instance, when data was only available in a graphic format, we used WebPlotDigitizer software to extract quantitative data from the figure. Results were expressed as standardized mean difference (SMD) and 95% confidence intervals (CI) when the outcome is measured in different ways; otherwise, the mean difference (MD) and 95% CI were calculated.^31^, ^34^ When there was a sufficient number of studies, subgroup analysis was performed on muscle type, atrophy model, atrophy duration, and hypertrophy percentage in the animal model and on age (young and old) and atrophy model in human subjects. To evaluate and ensure the robustness of the results, sensitivity analysis was carried out by removing studies from the meta‐analysis. Sensitivity analysis showed that no results were affected by any study (data not shown). Finally, funnel plots with *Egger* weighted regression test were used for assessing publication bias using STATA version 16.

## Results

### Evidence from human studies

#### Skeletal muscle responses to hypertrophy

Four reports involving 117 participants assessed the response of skeletal muscle (*vastus lateralis*) to resistance training followed by a period of detraining.^29^, ^30^, ^35^, ^36^ Resistance training duration ranged from 10 to 24 weeks in these studies. However, detraining duration ranged from 12 to 48 weeks. Currently, the general consensus is that myonuclear content tends to be lower in older adults (≥60 year) compared with young adults (18–55 year).^37^ Thus, we performed a subgroup analysis to clarify the effects of an episode of overload hypertrophy and subsequent disuse atrophy on the present review outcomes in terms of the different age categories. The details of the included studies are shown in *Table* *1*.

##### Myofibre size following training and detraining

Resistance training significantly increased cross‐sectional area (CSA) compared with baseline values (mean: MD = 650.32, 95% CI 355.30–945.34, *P* = 0.0001; Type I: MD = 470.83, 95% CI 168.29–773.37, *P* = 0.002; Type II: MD = 723.93, 95% CI 358.02–1089.84, *P* = 0.0001; *Figure* *S2* *A–* *S2* *C*). Further, CSA after a detraining period following resistance training returned to the pre‐training values (mean: MD = 83.46, 95% CI −649.41 to 816.32, *P* = 0.82; Type I: MD = 104.39, 95% CI −604.64 to 813.23, *P* = 0.77; Type II: MD = 190.74, 95% CI −882.92 to 1264.40, *P* = 0.73; *Figure* *S2* *D–* *S2* *F*). Subgroup analysis in mixed and Type II fibres showed no statistically significant difference between young and old adults after training and detraining periods (mixed: *P* = 0.50 and *P* = 0.20; Type II: *P* = 0.97 and *P* = 0.31, respectively). Further, subgroup analysis showed that the reduction of Type I fibre CSA of young adults was significantly higher following a detraining period than old subjects (*P* = 0.03) (*Figure* *S2* *A–* *S2* *F*).

##### Myonuclear content following training and detraining

Resistance training significantly increased myonuclear content in mixed and Type II fibres compared with baseline values (mean: MD = 0.12, 95% CI 0.00–0.23, *P* = 0.04; Type I: MD = 0.04, 95% CI −0.08 to 0.15, *P* = 0.55; Type II: MD = 0.23, 95% CI 0.07–0.40, *P* = 0.006; *Figure* *S2* *G–* *S2* *I*). Compared with pre‐training, there was a significant difference in myonuclear content after a detraining period (mean: MD = −0.14, 95% CI −0.26 to −0.02, *P* = 0.02; Type I: MD = −0.14, 95% CI −0.28 to −0.0, *P* = 0.05; Type II: MD = −0.23, 95% CI −0.37 to −0.10, *P* = 0.0009; *Figure* *S2* *J–* *S2* *L*), indicating that myonuclear content after a detraining period was less than the baseline. Subgroup analysis showed no statistically significant difference between young and old adults after training and detraining periods (mixed: *P* = 0.56 and *P* = 0.73; Type I: *P* = 0.42 and *P* = 0.86; Type II: *P* = 0.37 and *P* = 0.73; respectively; *Figure* *S2* *G–* *S2* *L*).

##### Myonuclear content in single muscle fibre following training and detraining

A single report with 19 participants assessed myonuclear content in single muscle fibre using 44–57 fibres from each biopsy sample.^29^ This study reported no change in myonuclear content in response to resistance training (i.e. +5%) and after detraining (i.e. +3%).

##### Myonuclear domain following training and detraining

Resistance training significantly increased myonuclear domain (MND) only in mixed fibres compared with baseline values (mean: MD = 110.91, 95% CI 24.93–196.89, *P* = 0.01; Type I: MD = 5.67, 95% CI −133.51 to 144.85, *P* = 0.94; Type II: MD = 73.87, 95% CI −62.35 to 210.09, *P* = 0.29; *Figure* *S2* *M–* *S2* *O*). Moreover, MND after a detraining period returned to pre‐training levels (mean: MD = 43.16, 95% CI −42.14 to 128.47, *P* = 0.32; Type I: MD = −9.26, 95% CI −166.29 to 147.77, *P* = 0.91; Type II: MD = 55.98, 95% CI −138.18 to 250.14, *P* = 0.57; *Figure* *S2* *P–* *S2* *R*). Subgroup analysis in mixed fibres showed no statistically significant difference between young and old adults after training and detraining periods (*P* = 0.06 and *P* = 0.33, respectively). The number of studies was too small to permit subgroup analyses of Type I or Type II fibres (*Figure* *S2* *M–* *S2* *R*).

##### Satellite cell number following training and detraining

Three studies involving 94 participants assessed SC abundance.^30^, ^35^, ^36^ Resistance training significantly increased SC abundance in mixed and Type II fibres compared to baseline values (mean: SMD = 0.75, 95% CI 0.33–1.18, *P* = 0.0005; Type I: SMD = 0.36, 95% CI −0.14 to 0.85, *P* = 0.16; Type II: SMD = 0.81, 95% CI 0.30–1.32, *P* = 0.002; *Figure* *S2* *S–* *S2* *U*). Additionally, SC abundance after a detraining period returned to pre‐training levels (mean: SMD = 0.16, 95% CI −0.32 to 0.64, *P* = 0.52; Type I: SMD = −0.01, 95% CI −0.66 to 0.65, *P* = 0.99; Type II: SMD = 0.09, 95% CI −0.57 to 0.74, *P* = 0.79; *Figure* *S2* *W–* *S2* *Y*). Subgroup analysis in mixed fibres showed no statistically significant difference between young and old adults after training and detraining periods (*P* = 0.29 and *P* = 0.58, respectively). The number of studies was too small to permit subgroup analyses of Type I or Type II fibres (*Figure* *S2* *S–* *S2* *Y*).

#### Skeletal muscle responses to atrophy

Twenty‐nine studies assessed skeletal muscle growth in whole muscle cross section in response to leg immobilization,^38^, ^39^, ^40^, ^41^, ^42^ bed rest,^19^, ^43^, ^44^, ^45^, ^46^ step reduction,^47^, ^48^ space flight,^20^ and patients suffering from cerebral palsy,^49^, ^50^, ^51^ chronic obstructive pulmonary disease (COPD),^52^, ^53^, ^54^, ^55^ anterior ligament reconstruction,^56^, ^57^, ^58^, fully sedating ICU patients^59^ hip fracture,^60^ multiple sclerosis,^61^ adolescent idiopathic scoliosis,^62^ spinal cord injury,^63^ and Type1 diabetes.^64^ The details of the included studies are shown in *Table* *1*. We performed subgroup analyses to determine the potential impact that differences in the age of the participants (old vs. young), the duration of the intervention (≤5 days, 7–14 days, 20–30 days, and ≥60 days), and the model of atrophy used had on the atrophic response.

##### Myofibre size following atrophy

Analysis of 19 studies involving 460 participants^19^, ^39^, ^41^, ^42^, ^43^, ^45^, ^46^, ^47^, ^50^, ^52^, ^53^, ^54^, ^55^, ^56^, ^59^, ^60^, ^62^, ^63^, ^65^ found there was lower skeletal muscle CSA following the aforementioned (‘Skeletal muscle responses to atrophy’ section) interventions (mixed: MD = −497.24, 95% CI −734.13 to −260.35, *P* = 0.0001; Type I: MD = −743.63, 95% CI −1059.28 to −427.98, *P* = 0.00001; Type II: MD = −908.11, 95% CI −1268.67 to −547.54, *P* = 0.00001; *Figure* *S3* *A–* *S3* *C*). Subgroup analysis showed no statistically significant difference between young and old adults for CSA of mixed, Type I, and Type II fibres in response to atrophy (*P* = 0.52, *P* = 0.93, and *P* = 0.60, respectively). Stratifying studies based on the duration of the intervention period found that myofibre CSA was decreased after 7 days in different atrophy models (mixed: MD = −914.33, 95% CI −1528.91 to −299.75, *P* = 0.004; Type I: MD = −710.72, 95% CI −1217.05 to −204.38, *P* = 0.006; Type II: MD = −1126.26, 95% CI −1618.85 to −633.68, *P* = 0.00001; *Figure* *S3* *D–* *S3* *F*). Subgroup analysis that stratified studies based on the model of atrophy showed that bed rest, COPD, idiopathic scoliosis, and hip fracture induced a significant decrease in fibre CSA (*Figure* *S3* *G–* *S3* *I*).

##### Myonuclear content following atrophy

Analysis of 13 studies involving 260 participants^19^, ^39^, ^40^, ^41^, ^43^, ^44^, ^45^, ^47^, ^50^, ^59^, ^60^, ^61^, ^62^ found lower myonuclear content in response to skeletal muscle atrophy (mean: MD = −11, 95% CI −0.19 to −0.03, *P* = 0.005; Type I: MD = −0.09, 95% CI −0.17 to −0.00, *P* = 0.04; Type II: MD = −0.13, 95% CI −0.22 to −0.05, *P* = 0.003; *Figure* *S3* *J–* *S3* *L*). Interestingly, subgroup analysis showed myonuclear content in mixed, Type I, and Type II fibre only decreased in young adults and not in old adults who experienced atrophy (old adults: *P* = 0.61, *P* = 0.58, and *P* = 0.77, respectively). Subgroup analysis showed no difference between different period of interventions in mixed, Type I, and Type II fibres (*P* = 0.69, *P* = 0.81, and *P* = 0.64, respectively; *Figure* *S3* *M–* *S3* *O*). Stratifying studies based on the model of atrophy showed that bed rest, idiopathic scoliosis, and cerebral palsy induced a significant decrease in myonuclear content (*Figure* *S3* *P–* *S3* *R*).

##### Myonuclear content in single muscle fibre following atrophy

A single report with five astronauts assessed myonuclear content in single muscle fibre using 42–81 fibres from each biopsy sample before and after 11 days of space flight.^20^ This study reported no change in the myonuclear content of Type I fibres, whereas lower myonuclear content was found in Type II fibres.

##### Myonuclear domain following atrophy

Analysis of 10 studies involving 202 participants^19^, ^39^, ^40^, ^41^, ^43^, ^47^, ^50^, ^59^, ^60^, ^63^ found a significant decrease in MND in response to skeletal muscle atrophy (mean: MD = −1.92, 95% CI −2.72 to −1.12, *P* = 0.00001; Type I: MD = −0.65, 95% CI −0.97 to −0.32, *P* = 0.0001; Type II: MD = −0.72, 95% CI −1.03 to −0.40, *P* = 0.0001; *Figure* *S3* *S–* *S3* *W*). The results from a single study with five astronauts showed lower MND in single muscle fibres after 11 days of space flight.^20^ Subgroup analysis showed no difference between the reduction of MND in mixed, Type I, and Type II fibres in old and young adults and different periods of intervention (*Figure* *S3* *X–* *S3* *Z*). Stratifying studies based on the model of atrophy showed that leg immobilization, step reduction, cerebral palsy, and hip fracture induced a significant decrease in myonuclear content (*Figure S3A1* *–* *S3C1*).

##### Satellite cell number following atrophy

Analysis from 24 studies involving 611 participants^19^, ^38^, ^39^, ^41^, ^45^, ^46^, ^47^, ^50^, ^51^, ^52^, ^53^, ^54^, ^56^, ^57^, ^58^, ^59^, ^60^, ^61^, ^64^, ^66^, ^67^ found there was lower SC abundance in response to skeletal muscle atrophy (mean: SMD = −0.49, 95% CI −0.77 to −0.22, *P* = 0.0005; Type I: SMD = −0.20, 95% CI −0.59 to 0.20, *P* = 0.33; Type II: SMD = −0.37, 95% CI −0.71 to −0.02, *P* = 0.04; *Figure S3D1* *–* *S3F1*). In agreement with changes in myonuclear content, subgroup analysis showed SC content in mixed, Type I, and Type II fibre only decreased in young adults and not in old adults who experienced atrophy (old adults: *P* = 0.07, *P* = 0.76, and *P* = 0.35, respectively). Stratifying studies based on duration of the intervention period found that SC content was decreased after 60 days (mixed: MD = −0.85, 95% CI −1.61 to −0.09, *P* = 0.03; Type I: MD = −0.87, 95% CI −0.48 to −0.43, *P* = 0.01; Type II: MD = −1.02, 95% CI −1.61 to −0.44, *P* = 0.0006; *Figure S3G1* *–* *S3I1*). Stratifying studies based on the model of atrophy showed that bed rest, cerebral palsy, idiopathic scoliosis, and ACL injury induced a significant decrease in SC content (*Figure S3J1* *–* *S3L1*).

#### Skeletal muscle responses in ageing compared with young adults

Next, we assessed the impact of ageing on myonuclear content, MND, and SC abundance. Twenty‐nine studies measured the aforementioned skeletal muscle characteristics in young and old adults.^42^, ^60^, ^63^, ^65^, ^68^, ^69^, ^70^, ^71^, ^72^, ^73^, ^74^, ^75^, ^76^, ^77^, ^78^, ^79^, ^80^, ^81^, ^82^, ^83^, ^84^, ^85^, ^86^, ^87^, ^88^, ^89^, ^90^, ^91^ The details of the included studies are shown in *Table* *2*.

##### Myofibre size following ageing

Analysis of 25 studies involving 724 participants^42^, ^46^, ^60^, ^63^, ^65^, ^68^, ^69^, ^70^, ^76^, ^77^, ^78^, ^79^, ^80^, ^81^, ^82^, ^83^, ^84^, ^85^, ^86^, ^87^, ^88^, ^89^, ^90^ found that except Type I fibres, CSA in mixed and Type II fibres decreased with ageing (mean: SMD = 0.91, 95% CI 0.25–1.56, *P* = 0.007; Type I: MD = −131.7, 95% CI −353.91 to 90.51, *P* = 0.25; Type II: MD = 1313.31, 95% CI 995.45–1631.16, *P* = 0.00001; *Figure* *S4* *A–* *S4* *C*).

##### Myonuclear content following ageing

Analysis of 17 studies involving 494 participants^60^, ^63^, ^68^, ^69^, ^70^, ^72^, ^74^, ^76^, ^77^, ^79^, ^82^, ^83^, ^84^, ^85^, ^86^, ^87^, ^88^ found no change in myonuclear content of mixed and Type I fibres with ageing (MD = −0.03, 95% CI −0.24 to 0.19, *P* = 0.8; MD = −0.01, 95% CI −0.31 to 0.29, *P* = 0.95; respectively; *Figure* *S4* *D* and *S4* *E*). A pooled analysis from eight studies involving 274 participants found lower myonuclear content in Type II fibres with ageing (MD = 0.47, 95% CI 0.09–0.85, *P* = 0.02; *Figure* *S4* *F*).

##### Myonuclear content in single muscle fibre following ageing

Two studies assessed muscle response to ageing at the single muscle fibre level. Cristea *et al*.^80^ in a separate analysis of men and women reported a significant increase in myonuclear content of Type I fibres with no change in Type II fibres. In another study, Naro *et al*.^89^ reported no change in myonuclear content and MND of Type I and II fibres.

##### Myonuclear domain following ageing

Analysis of 11 studies involving 346 participants^60^, ^63^, ^69^, ^77^, ^79^, ^81^, ^82^, ^83^, ^84^, ^87^, ^88^ found no change in MND of mixed and Type I fibres with ageing (MD = 236.01, 95% CI −11.78 to 483.79, *P* = 0.06; MD = −26.75, 95% CI −207.05 to 153.56, *P* = 0.77; respectively; *Figure* *S4* *G* and *S4* *H*). In contrast, there was lower MND in Type II fibres with ageing (MD = 296.19, 95% CI 109.08–483.29, *P* = 0.002; *Figure* *S4* *I*).

##### Satellite cell number following ageing

Analysis of 25 studies involving 717 participants^42^, ^46^, ^63^, ^65^, ^70^, ^71^, ^72^, ^73^, ^74^, ^75^, ^76^, ^77^, ^78^, ^79^, ^81^, ^82^, ^83^, ^84^, ^85^, ^86^, ^87^, ^88^, ^90^, ^91^ found lower SC abundance in mixed fibres with ageing (SMD = 0.78, 95% CI 0.37–1.19, *P* = 0.0002; *Figure* *S4* *J*). There was no change in SC content associated with Type I fibres, whereas SC content associated with Type II fibres was lower with ageing (SMD = 0.09, 95% CI −0.11 to 0.28, *P* = 0.38; SMD = 1.23, 95% CI 0.86–1.60, *P* = 0.00001; respectively; *Figure* *S4* *K* and *S4* *L*).

### Evidence from animal studies

#### Skeletal muscle responses to hypertrophy

Five studies assessed skeletal muscle growth in response to a hypertrophic stimulus induced by synergist ablation,^16^ weight loaded‐ladder climbing,^18^ climbing,^92^ or weighted wheel running^21^, ^22^ in *extensor digitorum longus* (EDL),^16^ *flexor hallucis longus* (FHL),^18^ *plantaris*,^21^, ^22^ *soleus*, *tibialis anterior* (TA),^92^ and *gasterocnemius* ^22^, ^92^ muscles. Following exposure to an episode of overload‐induced hypertrophy, skeletal muscle was subsequently exposed to disuse atrophy as a model of detraining^18^, ^21^, ^22^, ^92^ or denervation.^16^ Given that young mice (<4 months old) have been shown to display a different response to overload‐induced hypertrophy relative to mature mice (>4 months old),^93^ we performed a subgroup analysis to determine the effects of age on an episode of overload‐induced hypertrophy followed by disuse atrophy on the aforementioned outcome variables. The details of the included studies are shown in *Table* *3*.

##### Myofibre size following training and detraining

Five studies assessed CSA response to increased activity.^16^, ^18^, ^21^, ^22^, ^92^ An episode of overload‐induced hypertrophy significantly increased fibre CSA (SMD = 1.25, 95% CI 0.83–1.67, *p* = 0.00001; *Figure* *S5* *A*). Compared with control, there was no significant difference in fibre CSA after a detraining period (SMD = −0.60, 95% CI −1.71 to 0.51, *P* = 0.29), demonstrating that fibre CSA after a detraining period returns to baseline levels (*Figure* *S5* *B*). Subgroup analysis showed a significant difference between young and mature animals after training and detraining (*P* = 0.04 and *P* = 0.03, respectively), indicating that fibre CSA in young animals increases by a higher extent following training and decreases by a larger extent following detraining.

##### Myonuclear content following training and detraining

Three studies assessed myonuclear content in muscle cross section.^21^, ^22^, ^92^ In response to a hypertrophic stimulus, there was a significant increase in myonuclear content (MD = 0.17, 95% CI 0.09–0.25, *P* = 0.0001; *Figure* *S5* *C*). Myonuclear content remained significantly elevated after a period of detraining compare with control animals (MD = 0.11, 95% CI 0.02–0.20, *P* = 0.01; *Figure* *S5* *D*). The number of studies was too small to permit subgroup analysis.

##### Myonuclear content in single muscle fibre following training and detraining

Four studies assessed myonuclear content in single muscle fibre.^16^, ^18^, ^21^, ^22^ An episode of overload‐induced hypertrophy significantly increased myonuclear content (SMD = 2.26, 95% CI 1.28–3.23, *P* = 0.00001; *Figure* *S5* *E*). Myonuclear content following a period of detraining remained significantly elevated compared with control animals (SMD = 1.46, 95% CI 0.60–2.32, *P* = 0.0008; *Figure* *S5* *F*). Subgroup analysis of maturational age showed no statistically significant difference after overload‐induced hypertrophy or detraining periods (*P* = 0.60 and *P* = 0.43, respectively).

#### Skeletal muscle responses to atrophy

Eighty studies assessed skeletal muscle atrophy in response to different duration of denervation,^1^, ^17^, ^25^ ^,S1–S24^ hindlimb suspension,^15^, ^17^, ^23^, ^24^, ^66^ ^,S25–S57^ immobilization,^28^ ^,S58–S66^ space flight,^27^, ^67^ ^,S67–S69^ tetrodotoxin blockage,^17^ and mechanical ventilation.^S70^ We performed subgroup analyses to determine the potential impact that differences in the muscle under investigation, the duration of the intervention, and the model of atrophy used had on the atrophic response.

##### Myofibre size following atrophy

Analysis of 53 studies found lower CSA in response to skeletal muscle atrophy with a mean reduction of ~−36.9% (SMD = −1.96, 95% CI −2.21 to −1.71, *P* = 0.00001; *Figure* *S6* *A*). Subgroup analysis for different muscles showed fibre CSA was significantly decreased in *plantaris*, *soleus*, *gastrocnemius*, *pectoralis major*, *EDL*, and *TA* (*Figure* *S6* *B*). Subgroup analysis that stratified studies based on duration of the intervention period (≤5 days, 7–14 days, 20–30 days, and ≥42 days) found that myofibre CSA was decreased for all periods (*Figure* *S6* *C*). Subgroup analysis that stratified studies based on the model of atrophy showed that except for mechanical ventilation, all models induced a significant decrease in fibre CSA (*Figure* *S6* *D*). Subgroup analysis that stratified studies based on different methods of atrophy showed that myonuclear content was decreased in studies that performed hindlimb suspension, denervation, and immobilization (*Figure* *S6* *E*). Additionally, subgroup analysis based on %CSA reduction (<20, 20–29, 30–39, 40–49, and >50%) showed that when %CSA reduction reach ≥30%, myonuclear content decreased significantly (*Figure* *S6* *F*). A final subgroup analysis that stratified studies based on different methods of atrophy showed that SC content was decreased only in studies that performed hindlimb suspension (~−24.4%) and immobilization (~−30.1%). More interestingly, SC abundance was increased (~+113.3%) in response to denervation (*Figure* *S6* *G*).

##### Myonuclear content following atrophy

Analysis of 40 studies found lower myonuclear content in muscle cross section with a mean reduction of ~−20.6% (SMD = −1.03, 95% CI −1.30 to −0.76, *P* = 0.00001; *Figure* *S6* *H*). Subgroup analysis that stratified studies based on different muscles showed that myonuclear content was decreased only in *gastrocnemius*, *EDL*, and *soleus* (*Figure* *S6* *I*). Subgroup analysis that stratified studies based on different intervention periods (≤5, 7–14, 20–30, and ≥42 days) showed that myonuclear content was decreased in all periods (*Figure* *S6* *J*).

##### Myonuclear content in single muscle fibre following atrophy

Analysis of 22 studies found lower myonuclear content in single muscle fibres with a mean reduction of approximately −10.1% (SMD = −0.52, 95% CI −0.81 to −0.23, *P* = 0.0005; *Figure* *S6* *K*). Subgroup analyses that stratified studies based on differences in muscle under investigation, duration of the intervention, and model of atrophy used found myonuclear content was only decreased in the *soleus* (*Figure* *S6* *L*). Subgroup analysis that stratified studies based on different intervention periods (≤5 days, 7–14 days, 20–30 days, and ≥42 days) showed that myonuclear content was decreased in studies that lasted between 7–14 and more than 42 days (*Figure* *S6* *M*). Subgroup analysis that stratified studies based on different models of atrophy showed that myonuclear content was decreased in studies that performed hindlimb suspension and denervation (*Figure* *S6* *N*). Considering the different muscle type responses to atrophy, the discrepancy between the results for myonuclear content in whole muscle cross section and single muscle fibres may be due to the lower and selected fibre measurements in the studies that used single muscle fibre, as no more than 100 fibres were evaluated in any study.

##### Satellite cell number following atrophy

Analysis of 41 studies found no change in SC content in cross‐section (SMD = −0.13, 95% CI −0.50 to −0.24, *P* = 0.48) (*Figure* *S6* *O*). Subgroup analysis that stratified studies based on different muscles showed that SC content was decreased in *soleus*, whereas in TA it increased, and in EDL tend to increase (*Figure* *S6* *P*). Subgroup analysis that stratified studies based on different intervention periods (≤5, 7–14, 18–30, and ≥42 days) showed a trend for lower SC content only in studies that lasted between 7 and 14 days (*Figure* *S6* *Q*).

### Sensitivity analysis and publication bias

In regard to sensitivity analysis, the overall pooled estimates of the respective outcomes obtained in each analysis closely resembled the preliminary associations. Further, funnel plots were checked for the included studies in the meta‐analysis, which suggested that in almost all analyses in human studies, there is no noticeable bias (*Figure* *S7A* *–* *S7D*). Additionally, *Begg's* correlation rank and *Egger's* regression did not show significant publication bias in almost all analyses in human studies (*Table* *4*). In contrast, we found noticeable publication bias in most analyses of animal studies with significant *Begg's* correlation rank and *Egger's* regression results (*Figure S7E* and *S7F*; *Table* *4*).

**Table 4:** Meta‐analysis of all studies

Subgroup analysis	Classification	Heterogeneity		Model	Meta‐analysis			
		*P*	*I* ^2^ (%)		SMD (95% CI)	*P*	Beggs' *P* value	Eggers' *P* value
*Human studies: skeletal muscle responses to hypertrophy*								
Outcome: CSA in whole cross section								
Mixed fibre	After training	0.6	0%	Fixed	650.32 (355.30, 945.34)	0.0001	1.0000	0.324
	After detraining	0.01	72%	Random	83.46 (−649.41, 816.32)	0.82	0.7341	0.144
Type I fibres	After training	0.72	0%	Fixed	470.83 (168.29, 773.37)	0.002	1.0000	0.618
	After detraining	0.07	62%	Random	104.39 (−604.46, 813.23)	0.77	0.2963	0.309
Type II fibres	After training	0.32	13%	Fixed	723.93 (358.02, 1089.84)	0.0001	1.0000	0.363
	After detraining	0.04	70%	Random	190.74 (−882.92, 1264.40)	0.73	0.2963	0.200
Outcome: Myonuclear content								
Mixed fibres	After training	0.58	0%	Fixed	0.12 (0.00, 0.23)	0.04	0.7341	0.491
	After detraining	0.52	0%	Fixed	−0.14 (−0.26, −0.02)	0.02	1.0000	0.993
Type I fibres	After training	0.70	0%	Fixed	0.04 (−0.08, 0.15)	0.55	1.0000	0.764
	After detraining	0.96	0%	Fixed	‐0.14 (−0.28, −0.00)	0.05	1.0000	−0.71
Type II fibres	After training	0.4	0%	Fixed	0.23 (0.07, 0.40)	0.006	1.0000	0.349
	After detraining	0.61	0%	Fixed	−0.23 (−0.37, −0.10)	0.0009	1.0000	0.733
Outcome: Myonuclear domain								
Mixed fibres	After training	0.2	36%	Fixed	110.91 (24.93, 196.89)	0.01	0.7341	0.437
	After detraining	0.55	0%	Fixed	43.16 (−42.14, 128.47)	0.32	0.3082	0.349
Type I fibres	After training	0.34	0%	Fixed	5.67 (−133.51, 144.85)	0.94	IO	IO
	After detraining	0.42	0%	Fixed	−9.26 (−166.29, 147.77)	0.91	IO	IO
Type II fibres	After training	0.8	0%	Fixed	73.87 (−62.35, 210.09)	0.29	IO	IO
	After detraining	0.48	0%	Fixed	55.98 (−138.18, 250.14)	0.57	IO	IO
Outcome: Satellite cells								
Mixed fibres	After training	0.52	0%	Fixed	0.75 (0.33, 1.18)	0.0005	1.0000	0.814
	After detraining	0.84	0%	Fixed	0.16 (−0.32, 0.64)	0.52	1.0000	0.808
Type I fibres	After training	0.77	0%	Fixed	0.36 (−0.14, 0.85)	0.16	IO	IO
	After detraining	0.58	0%	Fixed	−0.01 (−0.66, 0.65)	0.99	IO	IO
Type II fibres	After training	0.98	0%	Fixed	0.81 (0.30, 1.32)	0.002	IO	IO
	After detraining	0.74	0%	Fixed	0.09 (−0.57, 0.74)	0.79	IO	IO
*Human studies: skeletal muscle responses to atrophy*								
Outcome: CSA								
Mixed fibre	NA	0.002	61%	Random	−497.24 (−734.13, −260.35)	0.0001	0.0022	0.005
Type I fibres	NA	0.0001	62%	Random	−735.16 (−1062.57, −407.75)	0.0001	0.0369	0.089
Type II fibres	NA	0.00001	71%	Random	−919.18 (−1292.14, −546.22)	0.00001	0.0241	0.102
Outcome: Myonuclear content								
Mixed fibre	NA	0.12	32%	Fixed	−0.11 (−0.19, −0.03)	0.005	0.0160	0.052
Type I fibres	NA	0.71	0%	Fixed	−0.09 (−0.17, −0.00)	0.04	0.2129	0.141
Type II fibres	NA	0.06	44%	Fixed	−0.13 (−0.22, −0.05)	0.003	0.1367	0.164
Outcome: Myonuclear domain								
Mixed fibre	NA	0.0001	72%	Fixed	−1.92 (−2.72, −1.12)	0.00001	0.7555	0.520
Type I fibres	NA	0.63	0%	Fixed	−0.65 (−0.97, −0.32)	0.0001	0.5362	0.798
Type II fibres	NA	0.68	0%	Fixed	−0.72 (−1.03, −0.40)	0.0001	0.3865	0.712
*Human studies: skeletal muscle responses to atrophy*								
Outcome: Satellite cells								
Mixed fibre	NA	0.0001	61%	Random	−0.49 (−0.77, −0.22)	0.0005	0.0232	0.000
Type I fibres	NA	0.00001	71%	Random	−0.20 (−0.59, 0.20)	0.33	0.5289	0.081
Type II fibres	NA	0.0001	63%	Random	−0.37 (−0.71, −0.02)	0.04	0.4415	0.022
*Human studies: Skeletal muscle responses in ageing compared with young adults*								
Outcome: CSA								
Mixed fibres	NA	0.01	63%	Random	0.91 (0.25, 1.56)	0.007	0.0163	0.107
Type I fibres	NA	0.02	42%	Random	−131.70 (−353.91, 90.51)	0.25	0.8215	0.283
Type II fibres	NA	0.0001	61%	Random	1313.31 (995.45, 1631.16)	0.00001	0.5728	0.189
Outcome: Myonuclear domain								
Mixed fibres	NA	0.0001	83%	Random	236.01 (−11.78, 483.79)	0.06	0.8065	0.955
Type I fibres	NA	0.0001	79%	Random	−26.75 (−207.05, 153.56)	0.77	0.7105	0.646
Type II fibres	NA	0.0002	75%	Random	296.19 (109.08, 483.29)	0.002	0.2655	0.502
Outcome: Satellite cells								
Mixed fibres	NA	0.00001	67%	Random	0.78 (0.37, 1.19)	0.0002	0.0179	0.006
Type I fibres	NA	0.13	30%	Fixed	0.09 (−0.11, 0.28)	0.38	0.9212	0.933
Type II fibres	NA	0.0004	64%	Random	1.23 (0.86, 1.60)	0.00001	0.0478	0.560
Outcome: Myonuclear content								
Mixed fibres	NA	0.00001	82%	Random	−0.03 (−0.24, 0.19)	0.8	0.2464	0.092
Type I fibres	NA	0.003	67%	Random	−0.07 (−0.53, 0.39)	0.76	0.7105	0.708
Type II fibres	NA	0.01	61%	Random	0.58 (0.15, 1.02)	0.008	0.7105	0.353
*Animal studies: skeletal muscle responses to hypertrophy*								
Outcome: CSA in whole cross‐section								
Mean CSA	Control vs training	0.15	37%	Fixed	1.25 (0.83, 1.67)	0.00001	0.0163	0.091
	Control vs detraining	0.00001	85%	Random	−0.60 (−1.71, 0.51)	0.29	0.229	0.015
Outcome: Myonuclear content in whole cross section								
Myonuclear content	Control vs training	0.06	60%	Random	0.17 (0.09, 0.25)	0.0001	0.0894	0.149
	Control vs detraining	0.06	59%	Random	0.11 (0.02, 0.20)	0.01	0.0894	0.251
Outcome: Myonuclear content in single muscle fibre								
Myonuclear content	Control vs training	0.01	66%	Random	2.26 (1.28, 3.23)	0.00001	0.0085	0.062
	Control vs detraining	0.007	68%	Random	1.46 (0.60, 2.32)	0.0008	0.0085	0.033
*Animal studies: skeletal muscle responses to atrophy*							
Outcome: CSA in whole cross section							
Mean CSA	NA	0.00001	0.63%	Random	−1.96 (−2.21, −1.71)	0.00001	0.0000	0.000
Outcome: Myonuclear content in whole cross section							
Myonuclear content	NA	0.00001	65%	Random	−1.03 (−1.30, −0.76)	0.00001	0.0000	0.000
Outcome: Satellite cells in whole cross section							
Satellite cells	NA	0.00001	81%	Random	−0.13 (−0.50, 0.24)	0.48	0.5724	0.266
Outcome: Myonuclear content in single muscle fibre								
Myonuclear content	NA	0.00001	62%	Random	−0.52 [−0.81, −0.23]	0.0005	0.0000	0.000

CSA, cross‐sectional area; IO, insufficient observation; NA, not applicable; SMD, standard mean difference.

## Discussion

The objective of the current systematic review and meta‐analysis was to assess the myonuclear and SC content of either human or rodent skeletal muscle that had undergone hypertrophy, atrophy, or detraining. We found that both myonuclear and SC content in human skeletal muscle are lower with atrophy, ageing, and following a period of detraining; however, the change in myonuclear and SC content with detraining represents a return to pre‐training levels. Subgroup analyses that stratified studies based on the age of the subjects showed that following detraining, Type I CSA in young adults decreases to a higher extent than in old adults. Additionally, following atrophy in human studies, we found that both myonuclear and SC content in mixed, Type I, and Type II fibres only decreased in young adults. In rodent studies, myonuclear content after an episode of overload‐induced hypertrophy remains elevated during the subsequent detraining period. With atrophy in rodents, myonuclear content is sensitive to the muscle type and the model of atrophy. More interestingly, we found that in animals, an atrophy of myofibre CSA of ≥30% was associated with a significant decrease in myonuclei.

Skeletal muscle fibres have a memory of prior chronic contractile activity, termed ‘muscle memory’. Evidence suggests myonuclei acquired during an initial period of hypertrophy are associated with enhanced muscle growth upon resumption of training following a period of detraining.^1^, ^15^, ^16^, ^17^ An obvious, but debated, critical aspect of this proposed mechanism of muscle memory is the ‘new’ myonuclei must be retained throughout the period of detraining.^16^, ^17^ The present meta‐analysis found that exercise‐induced myonuclei were not retained during detraining in humans but were in rodents. The rodent finding should be viewed with some caution as only five studies were included in the analysis with one study using denervation as a model of detraining following synergist ablation‐induced hypertrophy.^16^ The concern with denervation as a model of detraining stems from our meta‐analysis showing that denervation in rodent skeletal muscle causes a significant increase in SC content. Thus, it is not clear if the elevated myonuclear content reported by Bruusgaard *et al*.^16^ after denervation‐induced atrophy was driven by enhanced SC fusion, which would mask any loss of myonuclei. Other concerns that need to be taken into consideration are the magnitude of the hypertrophic response and the age of animals. The 25–60% increase in skeletal muscle CSA in response to synergist ablation^S71–S75^ is much higher than 6–10% increase in quadriceps CSA in response to resistance training in humans.^S75–S79^ Furthermore, three of the five rodent studies used animals under 4 months old.^16^, ^18^, ^92^ Considering the different SC requirements for hypertrophic growth in fully mature mice compared with juvenile mice,^93^ the elevated myonuclear content during detraining might reflect a low level of SC fusion known to occur in juvenile mice.^S80^ Additional animal studies are needed to more definitively answer the question of whether or not myonuclei acquired during hypertrophy are permanent during periods of detraining. Moreover, evaluating the same muscle in human studies (*vastus lateralis*) and different muscles in rodent studies (including *EDL*, *FHL*, *gastrocnemius*, *soleus*, and *plantaris*) resulted in very high heterogeneity in myonuclear content analysis in rodents (*I* ^2^ = 60–80%) but absolute homogeneity in humans (*I* ^2^ = 0%).

The meta‐analysis for atrophy in humans found that young adults respond differently to atrophy stimuli than old adults; myonuclear and SC content in mixed, Type I, and Type II fibres only decreased in young adults in response to atrophy. The influence of age on skeletal muscle plasticity is also observed in rodent studies, which found that juvenile mice (8 weeks of age) display a different response to overload‐induced hypertrophy relative to mature (16 weeks of age) mice; SC depletion in juvenile mice prevents hypertrophic growth, whereas skeletal muscle fibres in mature mice grow following SC depletion despite the lack of myonuclear accretion.^93^ The results of our meta‐analysis indicate that in young adults, skeletal muscle atrophy is accompanied by a decrease in myonuclear and SC content, although changes in the myonuclear domain control skeletal muscle size in old adults. The rodent meta‐analysis for atrophy found that myonuclear content is sensitive to muscle type as the abundance of myonuclei may not change in some muscles. In this regard, following a detraining period, some muscles (like *gastrocnemius* and *plantaris*) lose their myonuclei, whereas other muscles (such as the *soleus*) with different activation patterns are resistant to the loss of myonuclei.^22^ Hence, more pre‐clinical research using the various interventions in the same muscle is warranted. Interestingly, the magnitude of myonuclear elevation in rodent studies was about 2.6 times higher than in human studies (23.2% in animals vs. 9% in humans) and is reduced by 6.6% in animals after a detraining period. This finding indicates that even in rodents, elevated myonuclear content is not retained indefinitely but may decrease to a lower extent compared with humans. Additionally, the greater magnitude of hypertrophy in rodents was associated with a higher myonuclear content of approximately 18%, whereas in humans, it was ~11%. This finding provides further support for the notion that changes in myonuclear content influence the magnitude of muscle hypertrophy. Meta‐analysis of atrophy in humans found that myonuclear content was lower with only 9% atrophy. The rodent studies that directly assessed muscle memory showed no change in myonuclear number with atrophy of 10%; however, when atrophy was ≥30% in rodents, myonuclear content was lower. These findings reveal that, in rodents, myonuclear content is stable, except under the most extreme atrophic conditions.^22^

Needing more evidence in both humans and rodents, we decided to assess myonuclear content and SC numbers after exposure to atrophy. The results of our meta‐analysis showed that myonuclear content and SCs of atrophied human Type II fibres decrease following atrophy. This analysis also found that myonuclear content in rodents decreases in response to hindlimb suspension, denervation, and immobilization with SC content lower in response to hindlimb suspension and immobilization. These findings implicate that myonuclear and SC content in both humans and rodents are not maintained indefinitely and may be reduced with skeletal muscle atrophy. Interestingly, we found lower myonuclear content was associated with higher SC content in response to denervation. These results can be explained by a higher rate of atrophy and a lower rate of myonuclear reduction in response to denervation (44 vs. 16%, respectively) compare with hindlimb suspension (35 vs. 25%, respectively), and immobilization (28 vs. 19%, respectively). Finally, needing more evidence regarding the possibility of long‐term myonuclear permanence in humans, we assessed myonuclear content, MND, and SC numbers in studies that compared young and elderly adults. Interestingly, we found that human ageing is accompanied by reduction in myonuclear content, MND, and SC abundance in atrophied Type II myofibres. These results clearly demonstrate that myonuclei are not retained indefinitely throughout the human lifespan.

To better understand how skeletal muscle possesses a memory of prior chronic contractile activity, recent studies have focused on the potential role of epigenetics. Skeletal muscle may possess a long‐term DNA hypomethylation ‘memory’ of prior exercise training that could have consequences for future myofibres adaptability during retraining.^94^, ^95^, ^96^ Future studies should evaluate the role of epigenetic ‘memory’ association with a first training period to extend our understanding of the molecular bases of ‘muscle memory’.

### Limitations

There are several limitations of the systematic review and meta‐analysis. First, despite the intense interest in the concept of ‘muscle memory’, the evidence to support the concept remains anecdotal as illustrated by the paucity of human and animal studies (i.e. only five studies in animals and four studies in humans). Second, different muscles were analysed across the animal studies, which confounded the results. Third, the different rates of muscle hypertrophy and myonuclear accretion between humans and animals make it quite challenging to translate animal results to *in vivo* human setting. Fourth, the small number of human studies made it challenging to determine the relationship between myonuclear content and the degree of atrophy as observed in rodents. Fifth, the analysis of SC content during atrophy in human studies associated with different diseases or models of atrophy was unable to identify a loss of SC content is related to a particular disease state or model of atrophy. Sixth, the current meta‐analysis is based on the assumption that all studies accurately measured myonuclear content. To accurately quantify myonuclear abundance by muscle cross section (which represents the vast majority of the studies analysed), it is critical to clearly identify the myofibre cell border; yet this approach can be hampered by the fact that a three‐dimensional structure, that is, the myofibre is being assessed in two dimensions. This can lead to the mis‐identification of a satellite cell nucleus being inside the myofibre or, alternatively, a *bona fide* myonucleus not being counted as it appears outside the dystrophin border. While this scenario is possible, it is assumed to have a minor impact, if at all, on the quantification of myonuclear content. We generated a new transgenic mouse model that allows for the definitive identification of myonuclei via nuclear GFP‐labelling, which should help to further minimize this inherent limitation of quantifying myonuclear content by muscle cross section.^97^ Finally, the meta‐analysis of animal studies should be interpreted with caution as publication bias may be present.

### Conclusion

The findings of this study extend and add new information to the field's knowledge regarding the concept of ‘muscle memory’ based on the idea that, once myonuclei are acquired, they are permanent. In humans, myonuclear content is not stable as it was found to change in response to a bout of detraining or atrophy. This finding suggests that other mechanisms are operative in mediating muscle memory. In rodents, the stability of myonuclei is less clear because of the limited number of studies and differences in experimental design across studies.

## Conflict of interest

The authors declare that they have no conflicts of interest relevant to the content of this review.

## Funding

This work has been supported by the Lorestan University.

## Supporting information


**Figure S1A:** Results of the risk of bias and methodological quality indicators at an individual level (A) and all included studies in the systematic review (B) that evaluated skeletal muscle responses to hypertrophy in humans. The items were scored on the Physiotherapy Evidence Database (PEDro) scale.
**Figure S1B:** Results of the risk of bias and methodological quality indicators at an individual level (A) and all included studies in the systematic review (B) that evaluated skeletal muscle responses to atrophy in humans. The items were scored on the Physiotherapy Evidence Database (PEDro) scale.
**Figure S1C:** Results of the risk of bias and methodological quality indicators at an individual level (A) and all included studies in the systematic review (B) that compared skeletal muscles of old versus young peoples. The items were scored on the Physiotherapy Evidence Database (PEDro) scale.
**Figure S1D:** Results of the risk of bias and methodological quality indicators at an individual level (A) and all included studies in the systematic review (B) that evaluated skeletal muscle responses to hypertrophy in animals. The items in the Systematic Review Centre for Laboratory Animal Experimentation (SYRCLE) risk of bias assessment were scored with “yes” indicating low risk of bias, “no” indicating high risk of bias, or “unclear” indicating that the item was not reported, resulting in an unknown risk of bias.
**Figure S1E:** Results of the risk of bias and methodological quality indicators at an individual level (A) and all included studies in the systematic review (B) that evaluated skeletal muscle responses to atrophy in anaimals. The items in the Systematic Review Centre for Laboratory Animal Experimentation (SYRCLE) risk of bias assessment were scored with “yes” indicating low risk of bias, “no” indicating high risk of bias, or “unclear” indicating that the item was not reported, resulting in an unknown risk of bias.

**Figure S2.** Meta‐analysis results for skeletal muscle responses to hypertrophy in human studies.

**Figure S3.** Meta‐analysis results for skeletal muscle responses to atrophy in human studies.

**Figure S4.** Meta‐analysis results for skeletal muscle responses in aging compared with young adults in human studies.

**Figure S5.** Meta‐analysis results for skeletal muscle responses to hypertrophy in animal studies.

**Figure S6.** Meta‐analysis results for skeletal muscle responses to atrophy in animal studies.

**Figure S7A.** Funnel plots for publication bias on skeletal muscle responses to hypertrophy in human studies after training.
**Figure S7B.** Funnel plots for publication bias on skeletal muscle responses to hypertrophy in human studies after detraining.
**Figure S7C.** Funnel plots for publication bias on skeletal muscle responses to atrophy in human studies.
**Figure S7D.** Funnel plots for publication bias on skeletal muscle responses in aging compared with young adults.
**Figure S7E.** Funnel plots for publication bias on skeletal muscle responses to hypertrophy in animal studies.
**Figure S7F.** Funnel plots for publication bias on skeletal muscle responses to atrophy in animal studies.

**Data S1.** Supporting Information
