# Supplementary material for: Myonuclear permanence in skeletal muscle memory: a systematic review and meta‐analysis of human and animal studies
Source: J Cachexia Sarcopenia Muscle. 2022 Aug 12;13(5):2276–97. doi: 10.1002/jcsm.13043 (PMC9530508; doi:10.1002/jcsm.13043)
Supplement: Supplementary file 1 — Figure S1A: Results of the risk of bias and methodological quality indicators at an individual level (A) and all included studies in the systematic review (B) that evaluated skeletal muscle responses to hypertrophy in humans. The items were scored on the Physiotherapy Evidence Database (PEDro) scale. Figure S1B: Results of the risk of bias and methodological quality indicators at an individual level (A) and all included studies in the systematic review (B) that evaluated skeletal muscle responses to atrophy in humans. The items were scored on the Physiotherapy Evidence Database (PEDro) scale. Figure S1C: Results of the risk of bias and methodological quality indicators at an individual level (A) and all included studies in the systematic review (B) that compared skeletal muscles of old versus young peoples. The items were scored on the Physiotherapy Evidence Database (PEDro) scale. Figure S1D: Results of the risk of bias and methodological quality indicators at an individual level (A) and all included studies in the systematic review (B) that evaluated skeletal muscle responses to hypertrophy in animals. The items in the Systematic Review Centre for Laboratory Animal Experimentation (SYRCLE) risk of bias assessment were scored with “yes” indicating low risk of bias, “no” indicating high risk of bias, or “unclear” indicating that the item was not reported, resulting in an unknown risk of bias. Figure S1E: Results of the risk of bias and methodological quality indicators at an individual level (A) and all included studies in the systematic review (B) that evaluated skeletal muscle responses to atrophy in anaimals. The items in the Systematic Review Centre for Laboratory Animal Experimentation (SYRCLE) risk of bias assessment were scored with “yes” indicating low risk of bias, “no” indicating high risk of bias, or “unclear” indicating that the item was not reported, resulting in an unknown risk of bias. [file JCSM-13-2276-s003.docx]

**Figure S1A:** Results of the risk of bias and methodological quality indicators at an individual level (A) and all included studies in the systematic review (B) that evaluated skeletal muscle responses to hypertrophy in humans. The items were scored on the [Physiotherapy Evidence Database (PEDro) scale](https://journals.plos.org/plosone/article?id=10.1371/journal.pone.0222770).

**A**

**
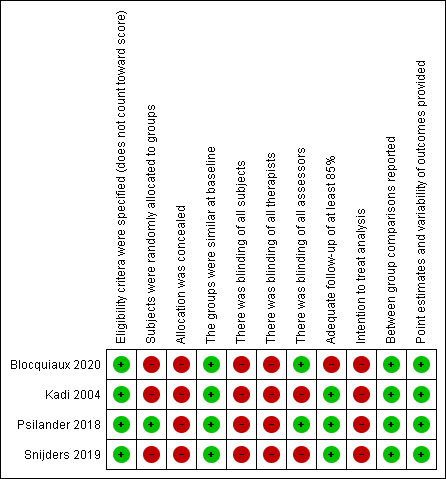
**

**B**

**
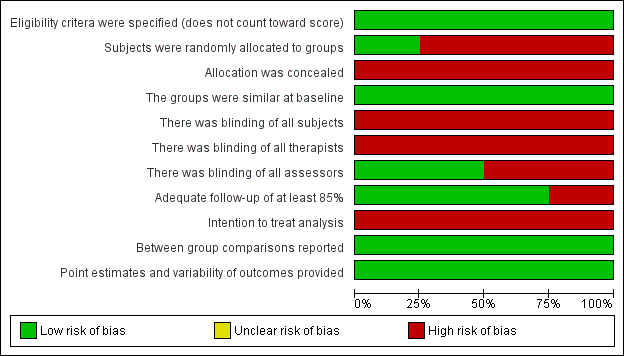
**

**Figure S1B:** Results of the risk of bias and methodological quality indicators at an individual level (A) and all included studies in the systematic review (B) that evaluated skeletal muscle responses to atrophy in humans. The items were scored on the [Physiotherapy Evidence Database (PEDro) scale](https://journals.plos.org/plosone/article?id=10.1371/journal.pone.0222770).


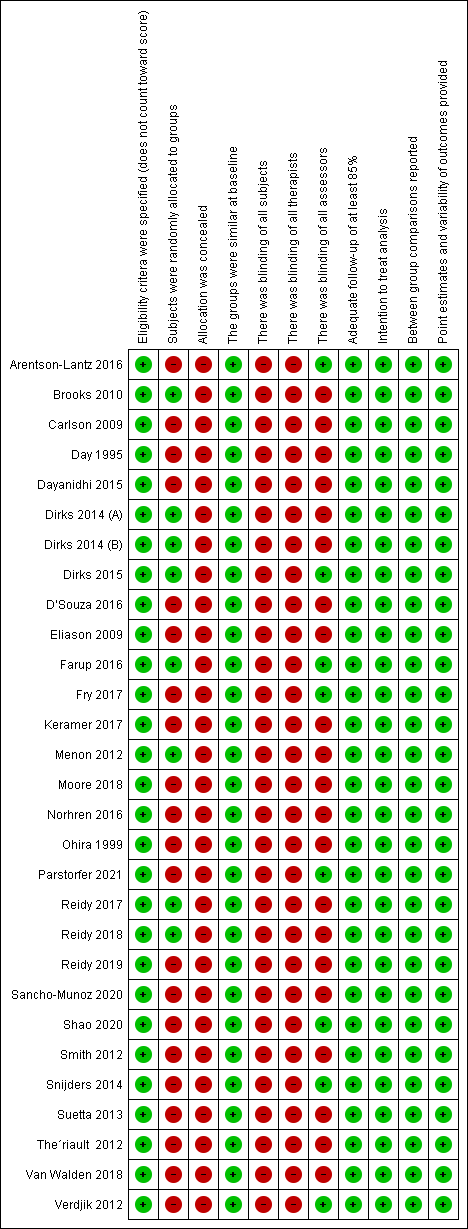


**A**

**B**

**
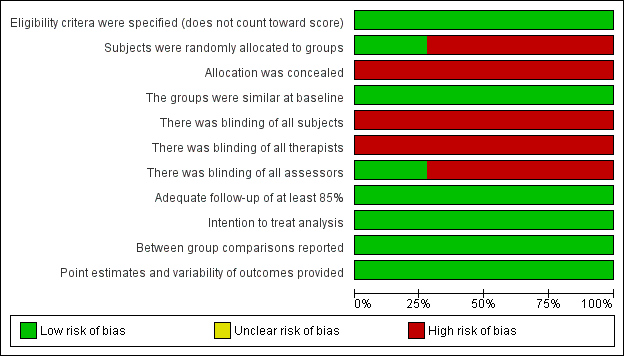
**

**Figure S1C:** Results of the risk of bias and methodological quality indicators at an individual level (A) and all included studies in the systematic review (B) that compared skeletal muscles of old versus young peoples. The items were scored on the [Physiotherapy Evidence Database (PEDro) scale](https://journals.plos.org/plosone/article?id=10.1371/journal.pone.0222770).

**A**

**
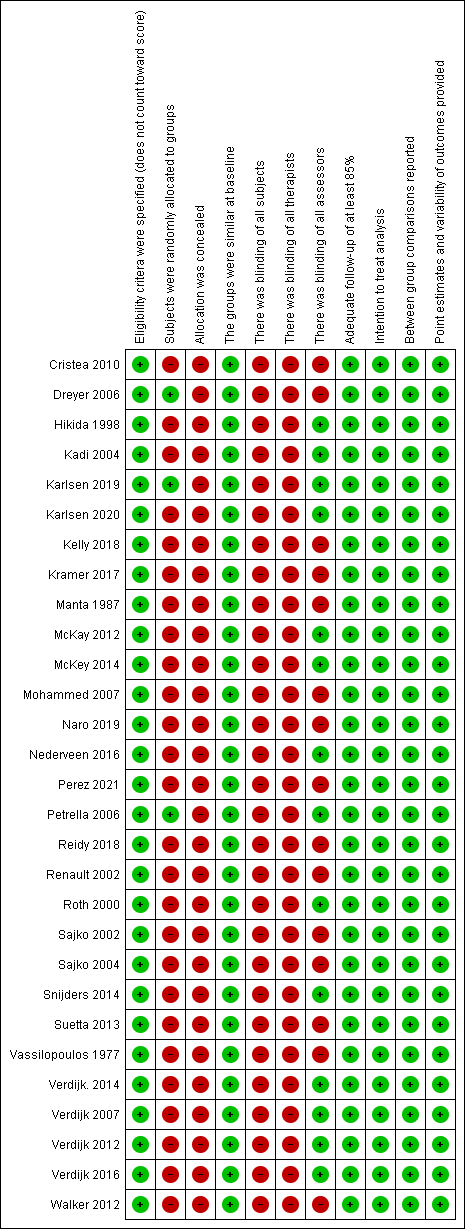
**

**B**

**
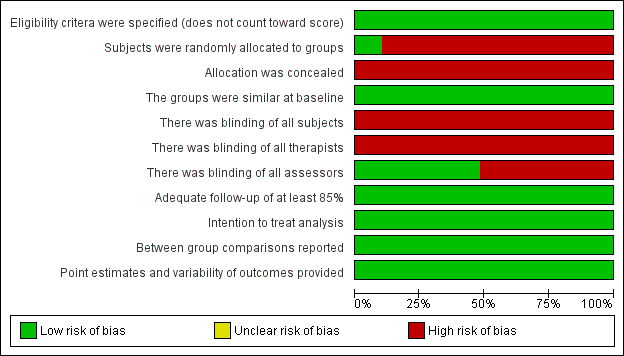
**

**Figure S1D:** Results of the risk of bias and methodological quality indicators at an individual level (A) and all included studies in the systematic review (B) that evaluated skeletal muscle responses to hypertrophy in animals. The items in the Systematic Review Centre for Laboratory Animal Experimentation (SYRCLE) risk of bias assessment were scored with “yes” indicating low risk of bias, “no” indicating high risk of bias, or “unclear” indicating that the item was not reported, resulting in an unknown risk of bias.

**A**

**
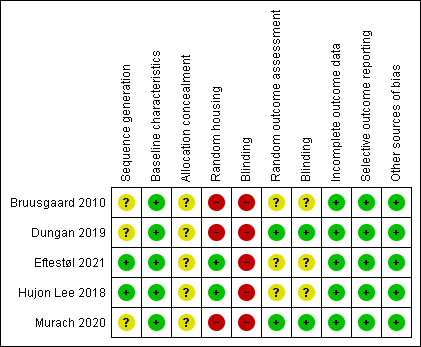
**

**B**

**
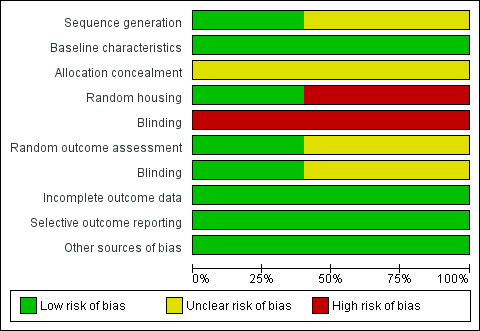
**

**Figure S1E:** Results of the risk of bias and methodological quality indicators at an individual level (A) and all included studies in the systematic review (B) that evaluated skeletal muscle responses to atrophy in anaimals. The items in the Systematic Review Centre for Laboratory Animal Experimentation (SYRCLE) risk of bias assessment were scored with “yes” indicating low risk of bias, “no” indicating high risk of bias, or “unclear” indicating that the item was not reported, resulting in an unknown risk of bias.

**B**


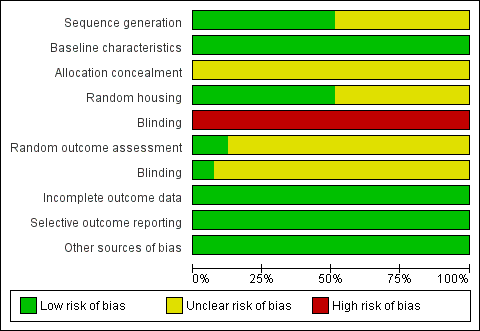


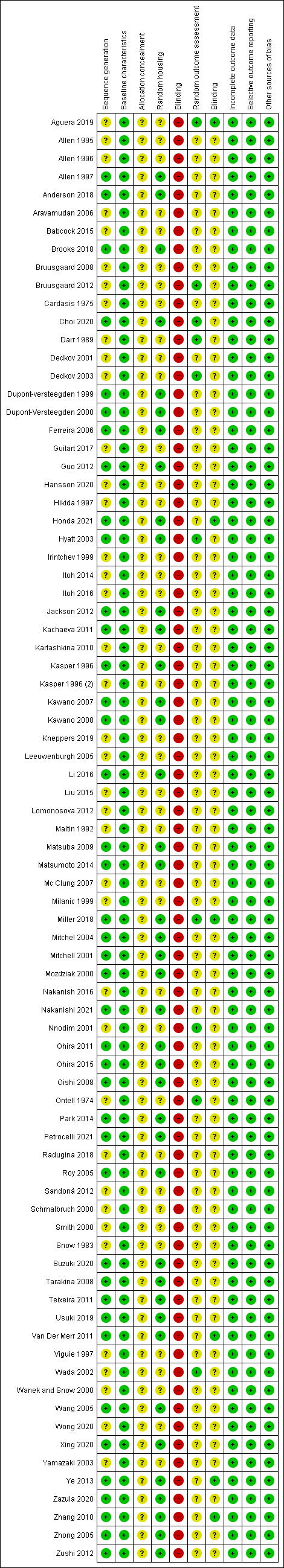


**A**
