# Supplementary material for: Myonuclear permanence in skeletal muscle memory: a systematic review and meta‐analysis of human and animal studies
Source: J Cachexia Sarcopenia Muscle. 2022 Aug 12;13(5):2276–97. doi: 10.1002/jcsm.13043 (PMC9530508; doi:10.1002/jcsm.13043)
Supplement: Supplementary file 2 — Figure S2. Meta‐analysis results for skeletal muscle responses to hypertrophy in human studies. [file JCSM-13-2276-s004.docx]

**Figure 2S. Meta-analysis results for skeletal muscle responses to hypertrophy in human studies.**

**2SA. Skeletal muscle CSA in mixed fibers after training.**


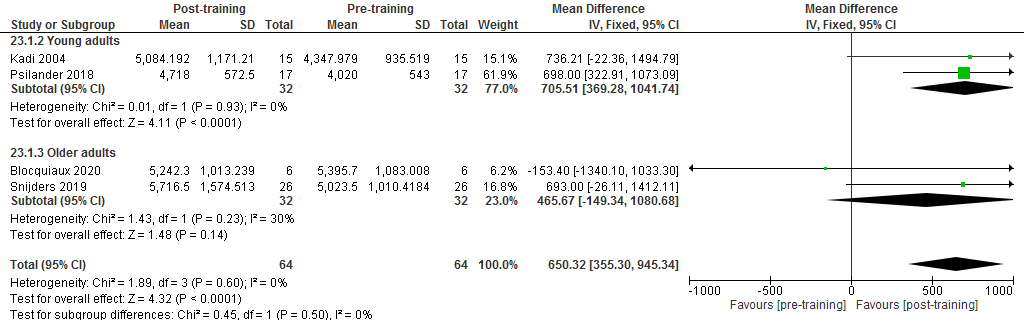


**2SB. Skeletal muscle CSA in type I fibers after training.**


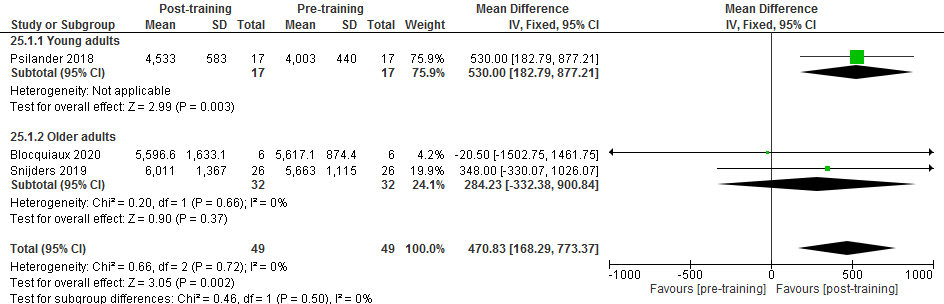


**2SC. Skeletal muscle CSA in type II fibers after training.**


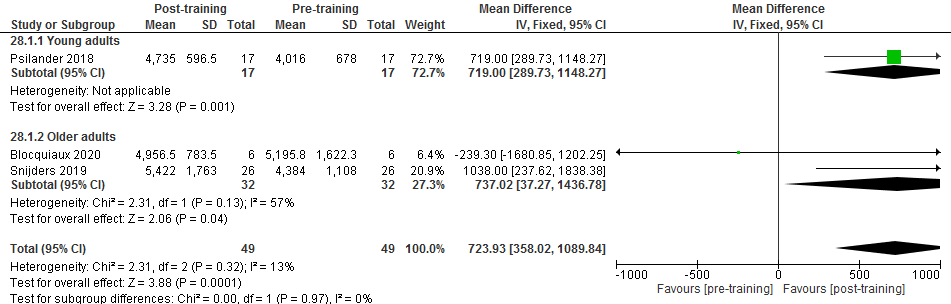


**2SD. Skeletal muscle CSA in mixed fibers after detraining.**


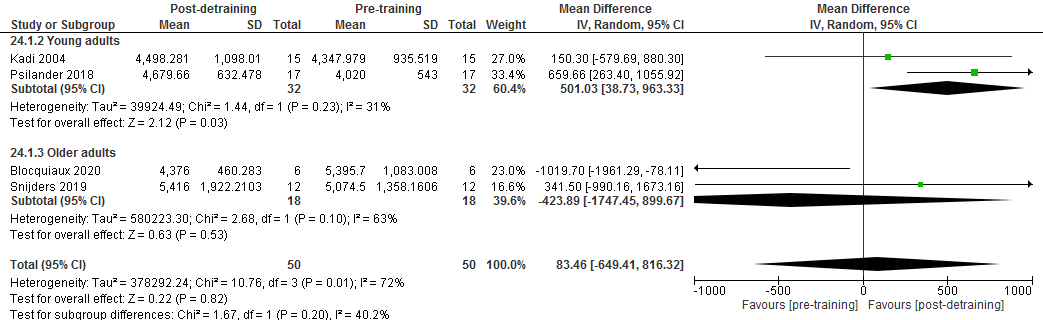


**2SE. Skeletal muscle CSA in type I fibers after detraining.**


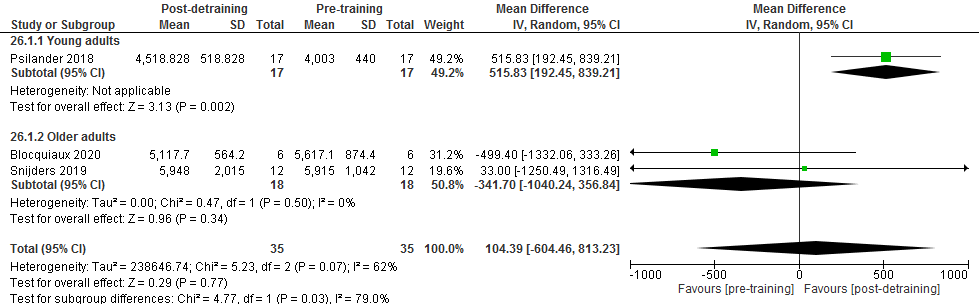


**2SF. Skeletal muscle CSA in type II fibers after detraining.**


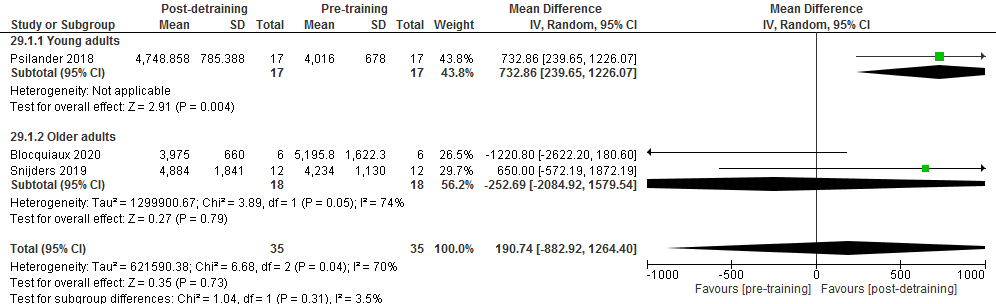


**Note:** Compared with baseline values, mean improvement of CSA after resistance training ranged from ~11.3% in mixed fibers to ~6.3% in type I and ~12.3% in type II fibers. However, compared with baseline values, the mean improvement of CSA after a detraining period ranged from ~2% in mixed fibers to ~1.5% in type I and ~3.4% in type II fibers.

**2SG. Myonuclear content in mixed after training.**


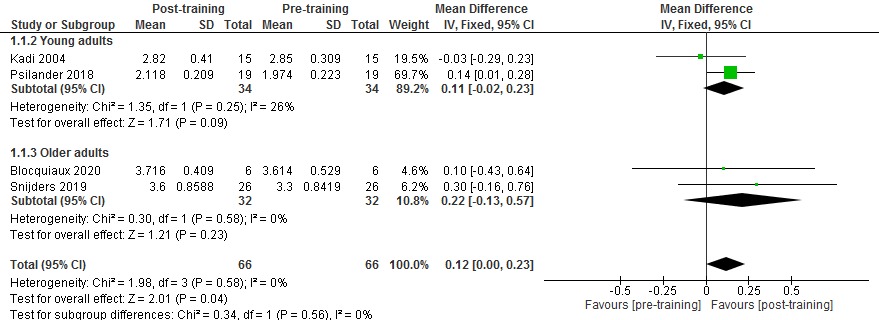


**2SH. Myonuclear content in type I fibers after training.**


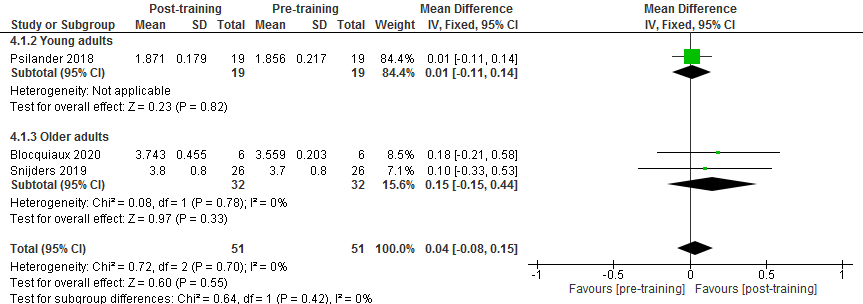


**2SI. Myonuclear content in type II fibers after training.**


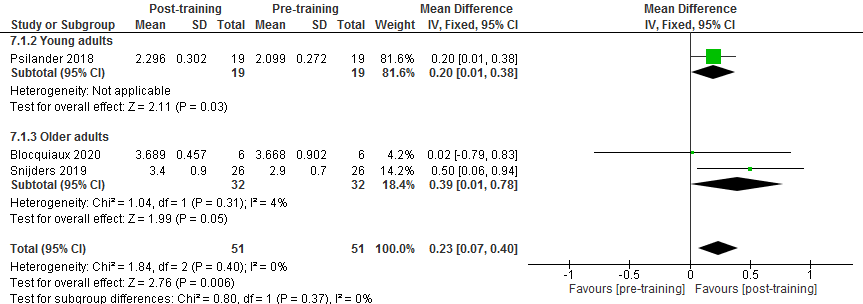


**2SJ. Myonuclear content in mixed fibers after detraining.**


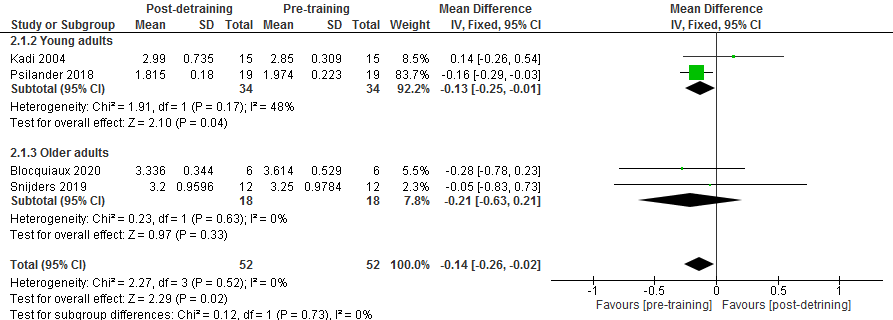


**2SK. Myonuclear content in type I fibers after detraining.**


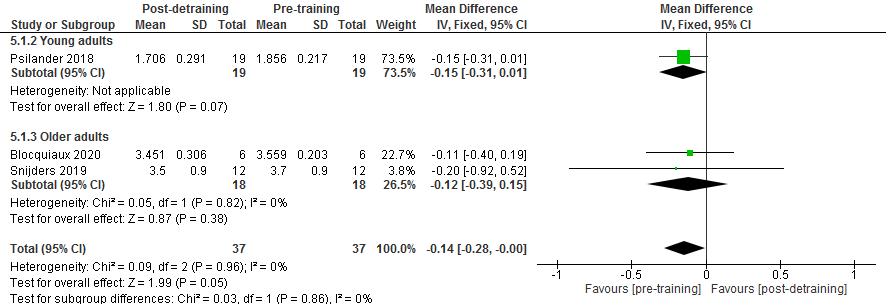


**2SL. Myonuclear content in type II fibers after detraining.**


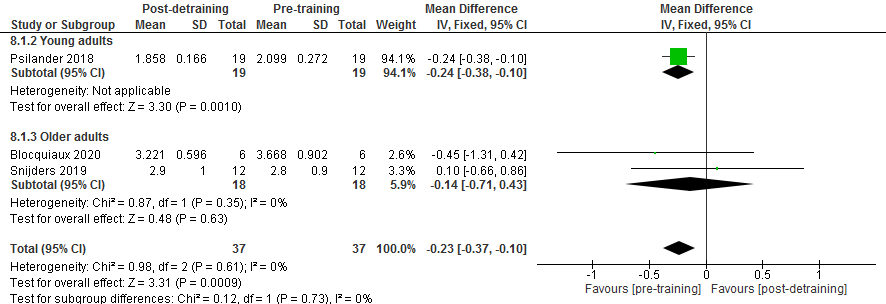


**Note:** Compared with baseline values, mean improvement of myonuclear content after resistance training ranged from ~4.5% in mixed fibers to ~2. 9% in type I and ~9% in type II fibers. However, compared with baseline values, the mean improvement of myonuclear content after a detraining period ranged from ~-3% in mixed fibers to ~-5.5% in type I and ~-6.7% in type II fibers.

**2SM. MND in mixed fibers after training.**


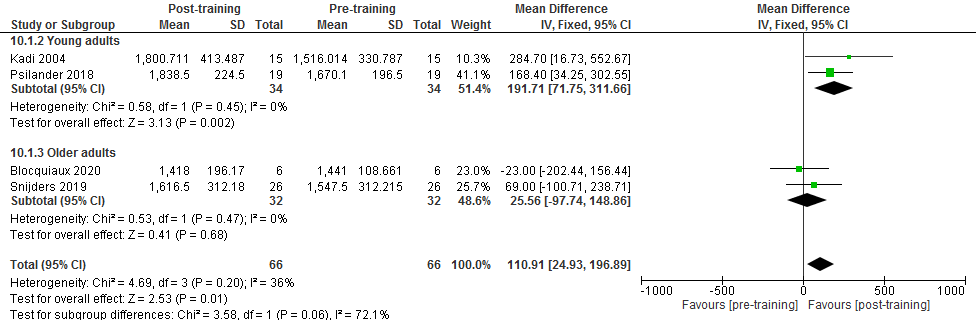


**2SN. MND in type I fibers after training.**


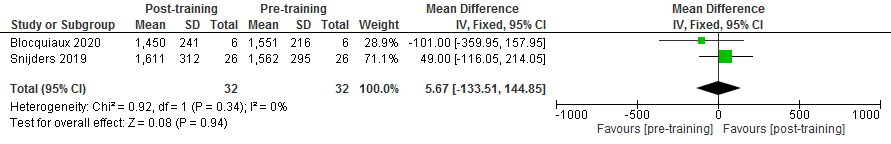


**2SO. MND in type II fibers after training.**


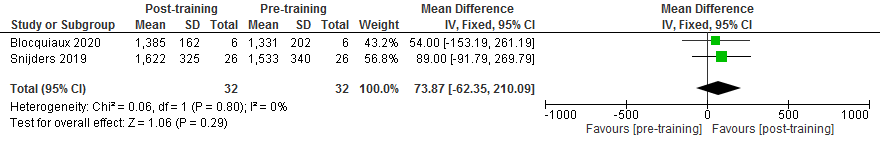


**2SP. MND in mixed fibers after detraining.**


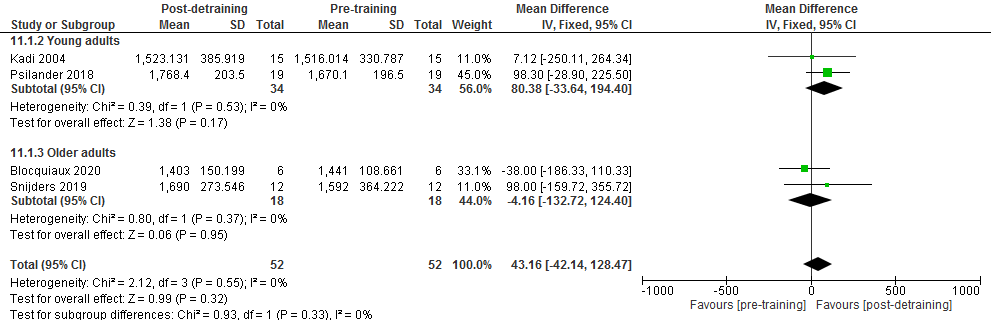


**2SQ. MND in type I fibers after detraining.**


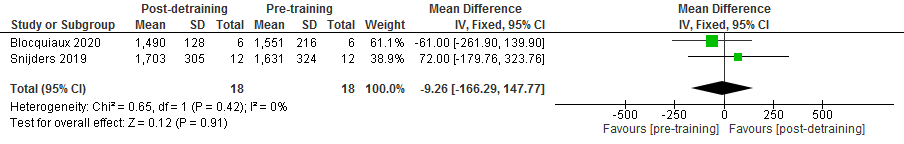


**2SR. MND in type II fibers after detraining.**


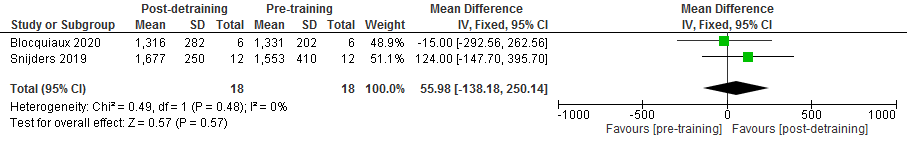


**Note:** Compared with baseline values, mean improvement of MND after resistance training ranged from ~7.9% in mixed fibers to ~-1.6% in type I and ~4.1% in type II fibers. However, compared with baseline values, the mean improvement of MND after a detraining period ranged from ~-2.5% in mixed fibers to ~0.2% in type I and ~4.1% in type II fibers.

**2SS. SC in mixed fibers after training.**


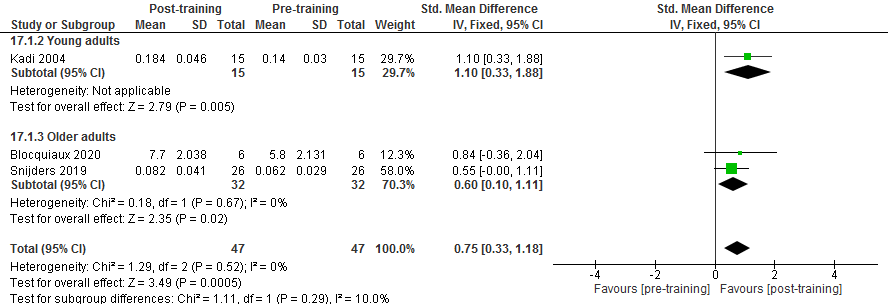


**2ST. SC in type I fibers after training.**


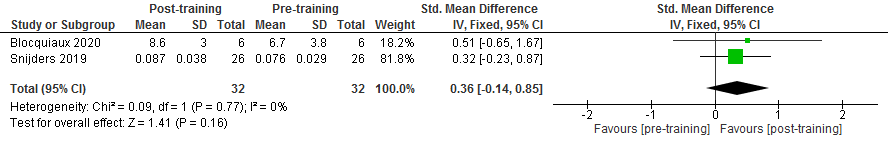


**2SU. SC in type II fibers after training.**


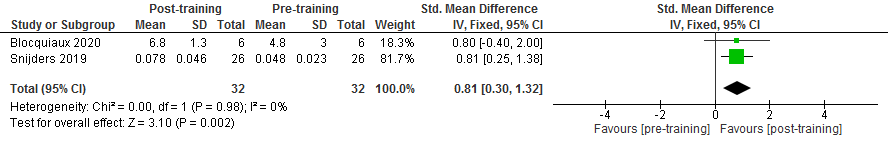


**2SW. SC in mixed fibers after detraining.**


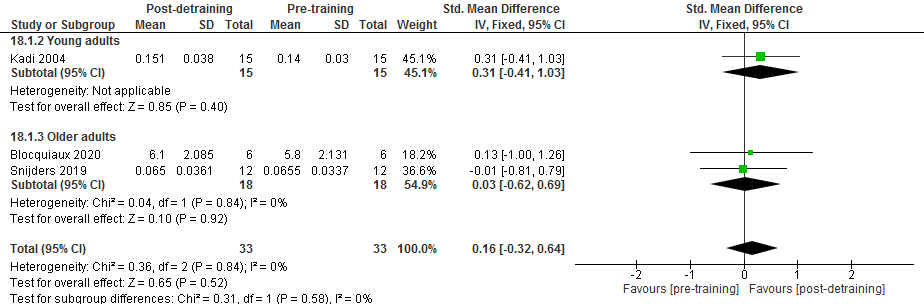


**2SX. SC in type I fibers after detraining.**


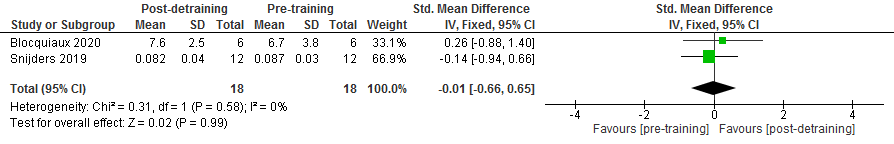


**2SY. SC in type II fibers after detraining.**


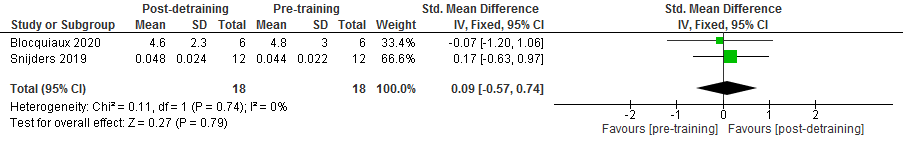


**Note:** Compared with baseline values, mean improvement of SCs after resistance training ranged from ~32.4% in mixed fibers to ~21.4% in type I and ~52% in type II fibers. However, compared with baseline values, the mean improvement of satellite cell numbers after a detraining period ranged from ~4% in mixed fibers to ~3.8% in type I and ~2.5% in type II fibers.
