# Supplementary material for: Myonuclear permanence in skeletal muscle memory: a systematic review and meta‐analysis of human and animal studies
Source: J Cachexia Sarcopenia Muscle. 2022 Aug 12;13(5):2276–97. doi: 10.1002/jcsm.13043 (PMC9530508; doi:10.1002/jcsm.13043)
Supplement: Supplementary file 4 — Figure S4. Meta‐analysis results for skeletal muscle responses in aging compared with young adults in human studies. [file JCSM-13-2276-s006.docx]

**Figure 4S. Meta-analysis results for skeletal muscle responses in aging compared with young adults in human studies.**

**4SA. Skeletal muscle CSA in mixed fibers.**


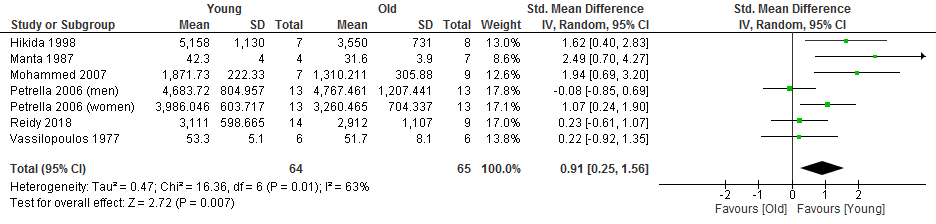


**4SB. Skeletal muscle CSA in type I fibers.**


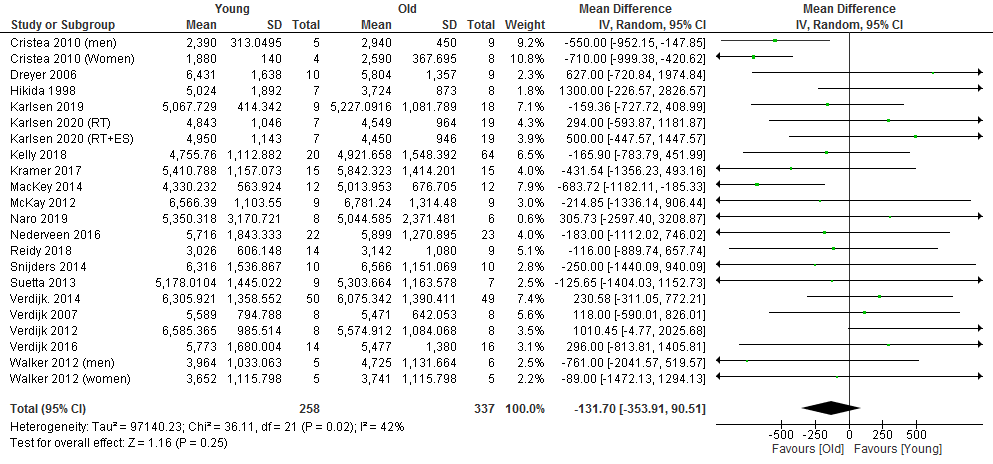


**4SC. Skeletal muscle CSA in type II fibers.**


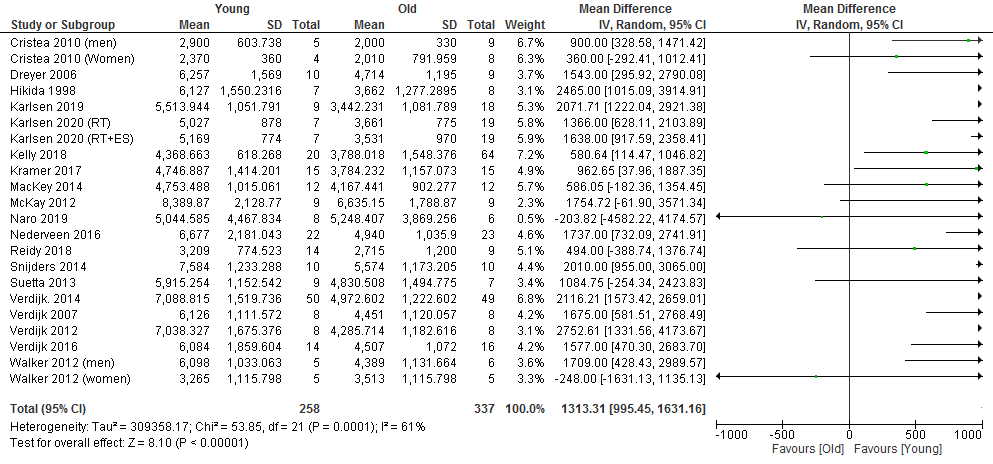


**Note:** The mean reduction in CSA in response to human aging ranged from ~-21.8% in mixed fibers to ~0.3% in type I and ~-32.1% in type II fibers.

**4SD. Myonuclear content in mixed fibers.**


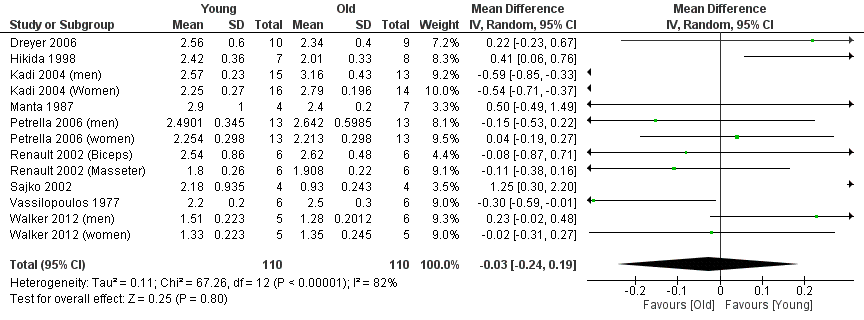


**4SE. Myonuclear content in type I fibers.**


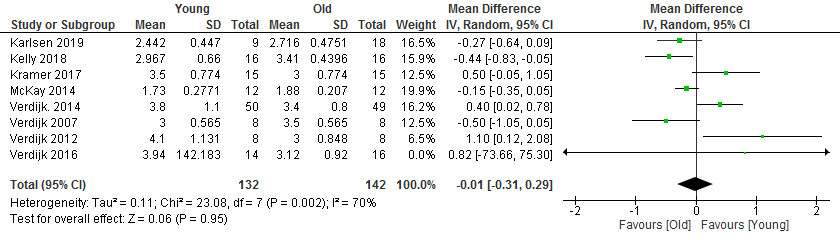


**4SF. Myonuclear content in type II fibers.**


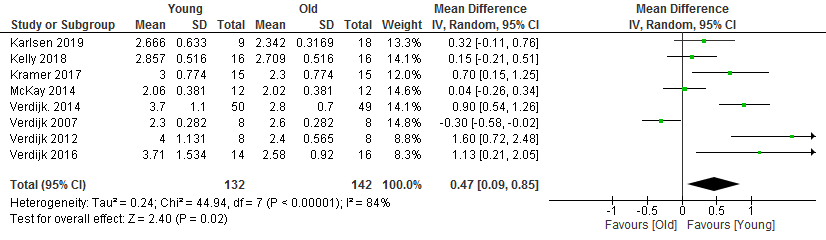


**Note:** The mean reduction in myonuclear content in response to human aging ranged from ~-0.1% in mixed fibers to ~-5.7% in type I and ~-22.8% in type II fibers.

**4SG. MND in mixed fibers.**


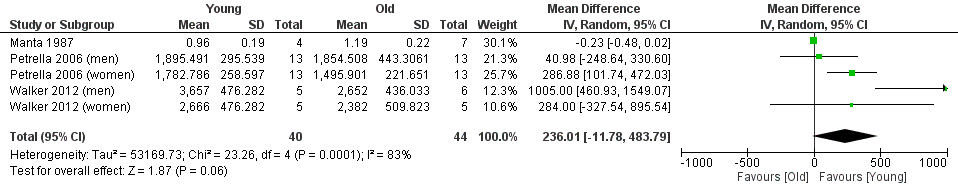


**4SH. MND in type I fibers.**


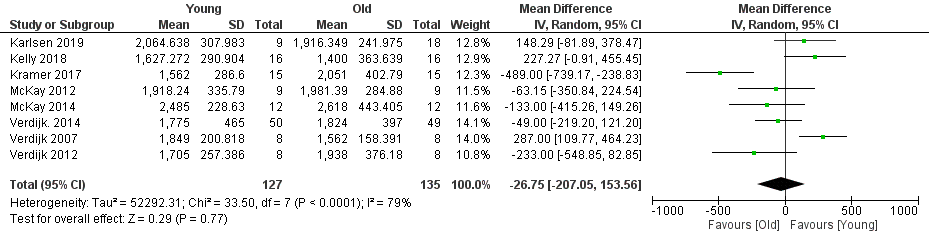


**4SI. MND in type II fibers.**


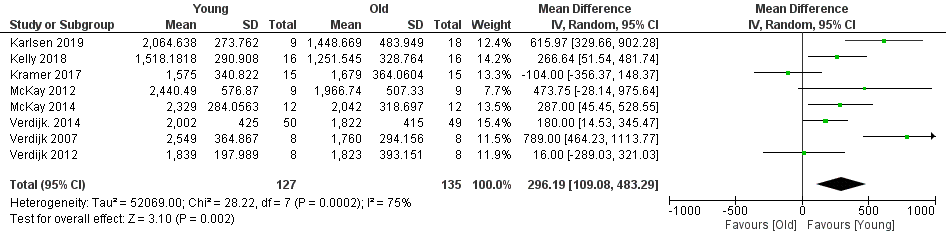


**Note:** The mean reduction in MND in response to human aging ranged from ~-10.4% in mixed fibers to ~1% in type I and ~-18.9% in type II fibers.

**4SJ. Satellite cells in mixed fibers.**


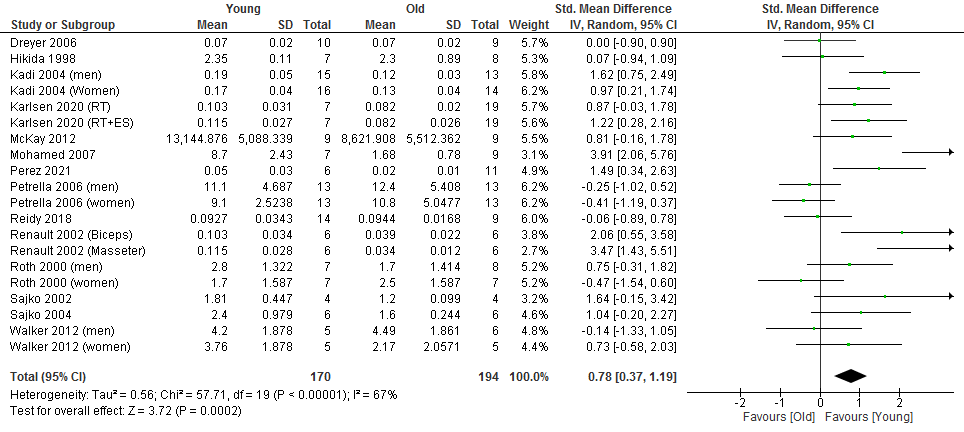


**4SK. Satellite cells in type I fibers.**


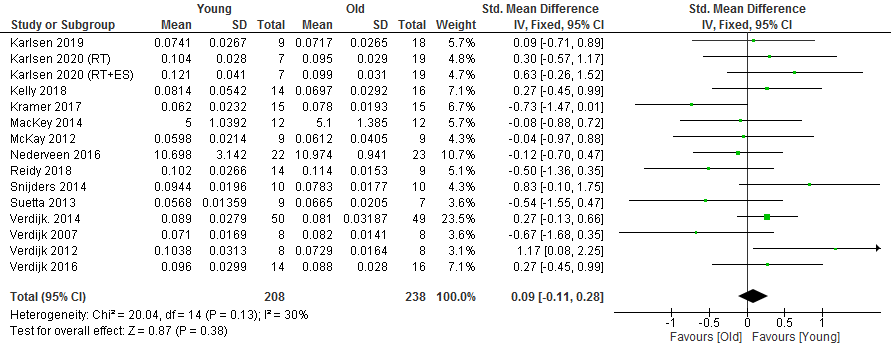


**4SL. Satellite cells in type II fibers.**


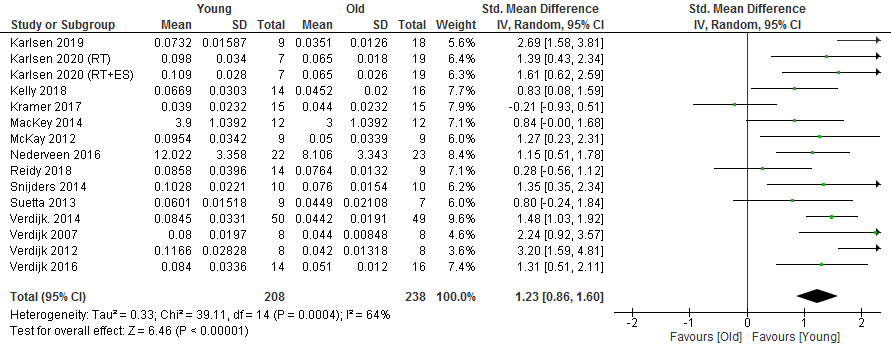


**Note:** The mean reduction in myonuclear content in response to human aging ranged from ~-68.5% in mixed fibers to ~-5% in type I and ~-63% in type II fibers.
