# Supplementary material for: Myonuclear permanence in skeletal muscle memory: a systematic review and meta‐analysis of human and animal studies
Source: J Cachexia Sarcopenia Muscle. 2022 Aug 12;13(5):2276–97. doi: 10.1002/jcsm.13043 (PMC9530508; doi:10.1002/jcsm.13043)
Supplement: Supplementary file 5 — Figure S5. Meta‐analysis results for skeletal muscle responses to hypertrophy in animal studies. [file JCSM-13-2276-s002.docx]

**Figure 5S. Meta-analysis results for skeletal muscle responses to hypertrophy in animal studies.**

**5SA. Skeletal muscle CSA after training.**


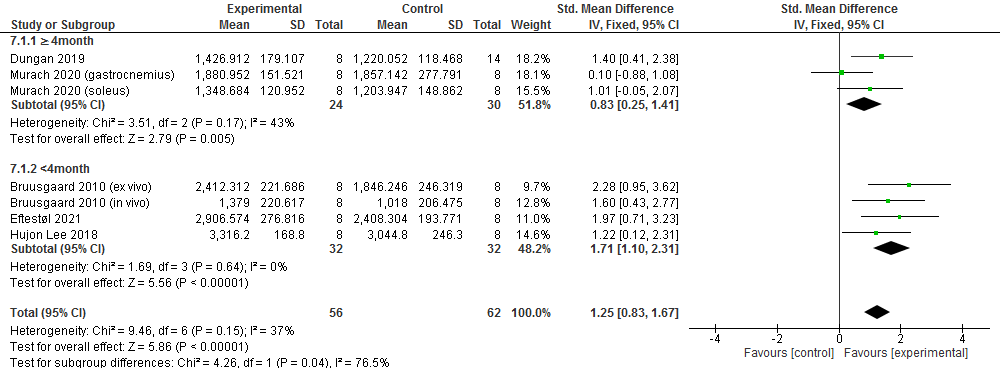


**5SB. Skeletal muscle CSA after detraining.**


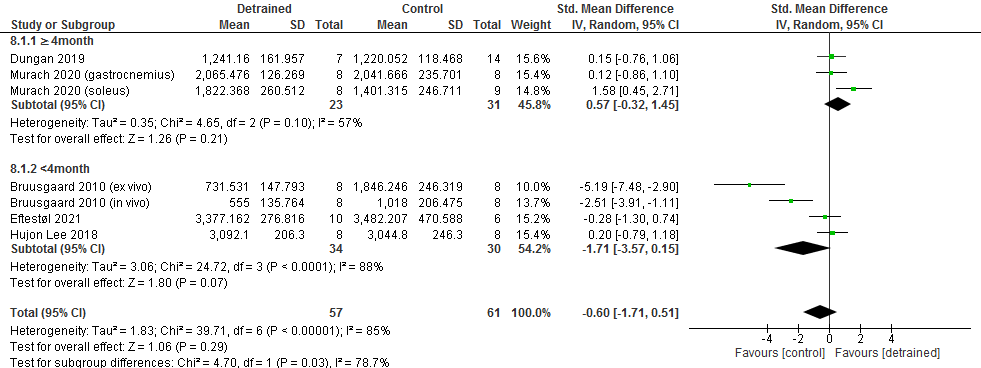


**Note:** CSA responded to hypertrophy with a mean improvement of ~+18% after training and an average reduction of ~-10.63% after detraining. Subgroup analysis showed that CSA in mature animals improves to a greater extent in response to overload-induced hypertrophy (P= 0.04).

**5SC. Myonuclear content in CSA after training.**


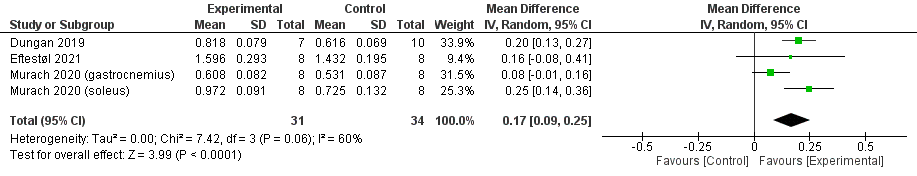


**5SD. Myonuclear content in CSA after detraining.**


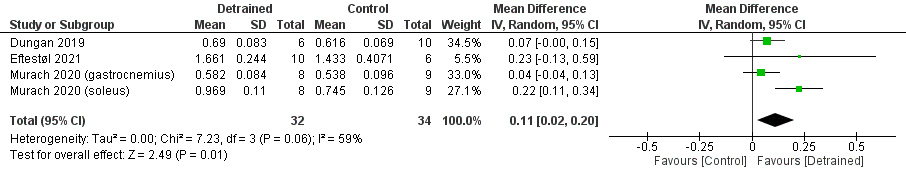


**Note:** Myonuclear content responded to hypertrophy with a mean improvement of ~+23.2% after training and an average improvement of ~+16.5% after detraining.

**5SE. Myonuclear content in single muscle fiber after training.**


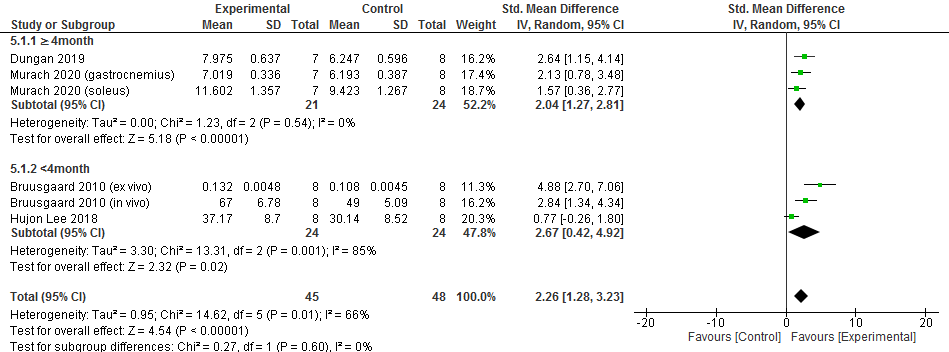


**5SF. Myonuclear content in single muscle fiber after detraining.**


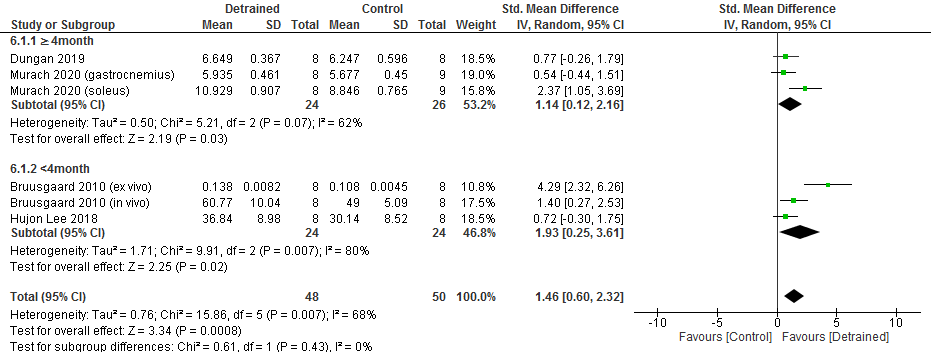


**Note:** Myonuclear content responded to hypertrophy with a mean improvement of ~+24.4% after training and an average improvement of ~+18.1% after detraining.
