# Supplementary material for: Myonuclear permanence in skeletal muscle memory: a systematic review and meta‐analysis of human and animal studies
Source: J Cachexia Sarcopenia Muscle. 2022 Aug 12;13(5):2276–97. doi: 10.1002/jcsm.13043 (PMC9530508; doi:10.1002/jcsm.13043)

**Figure 6S. Meta-analysis results for skeletal muscle responses to atrophy in animal studies.**

**6SA. Skeletal muscle CSA**


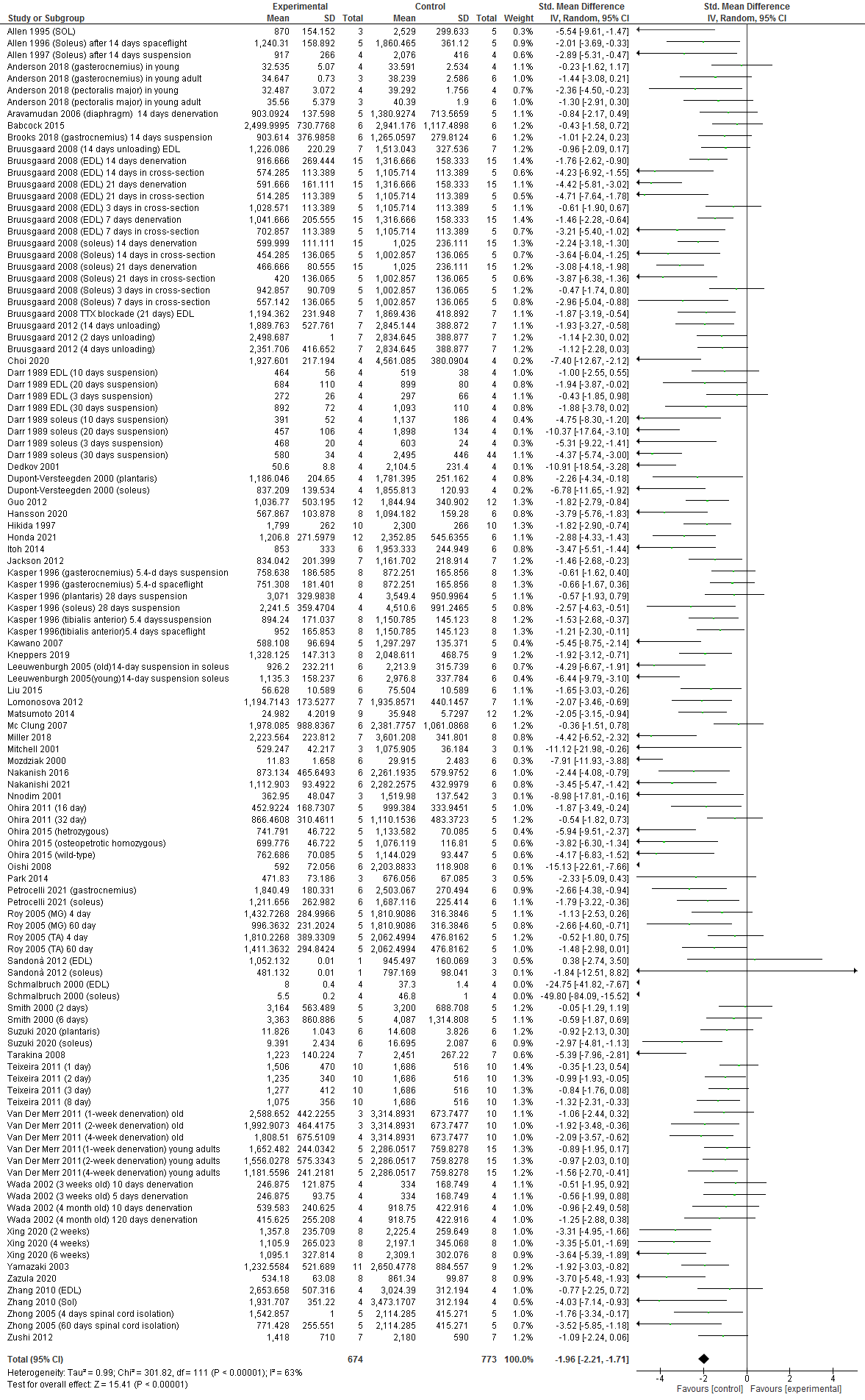


**6SB. Subgroup analysis of skeletal muscle CSA based on different muscles.**


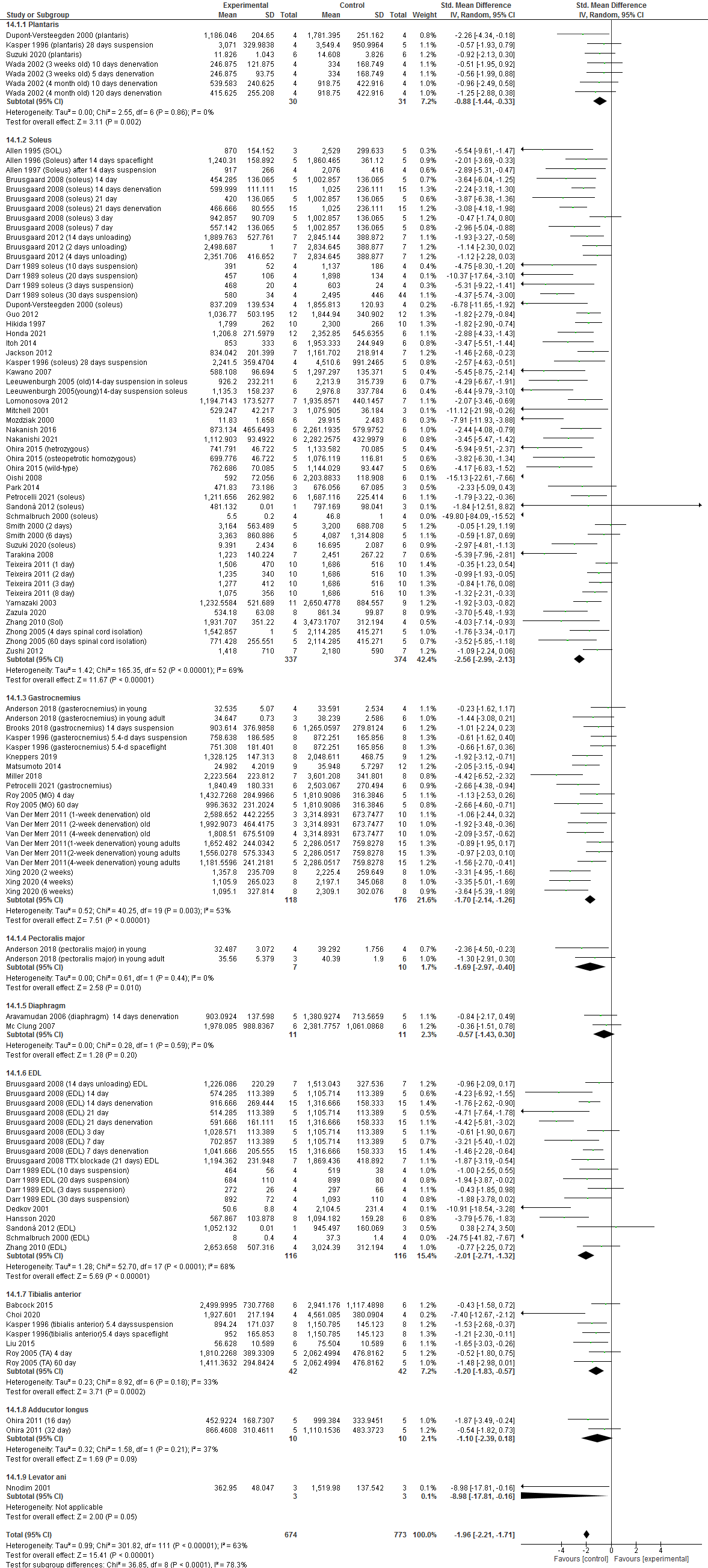


**6SC. Subgroup analysis of skeletal muscle CSA based on different intervention periods.**


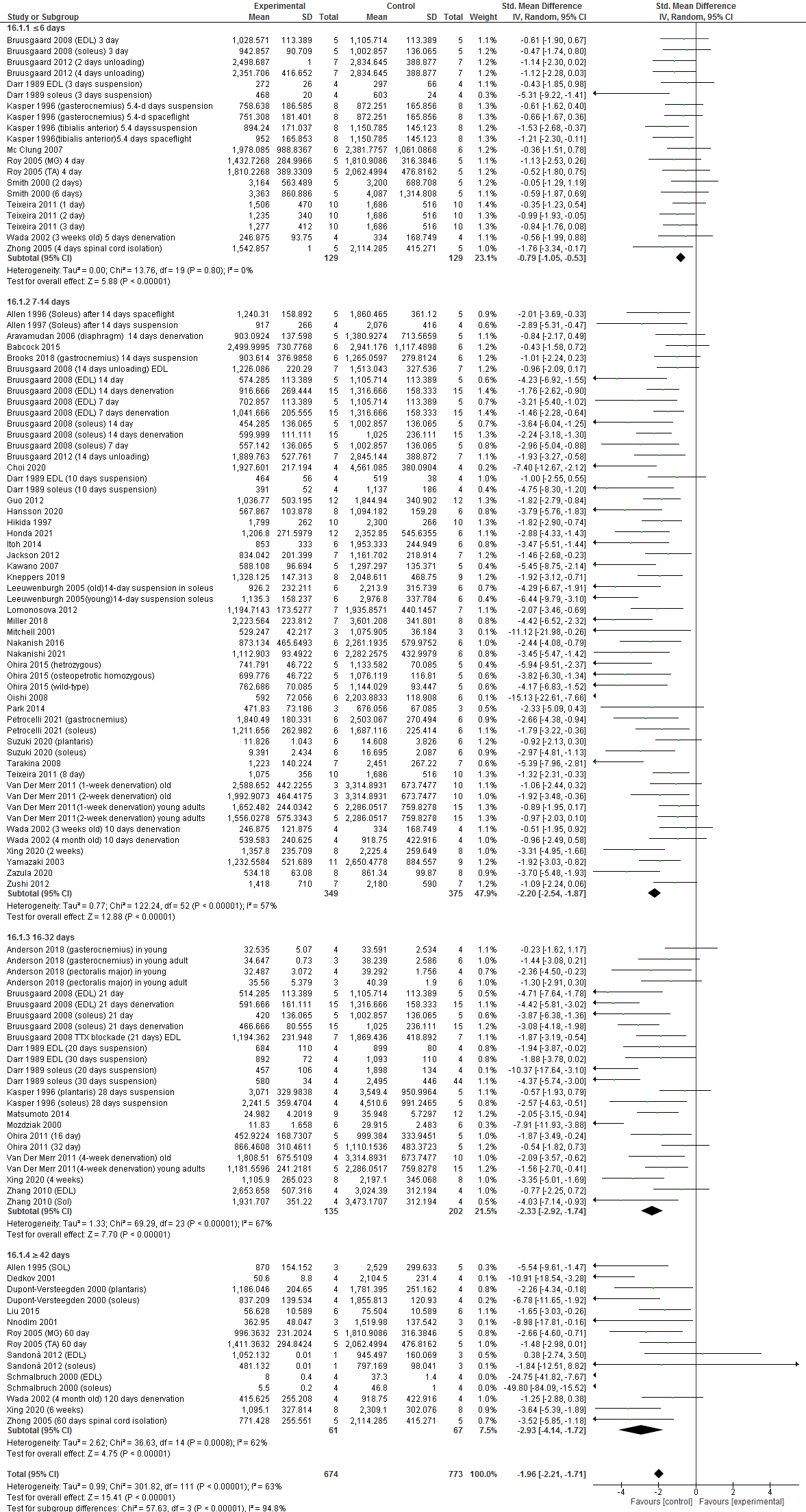


**6SD. Subgroup analysis of skeletal muscle CSA based on different intervention methods.**


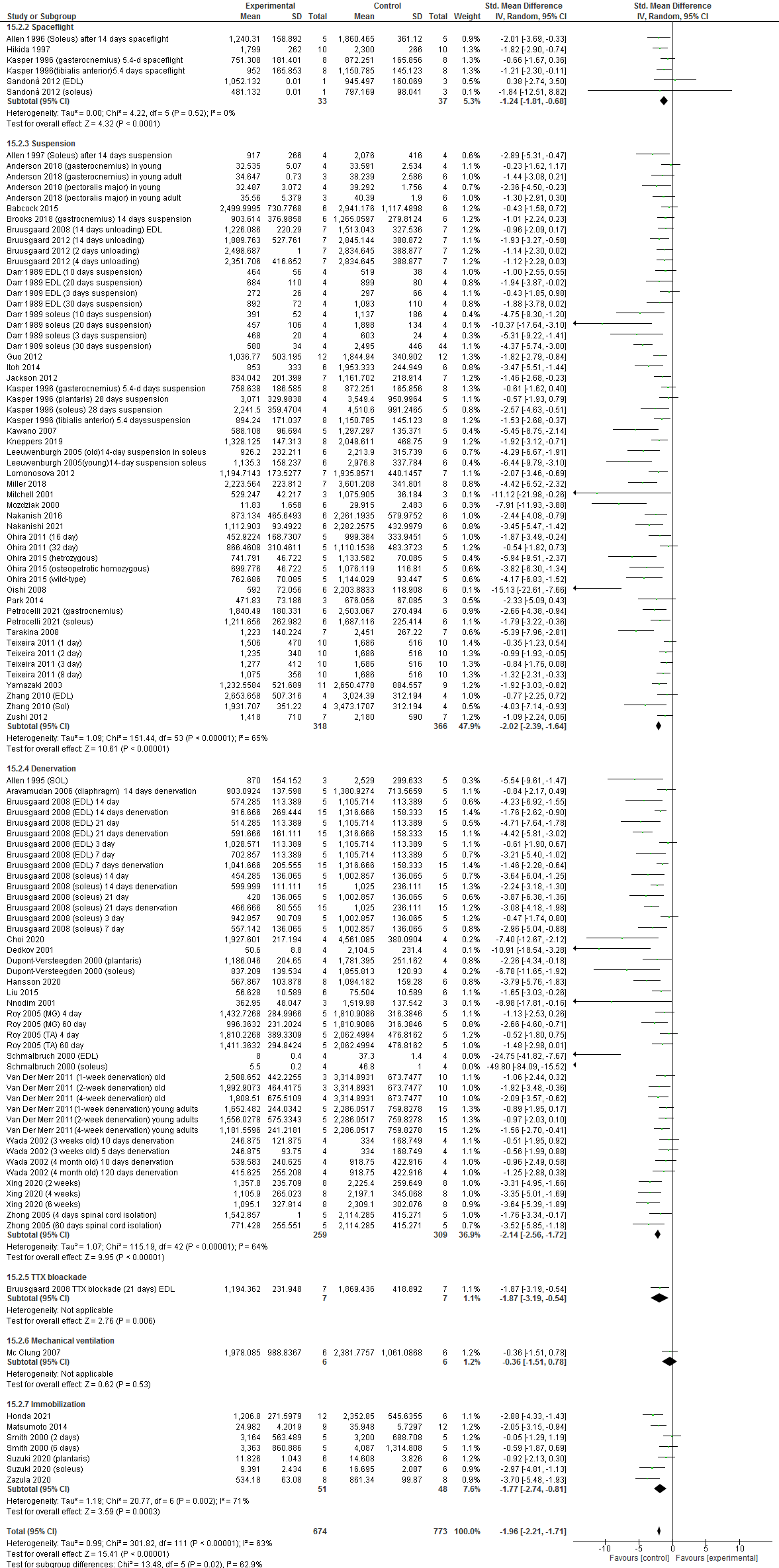


**6SE. Subgroup analysis of myonuclear content in CSA based on different intervention methods.**


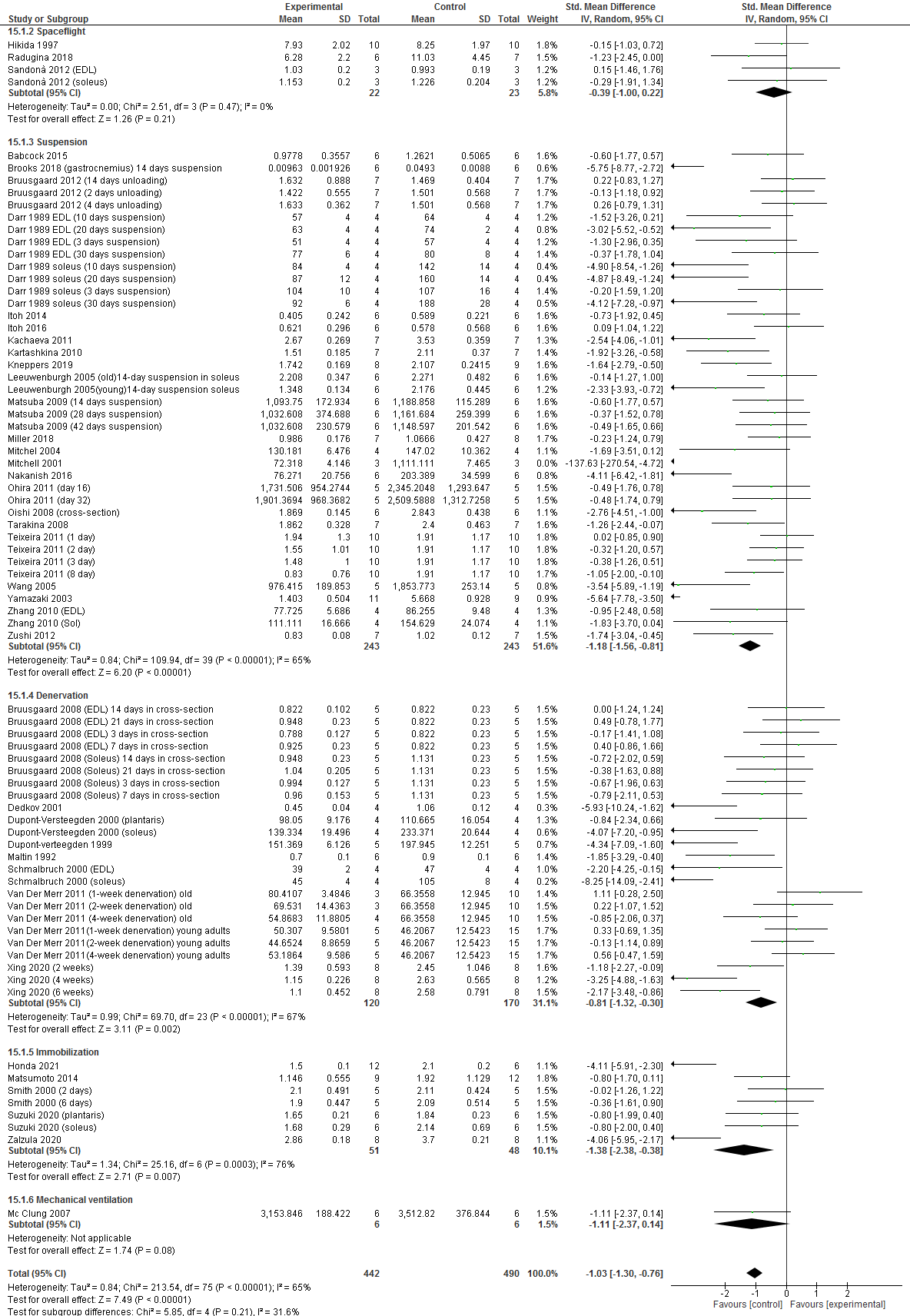


**6SF. Myonuclear content based on %CSA reduction.**


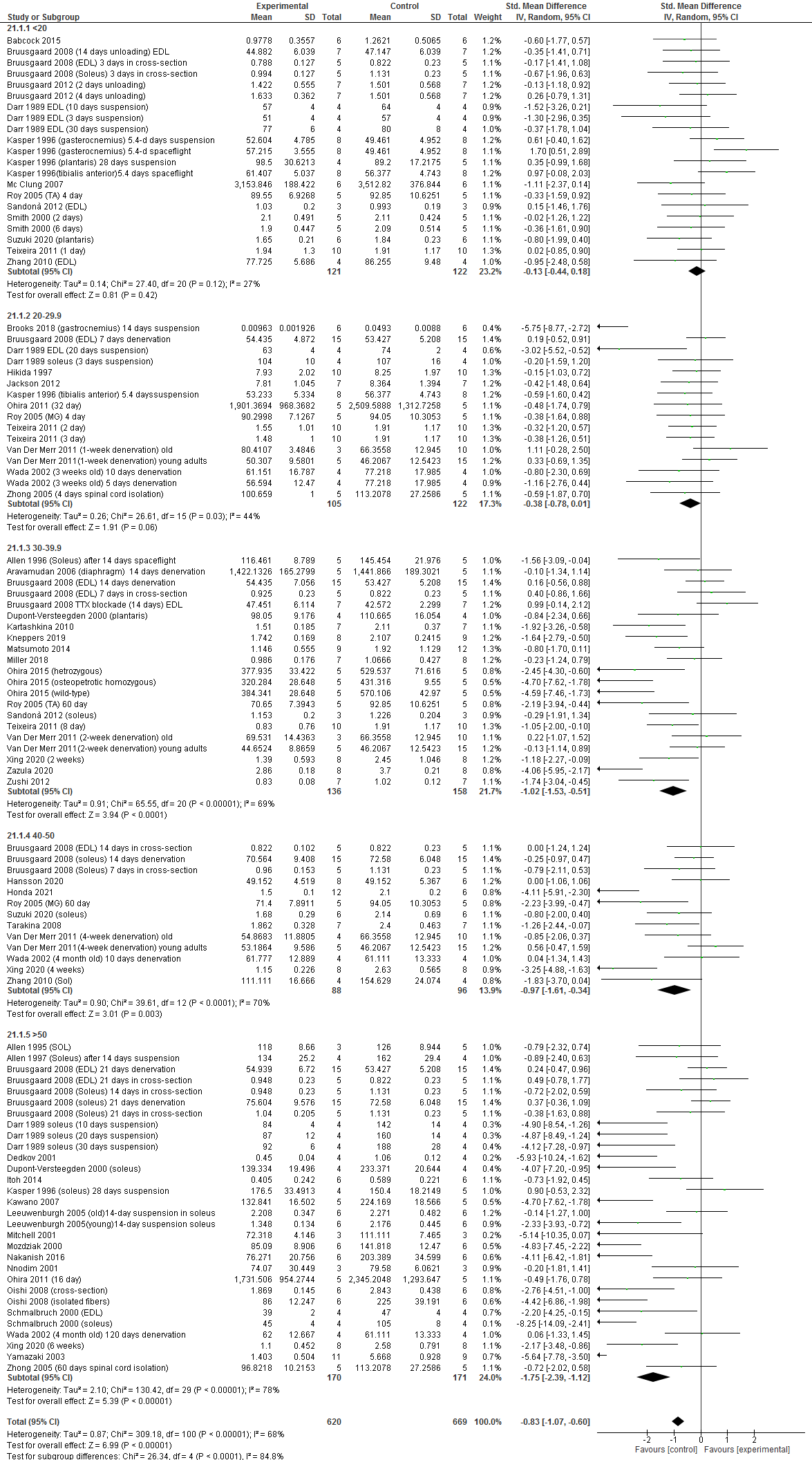


**6SG. Subgroup analysis of satellite cells in CSA based on different intervention methods.**


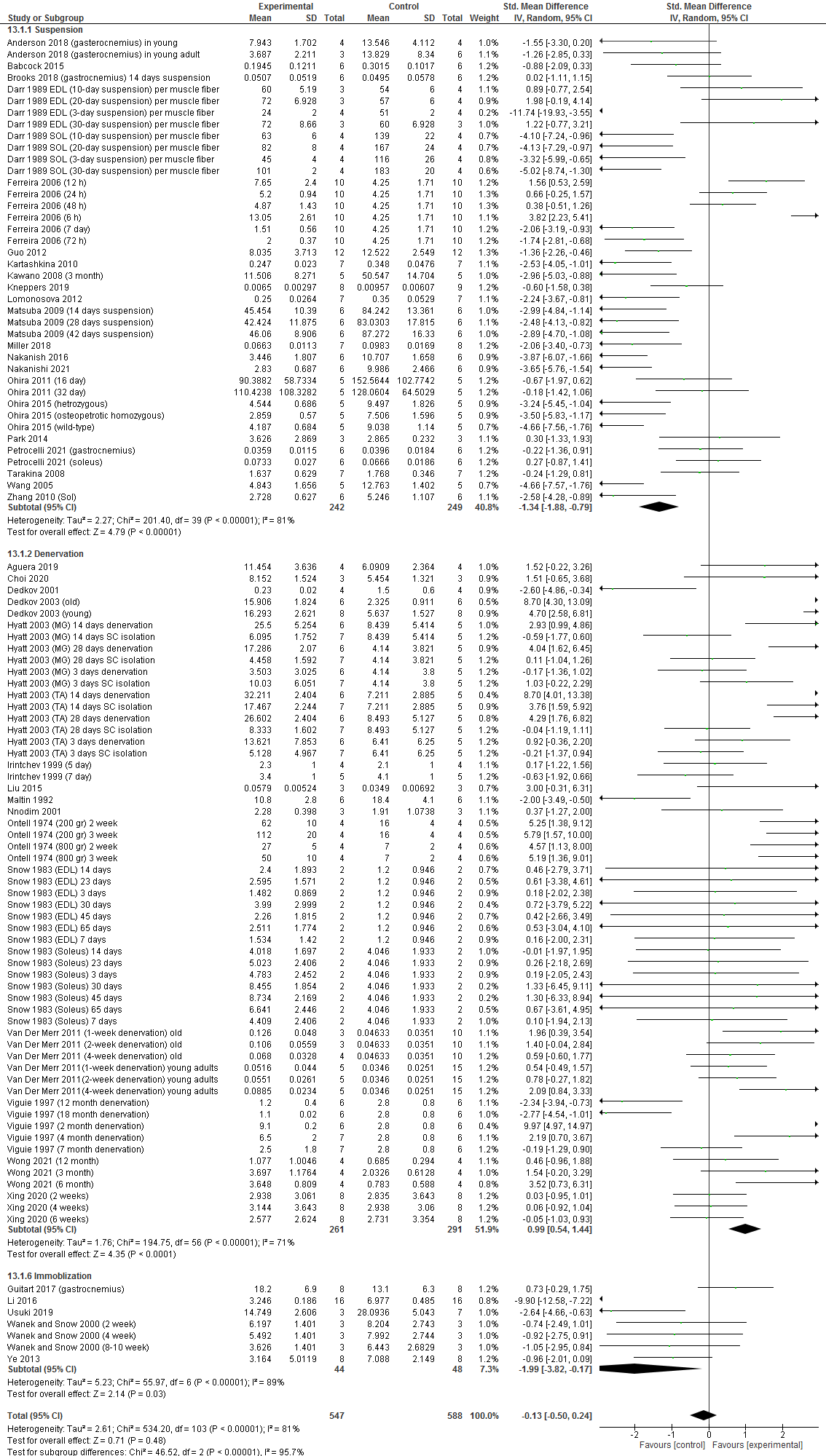


**6SH. Myonuclear content in CSA.**


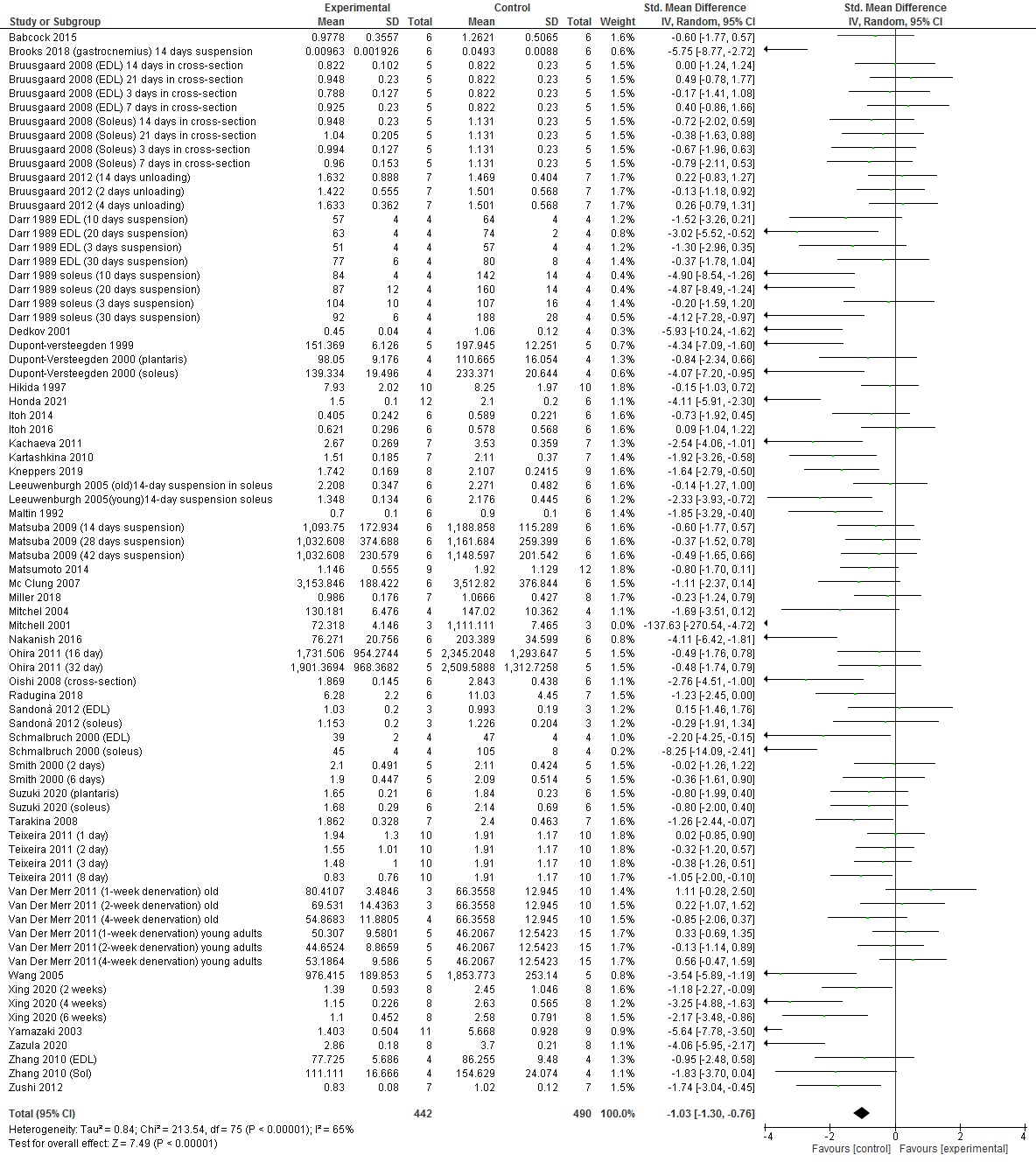


**6SI. Subgroup analysis of myonuclear content in CSA based on different muscles.**


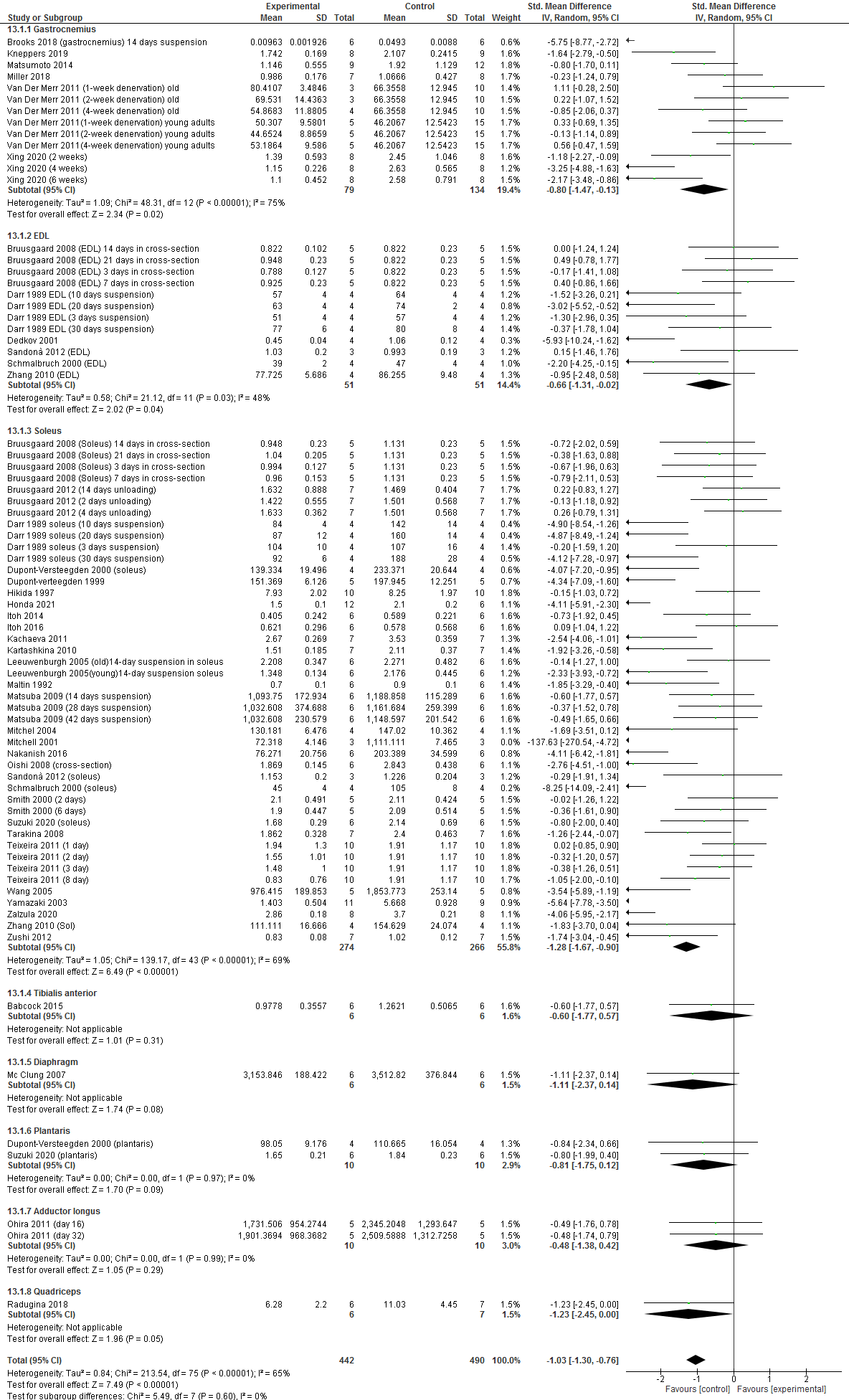


**6SJ. Subgroup analysis of myonuclear content in CSA based on different intervention periods.**


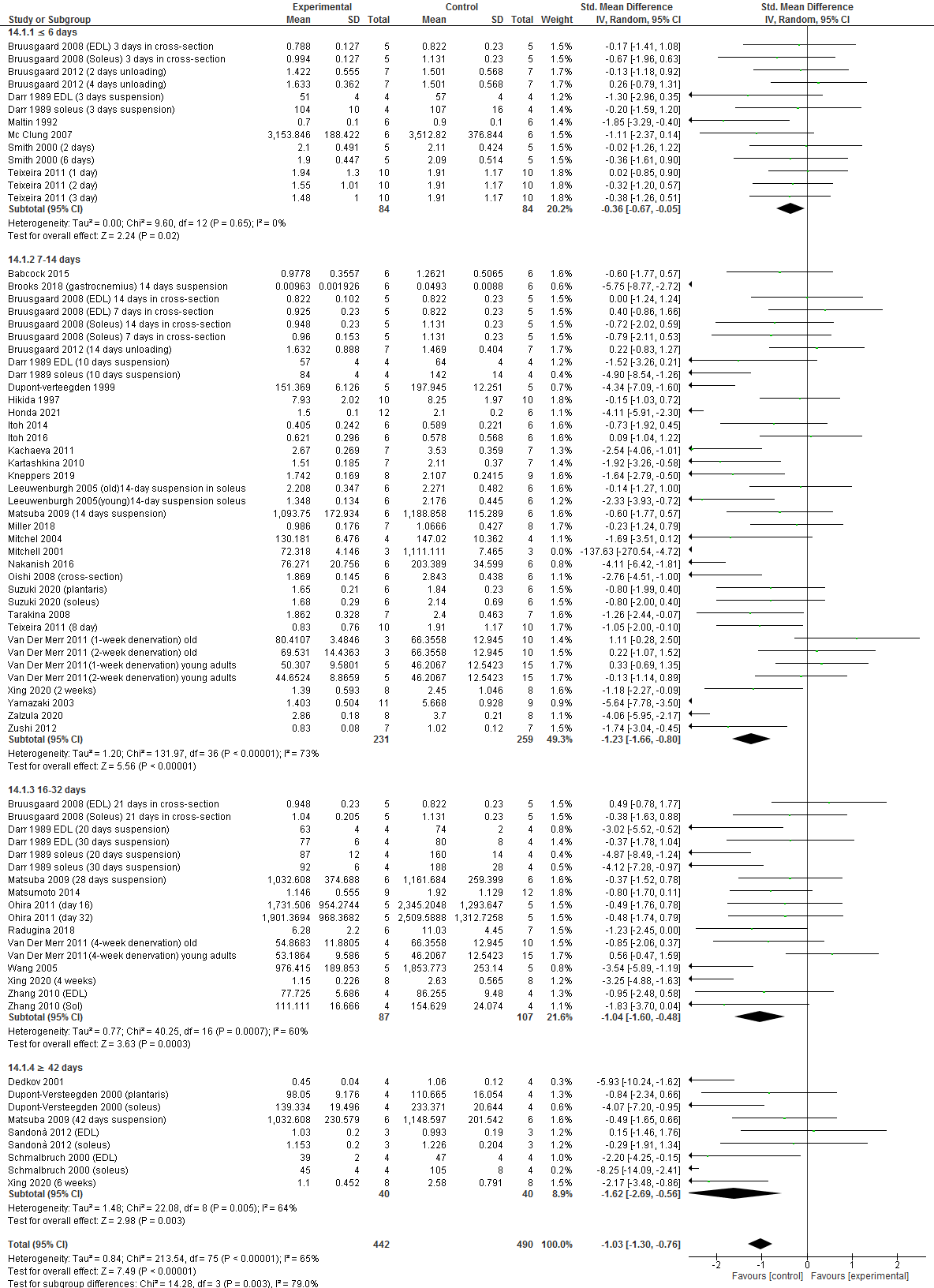


**6SK. Myonuclear content in single muscle fiber.**


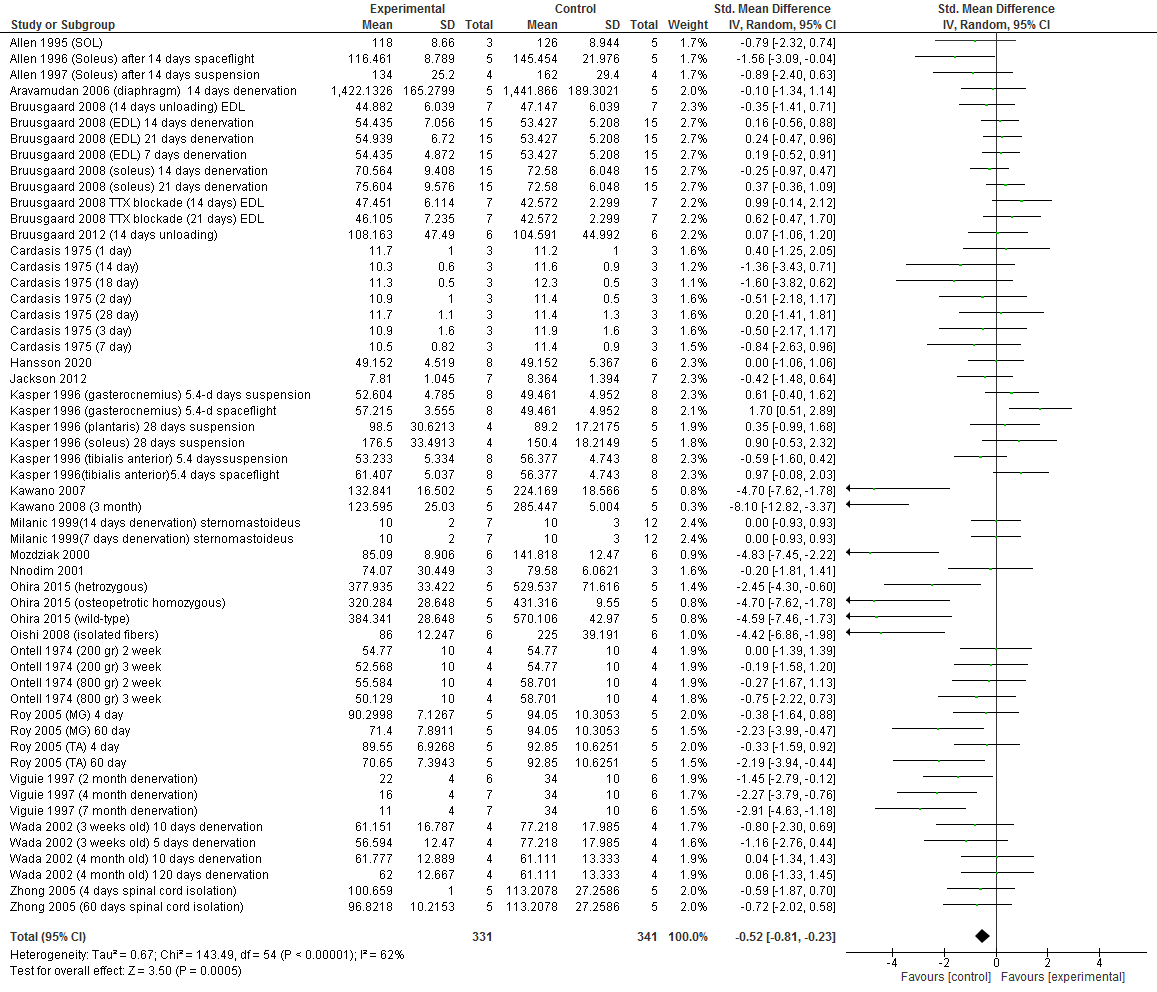


**6SL. Subgroup analysis of myonuclear content in single muscle fiber based on different muscles.**


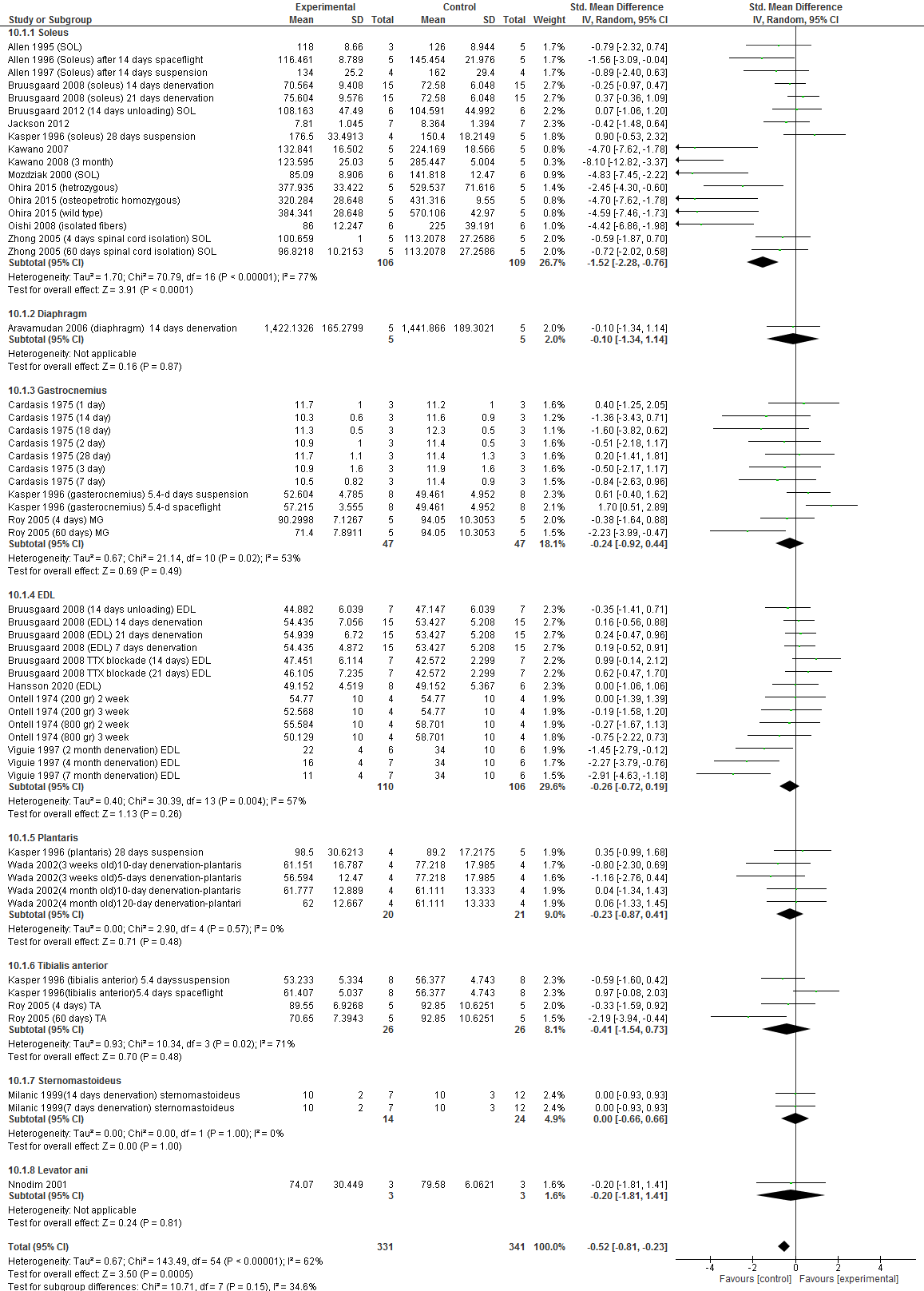


**6SM. Subgroup analysis of myonuclear content in single muscle fiber based on different intervention periods.**


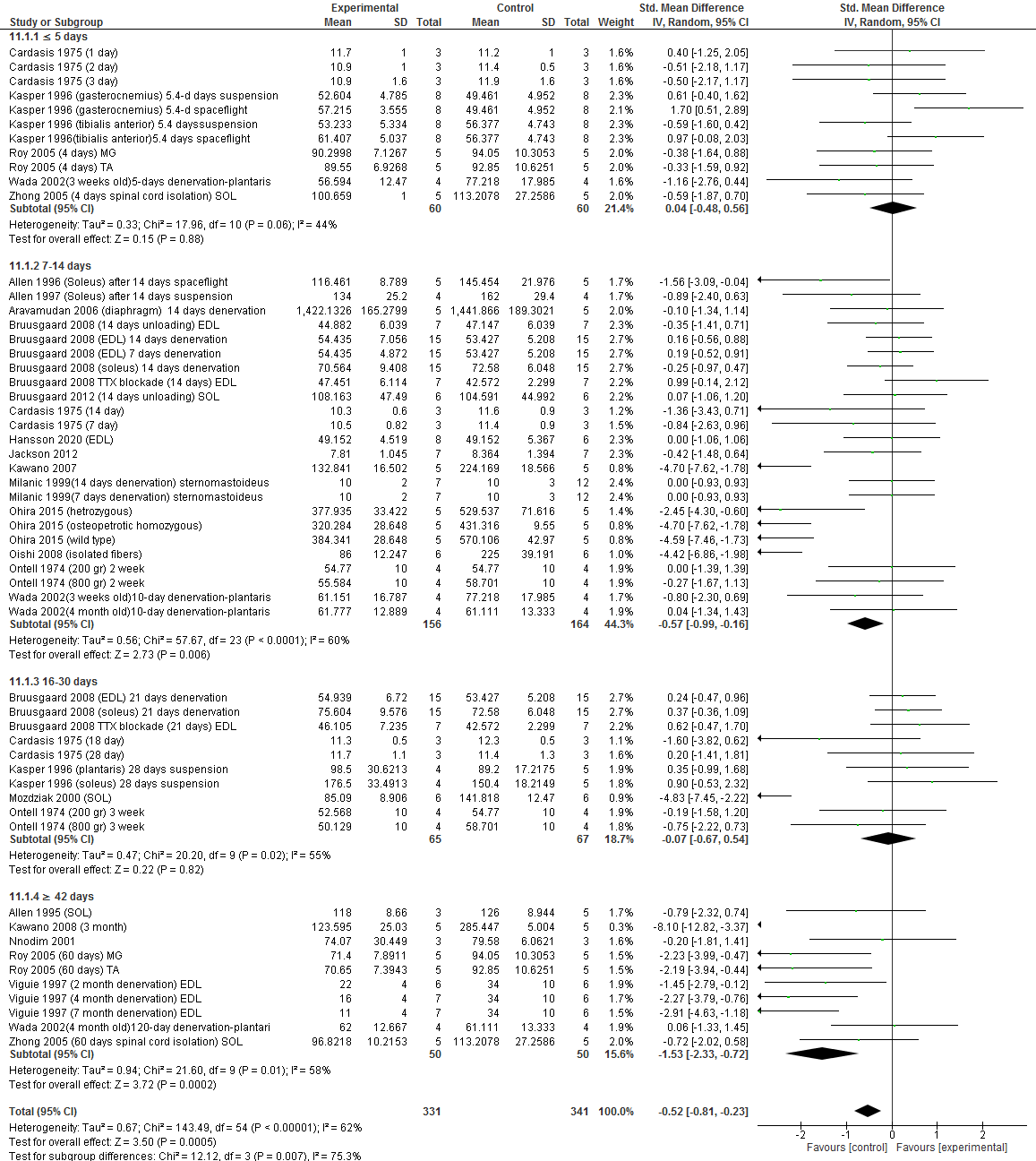


**6SN. Subgroup analysis of myonuclear content in single muscle fiber based on different intervention methods.**


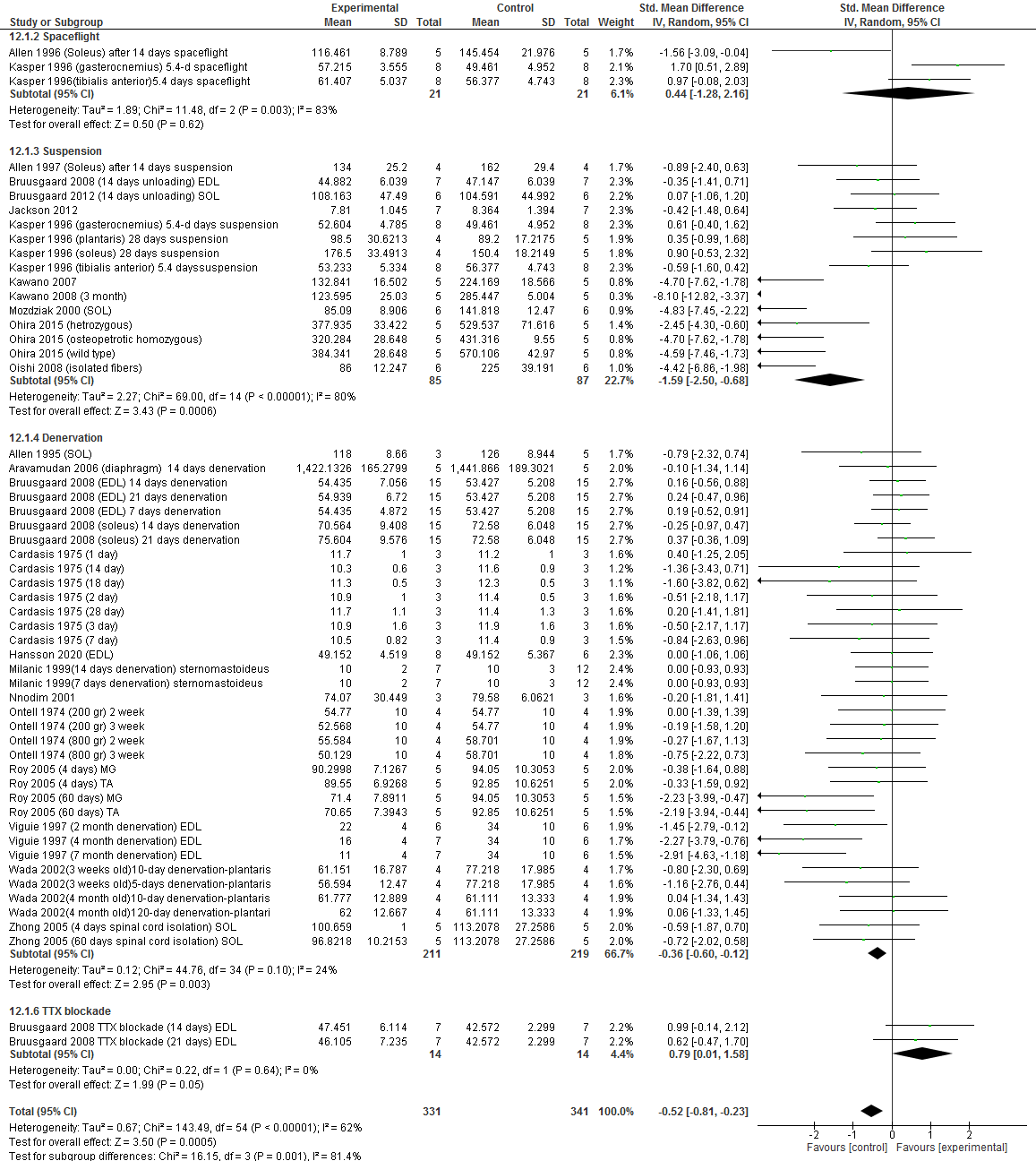


**6SO. Satellite cell in CSA.**


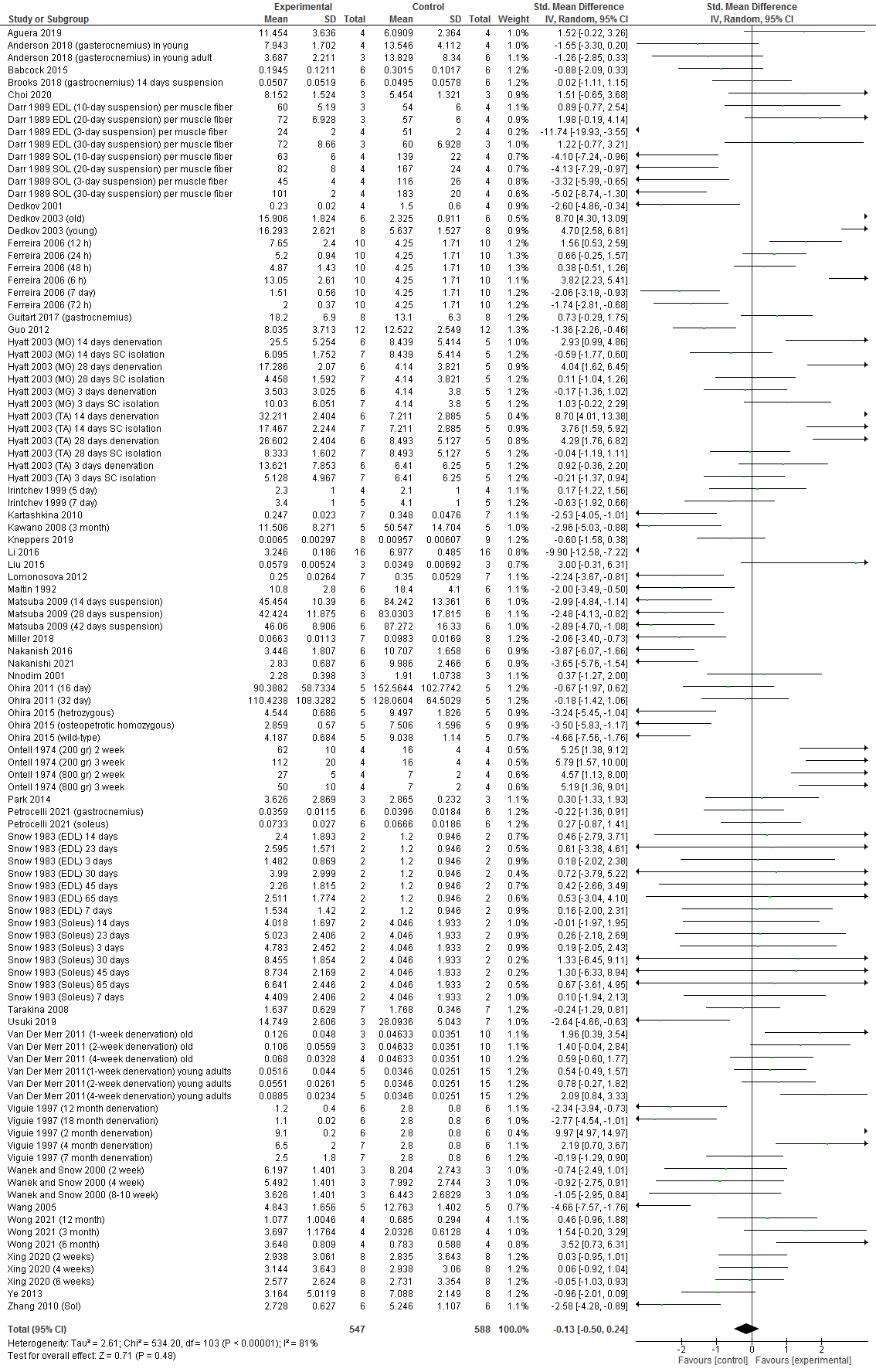


**6SP. Subgroup analysis of satellite cells in CSA based on different muscles.**


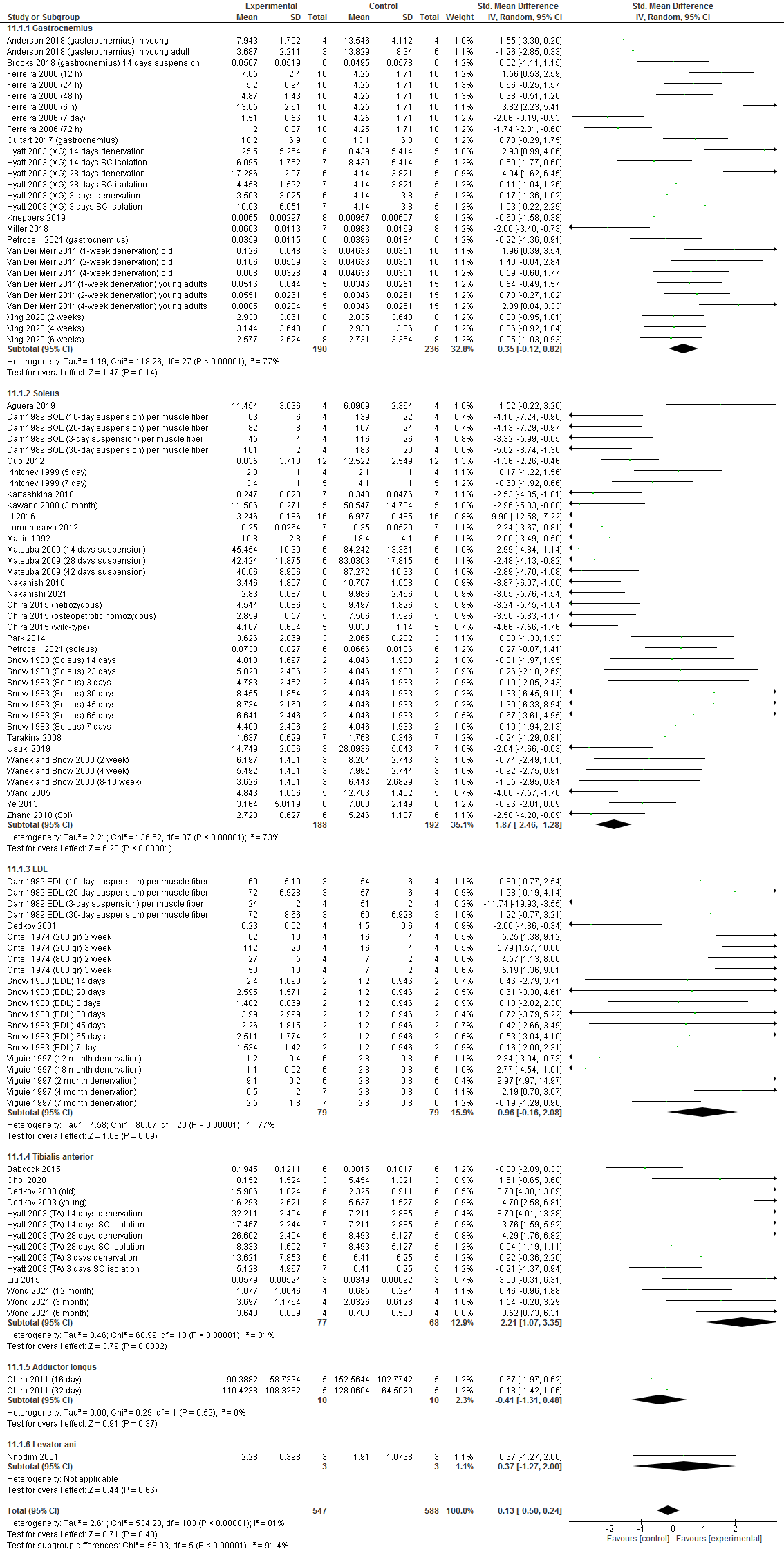


**6SQ. Subgroup analysis of satellite cells in CSA based on different intervention periods.**


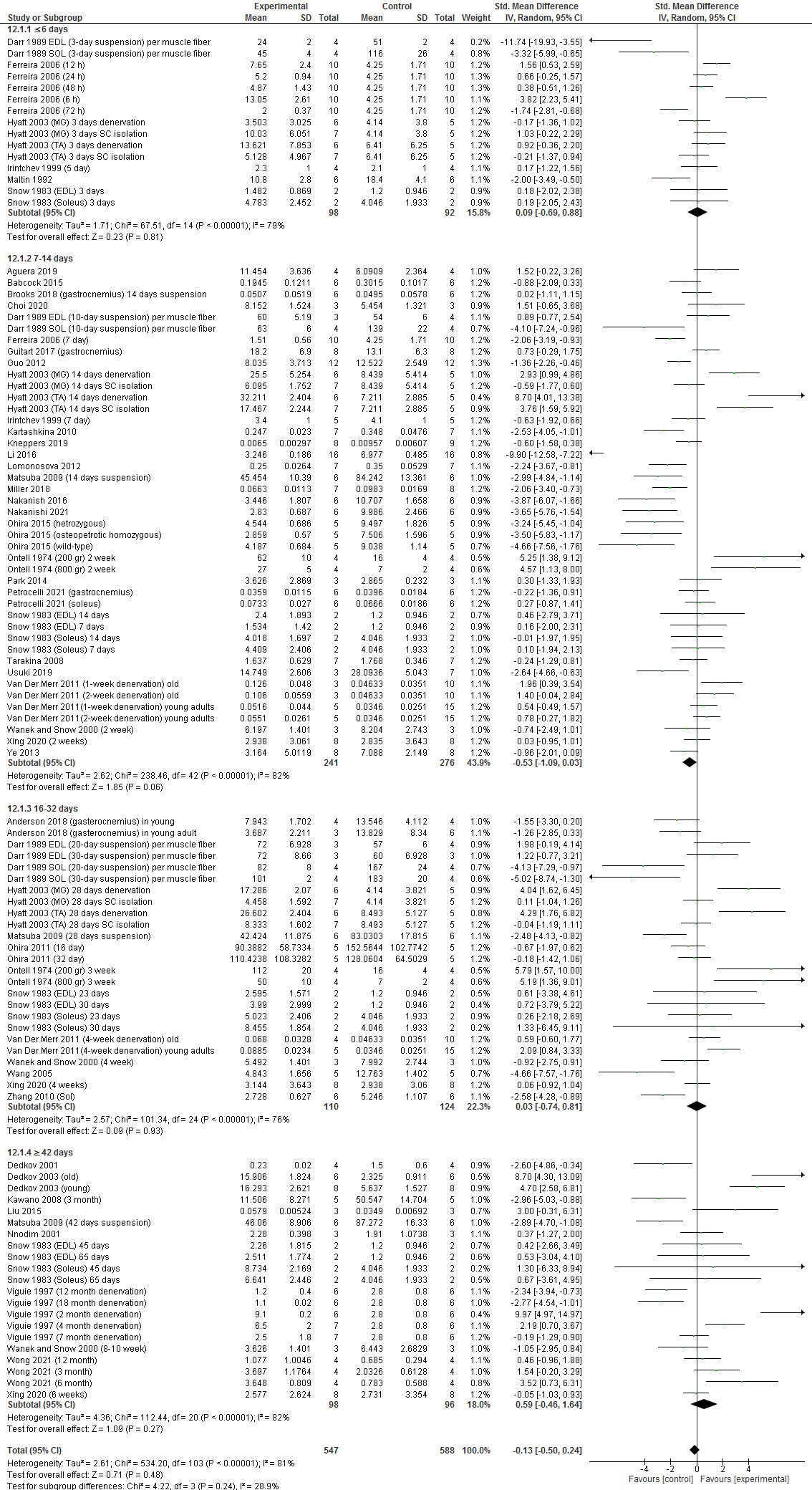

Supplement: Supplementary file 6 — Figure S6. Meta‐analysis results for skeletal muscle responses to atrophy in animal studies. [file JCSM-13-2276-s008.docx]
