# Supplementary material for: Myonuclear permanence in skeletal muscle memory: a systematic review and meta‐analysis of human and animal studies
Source: J Cachexia Sarcopenia Muscle. 2022 Aug 12;13(5):2276–97. doi: 10.1002/jcsm.13043 (PMC9530508; doi:10.1002/jcsm.13043)
Supplement: Supplementary file 7 — Figure S7A. Funnel plots for publication bias on skeletal muscle responses to hypertrophy in human studies after training. Figure S7B. Funnel plots for publication bias on skeletal muscle responses to hypertrophy in human studies after detraining. Figure S7C. Funnel plots for publication bias on skeletal muscle responses to atrophy in human studies. Figure S7D. Funnel plots for publication bias on skeletal muscle responses in aging compared with young adults. Figure S7E. Funnel plots for publication bias on skeletal muscle responses to hypertrophy in animal studies. Figure S7F. Funnel plots for publication bias on skeletal muscle responses to atrophy in animal studies. [file JCSM-13-2276-s007.docx]

**Figure 7SA.** Funnel plots for publication bias on skeletal muscle responses to hypertrophy in human studies after training.

|  |  | **Funnel plot** |  |
| --- | --- | --- | --- |
| **Standard error** | CSA after training  (Mixed fibers) | CSA after training  (type I fibers) | CSA after training  (type II fibers) |
|  |  |  |  |
|  | Myonuclear content after training (Mixed fibers) | Myonuclear content after training (type I fibers) | Myonuclear content after training (type II fibers) |
|  |  |  |  |
|  | Myonuclear domain after training (Mixed fibers) | Myonuclear domain after training (type I fibers) | Myonuclear domain after training (type II fibers) |
|  |  | **** |  |
|  | Satellite cell after training (Mixed fibers) | Satellite cell after training  (type I fibers) | Satellite cell after training  (type II fibers) |
|  | **** |  |  |
|  | 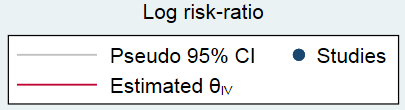 | | |

**Figure 7SB.** Funnel plots for publication bias on skeletal muscle responses to hypertrophy in human studies after detraining.

|  |  | **Funnel plot** |  |
| --- | --- | --- | --- |
| **Standard error** | CSA after detraining  (Mixed fibers) | CSA after detraining  (type I fibers) | CSA after detraining  (type II fibers) |
|  |  |  | **** |
|  | Myonuclear content after detraining (Mixed fibers) | Myonuclear content after detraining (type I fibers) | Myonuclear content after detraining (type II fibers) |
|  |  |  |  |
|  | Myonuclear domain after detraining (Mixed fibers) | Myonuclear domain after detraining (type I fibers) | Myonuclear domain after detraining (type II fibers) |
|  |  |  | **** |
|  | Satellite cell after detraining (Mixed fibers) | Satellite cell after detraining  (type I fibers) | Satellite cell after detraining  (type II fibers) |
|  |  | **** | **** |
|  | 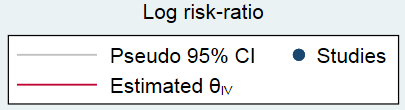 | | |

**Figure 7SC.** Funnel plots for publication bias on skeletal muscle responses to atrophy in human studies.

|  |  | **Funnel plot** |  |
| --- | --- | --- | --- |
| **Standard error** | CSA (Mixed fibers) | CSA (type I fibers) | CSA (type II fibers) |
|  | ****** | ****** | ****** |
|  | Myonuclear content  (Mixed fibers) | Myonuclear content  (type I fibers) | Myonuclear content  (type II fibers) |
|  | ****** | ****** | ****** |
|  | Myonuclear domain  (Mixed fibers) | Myonuclear domain  (type I fibers) | Myonuclear domain  (type II fibers) |
|  | ****** | ****** | ****** |
|  | Satellite cell (Mixed fibers) | Satellite cell (type I fibers) | Satellite cell (type II fibers) |
|  | ****** | ****** | ****** |
|  | 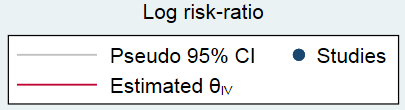 | | |

**Figure 7SD.** Funnel plots for publication bias on skeletal muscle responses in aging compared with young adults.

|  |  | **Funnel plot** |  |
| --- | --- | --- | --- |
| **Standard error** | CSA (Mixed fibers) | CSA (type I fibers) | CSA (type II fibers) |
|  |  |  |  |
|  | Myonuclear content  (Mixed fibers) | Myonuclear content  (type I fibers) | Myonuclear content  (type II fibers) |
|  | ****** |  |  |
|  | Myonuclear domain  (Mixed fibers) | Myonuclear domain  (type I fibers) | Myonuclear domain  (type II fibers) |
|  | ****** | ****** | ****** |
|  | Satellite cell (Mixed fibers) | Satellite cell (type I fibers) | Satellite cell (type II fibers) |
|  | ****** | ****** | ****** |
|  | 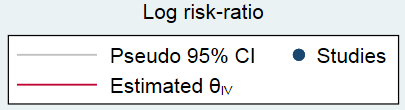 | | |

**Figure 7SE.** Funnel plots for publication bias on skeletal muscle responses to hypertrophy in animal studies.

|  | **Funnel plot** | |
| --- | --- | --- |
| **Standard error** | CSA (exercise *vs* control) | CSA (control *vs* detraining) |
|  |  |  |
|  | Myonuclear content in the whole cross-section  (exercise vs. control) | Myonuclear content in the whole cross-section  (control vs. detraining) |
|  |  |  |
|  | Myonuclear content in single muscle fiber  (exercise vs. control) | Myonuclear content in single muscle fiber  (control vs. detraining) |
|  |  |  |
|  | 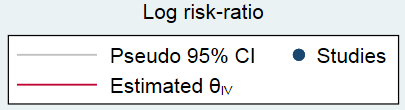 | |

**Figure 7SF.** Funnel plots for publication bias on skeletal muscle responses to atrophy in animal studies.

|  | **Funnel plot** | |
| --- | --- | --- |
| **Standard error** | CSA | Myonuclear content in the whole cross-section |
|  |  |  |
|  | Myonuclear content in single muscle fiber | Satellite cell |
|  |  |  |
|  | 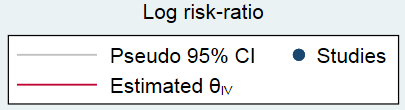 | |
