# Supplementary material for: Myonuclear permanence in skeletal muscle memory: a systematic review and meta‐analysis of human and animal studies
Source: J Cachexia Sarcopenia Muscle. 2022 Aug 12;13(5):2276–97. doi: 10.1002/jcsm.13043 (PMC9530508; doi:10.1002/jcsm.13043)
Supplement: Supplementary file 8 — Data S1. Supporting Information [file JCSM-13-2276-s001.docx]

**Supplementary references**

S1. Hyatt J-PK, Roy RR, Baldwin KM, Edgerton VR. Nerve activity-independent regulation of skeletal muscle atrophy: role of MyoD and myogenin in satellite cells and myonuclei. *American Journal of Physiology-Cell Physiology.* 2003;285(5):C1161-C1173.

S2. Ontell M. Muscle satellite cells: a validated technique for light microscopic identification and a quantitative study of changes in their population following denervation. *The Anatomical Record.* 1974;178(2):211-227.

S3. Cardasis CA, Cooper GW. A method for the chemical isolation of individual muscle fibers and its application to a study of the effect of denervation on the number of nuclei per muscle fiber. *Journal of Experimental Zoology.* 1975;191(3):333-345.

S4. Snow MH. A quantitative ultrastructural analysis of satellite cells in denervated fast and slow muscles of the mouse. *The Anatomical Record.* 1983;207(4):593-604.

S5. Maltin CA, Delday MI. Satellite cells in innervated and denervated muscles treated with clenbuterol. *Muscle & Nerve: Official Journal of the American Association of Electrodiagnostic Medicine.* 1992;15(8):919-925.

S6. Irintchev A, Zeschnigk M, Starzinski‐Powitz A, Wernig A. Expression pattern of M‐cadherin in normal, denervated, and regenerating mouse muscles. *Developmental Dynamics.* 1994;199(4):326-337.

S7. Allen D, Monke S, Talmadge R, Roy R, Edgerton V. Plasticity of myonuclear number in hypertrophied and atrophied mammalian skeletal muscle fibers. *Journal of Applied Physiology.* 1995;78(5):1969-1976.

S8. Viguie CA, Lu DX, Huang SK, Rengen H, Carlson BM. Quantitative study of the effects of long‐term denervation on the extensor digitorum longus muscle of the rat. *The Anatomical Record: An Official Publication of the American Association of Anatomists.* 1997;248(3):346-354.

S9. Milanič T, Kunstelj A, Marš T, Grubič Z. Morphometric characteristics of myonuclear distribution in the normal and denervated fast rat muscle fiber. *Chemico-biological interactions.* 1999;119:321-326.

S10. Dupont-Versteegden EE, Murphy RJ, Houlé JD, Gurley CM, Peterson CA. Mechanisms leading to restoration of muscle size with exercise and transplantation after spinal cord injury. *American Journal of Physiology-Cell Physiology.* 2000;279(6):C1677-C1684.

S11. Schmalbruch H, Lewis D. Dynamics of nuclei of muscle fibers and connective tissue cells in normal and denervated rat muscles. *Muscle & Nerve: Official Journal of the American Association of Electrodiagnostic Medicine.* 2000;23(4):617-626.

S12. Dedkov EI, Kostrominova TY, Borisov AB, Carlson BM. Reparative myogenesis in long‐term denervated skeletal muscles of adult rats results in a reduction of the satellite cell population. *The Anatomical Record: An Official Publication of the American Association of Anatomists.* 2001;263(2):139-154.

S13. Nnodim JO. Testosterone mediates satellite cell activation in denervated rat levator ani muscle. *The Anatomical Record: An Official Publication of the American Association of Anatomists.* 2001;263(1):19-24.

S14. Wada K-I, Takahashi H, Katsuta S, Soya H. No decrease in myonuclear number after long-term denervation in mature mice. *American Journal of Physiology-Cell Physiology.* 2002;283(2):C484-C488.

S15. Dedkov EI, Borisov AB, Wernig A, Carlson BM. Aging of skeletal muscle does not affect the response of satellite cells to denervation. *Journal of Histochemistry & Cytochemistry.* 2003;51(7):853-863.

S16. Roy RR, Zhong H, Siengthai B, Edgerton VR. Activity‐dependent influences are greater for fibers in rat medial gastrocnemius than tibialis anterior muscle. *Muscle & Nerve: Official Journal of the American Association of Electrodiagnostic Medicine.* 2005;32(4):473-482.

S17. Zhong H, Roy RR, Siengthai B, Edgerton VR. Effects of inactivity on fiber size and myonuclear number in rat soleus muscle. *Journal of Applied Physiology.* 2005;99(4):1494-1499.

S18. Aravamudan B, Mantilla CB, Zhan W-Z, Sieck GC. Denervation effects on myonuclear domain size of rat diaphragm fibers. *Journal of applied physiology.* 2006;100(5):1617-1622.

S19. van der Meer SF, Jaspers RT, Jones DA, Degens H. Time‐course of changes in the myonuclear domain during denervation in young‐adult and old rat gastrocnemius muscle. *Muscle & nerve.* 2011;43(2):212-222.

S20. Liu W, Wei-LaPierre L, Klose A, Dirksen RT, Chakkalakal JV. Inducible depletion of adult skeletal muscle stem cells impairs the regeneration of neuromuscular junctions. *elife.* 2015;4:e09221.

S21. Agüera E, Castilla S, Luque E, et al. Denervated muscle extract promotes recovery of muscle atrophy through activation of satellite cells. An experimental study. *Journal of sport and health science.* 2019;8(1):23-31.

S22. Choi JJ, Shin EJ, Han WM, et al. Regenerating motor neurons prime muscle stem cells for myogenesis by enhancing protein synthesis and mitochondrial bioenergetics. *bioRxiv.* 2020.

S23. Xing H-Y, Liu N, Zhou M-W. Satellite cell proliferation and myofiber cross-section area increase after electrical stimulation following sciatic nerve crush injury in rats. *Chinese medical journal.* 2020;133(16):1952.

S24. Wong A, Garcia SM, Tamaki S, et al. Satellite cell activation and retention of muscle regenerative potential after long‐term denervation. *Stem Cells.* 2021;39(3):331-344.

S25. Jackson JR, Mula J, Kirby TJ, et al. Satellite cell depletion does not inhibit adult skeletal muscle regrowth following unloading-induced atrophy. *American Journal of Physiology-Cell Physiology.* 2012;303(8):C854-C861.

S26. Darr KC, Schultz E. Hindlimb suspension suppresses muscle growth and satellite cell proliferation. *Journal of Applied Physiology.* 1989;67(5):1827-1834.

S27. Kasper CE, Xun L. Cytoplasm-to-myonucleus ratios in plantaris and soleus muscle fibres following hindlimb suspension. *Journal of Muscle Research & Cell Motility.* 1996;17(5):603-610.

S28. Allen DL, Linderman JK, Roy RR, Grindeland RE, Mukku V, Edgerton VR. Growth hormone/IGF-I and/or resistive exercise maintains myonuclear number in hindlimb unweighted muscles. *Journal of Applied Physiology.* 1997;83(6):1857-1861.

S29. Mozdziak P, Pulvermacher P, Schultz E. Unloading of juvenile muscle results in a reduced muscle size 9 wk after reloading. *Journal of Applied Physiology.* 2000;88(1):158-164.

S30. Mitchell PO, Pavlath GK. A muscle precursor cell-dependent pathway contributes to muscle growth after atrophy. *American Journal of Physiology-Cell Physiology.* 2001;281(5):C1706-C1715.

S31. Yamazaki T. Influence of hindlimb unweighting and intermittent weight bearing on dynamics of nuclei in rat soleus muscle. *Journal of the Japanese Physical Therapy Association.* 2003;6(1):1-8.

S32. Mitchell PO, Pavlath GK. Skeletal muscle atrophy leads to loss and dysfunction of muscle precursor cells. *American Journal of Physiology-Cell Physiology.* 2004;287(6):C1753-C1762.

S33. Ferreira R, Neuparth MJ, Ascensão A, et al. Skeletal muscle atrophy increases cell proliferation in mice gastrocnemius during the first week of hindlimb suspension. *European journal of applied physiology.* 2006;97(3):340-346.

S34. Wang X, Kawano F, Matsuoka Y, et al. Mechanical load-dependent regulation of satellite cell and fiber size in rat soleus muscle. *American Journal of Physiology-Cell Physiology.* 2006;290(4):C981-C989.

S35. Kawano F, Matsuoka Y, Oke Y, et al. Role (s) of nucleoli and phosphorylation of ribosomal protein S6 and/or HSP27 in the regulation of muscle mass. *American Journal of Physiology-Cell Physiology.* 2007;293(1):C35-C44.

S36. Kawano F, Takeno Y, Nakai N, et al. Essential role of satellite cells in the growth of rat soleus muscle fibers. *American Journal of Physiology-Cell Physiology.* 2008;295(2):C458-C467.

S37. Oishi Y, Ogata T, Yamamoto Ki, et al. Cellular adaptations in soleus muscle during recovery after hindlimb unloading. *Acta physiologica.* 2008;192(3):381-395.

S38. Tarakina M, Turtikova O, Nemirovskaya T, Kokontcev A, Shenkman B. Muscle progenitor cells proliferation doesn’t sufficiently contribute to maintaining stretched soleus muscle mass during gravitational unloading. *Acta Astronautica.* 2008;63(7-10):706-713.

S39. Matsuba Y, Goto K, Morioka S, et al. Gravitational unloading inhibits the regenerative potential of atrophied soleus muscle in mice. *Acta physiologica.* 2009;196(3):329-339.

S40. Kartashkina N, Turtikova O, Kuznetsov S, et al. Effect of NO on satellite cell proliferation during functional unloading and muscle stretching. Paper presented at: Doklady Biological Sciences2010.

S41. Zhang B-T, Yeung SS, Liu Y, et al. The effects of low frequency electrical stimulation on satellite cell activity in rat skeletal muscle during hindlimb suspension. *BMC cell biology.* 2010;11(1):1-9.

S42. Kachaeva E, Turtikova O, Ushakov I, Orlov O, Shenkman B. Postural muscle recovery under lowered oxygen concentration after prolonged disuse. Paper presented at: Doklady Biochemistry and biophysics2011.

S43. Ohira T, Terada M, Kawano F, Nakai N, Ogura A, Ohira Y. Region-specific responses of adductor longus muscle to gravitational load-dependent activity in Wistar Hannover rats. *PLoS One.* 2011;6(6):e21044.

S44. Teixeira CE, Duarte JA. Changes in cross sectional area per myonucleus on mice soleus muscle during one week of hindlimb suspension reinforce the concept of myonuclear domain. *Archives of Exercise in Health & Disease.* 2011;2(1).

S45. Lomonosova YN, Kalamkarov G, Bugrova A, et al. Role of NO-synthase in regulation of protein metabolism of stretched rat m. soleus muscle during functional unloading. *Biochemistry (Moscow).* 2012;77(2):208-216.

S46. Zushi K, Yamazaki T. The effect of reloading on disuse muscle atrophy: time course of hypertrophy and regeneration focusing on the myofiber cross-sectional area and myonuclear change. *Journal of the Japanese Physical Therapy Association.* 2012:1203190016-1203190016.

S47. Itoh Y, Hayakawa K, Mori T, et al. Stand‐up exercise training facilitates muscle recovery from disuse atrophy by stimulating myogenic satellite cell proliferation in mice. *Physiological reports.* 2014;2(11):e12185.

S48. Park S, Brisson BK, Liu M, Spinazzola JM, Barton ER. Mature IGF-I excels in promoting functional muscle recovery from disuse atrophy compared with pro-IGF-IA. *Journal of Applied Physiology.* 2014;116(7):797-806.

S49. Babcock LW, Knoblauch M, Clarke MS. The role of myostatin and activin receptor IIB in the regulation of unloading-induced myofiber type-specific skeletal muscle atrophy. *Journal of applied physiology.* 2015;119(6):633-642.

S50. Ohira T, Wang X-D, Ito T, et al. Macrophage deficiency in osteopetrotic (op/op) mice inhibits activation of satellite cells and prevents hypertrophy in single soleus fibers. *American Journal of Physiology-Cell Physiology.* 2015;308(10):C848-C855.

S51. Nakanishi R, Hirayama Y, Tanaka M, et al. Nucleoprotein supplementation enhances the recovery of rat soleus mass with reloading after hindlimb unloading–induced atrophy via myonuclei accretion and increased protein synthesis. *Nutrition Research.* 2016;36(12):1335-1344.

S52. Itoh Y, Murakami T, Mori T, et al. Training at non‐damaging intensities facilitates recovery from muscle atrophy. *Muscle & nerve.* 2017;55(2):243-253.

S53. Anderson JE, Zhu A, Mizuno TM. Nitric oxide treatment attenuates muscle atrophy during hind limb suspension in mice. *Free Radical Biology and Medicine.* 2018;115:458-470.

S54. Miller BF, Hamilton KL, Majeed ZR, et al. Enhanced skeletal muscle regrowth and remodelling in massaged and contralateral non‐massaged hindlimb. *The Journal of physiology.* 2018;596(1):83-103.

S55. Kneppers A, Leermakers P, Pansters N, et al. Coordinated regulation of skeletal muscle mass and metabolic plasticity during recovery from disuse. *The FASEB Journal.* 2019;33(1):1288-1298.

S56. Nakanishi R, Tanaka M, Maeshige N, Kondo H, Roy RR, Fujino H. Nucleoprotein‐enriched diet enhances protein synthesis pathway and satellite cell activation via ERK1/2 phosphorylation in unloaded rat muscles. *Experimental Physiology.* 2021.

S57. Petrocelli JJ, Mahmassani ZS, Fix DK, et al. Metformin and leucine increase satellite cells and collagen remodeling during disuse and recovery in aged muscle. *The FASEB Journal.* 2021;35(9):e21862.

S58. Smith HK, Maxwell L, Martyn JA, Bass JJ. Nuclear DNA fragmentation and morphological alterations in adult rabbit skeletal muscle after short-term immobilization. *Cell and tissue research.* 2000;302(2):235-241.

S59. Wanek LJ, Snow MH. Activity‐induced fiber regeneration in rat soleus muscle. *The Anatomical Record: An Official Publication of the American Association of Anatomists.* 2000;258(2):176-185.

S60. Ye F, Mathur S, Liu M, et al. Overexpression of insulin‐like growth factor‐1 attenuates skeletal muscle damage and accelerates muscle regeneration and functional recovery after disuse. *Experimental physiology.* 2013;98(5):1038-1052.

S61. Matsumoto Y, Nakano J, Oga S, et al. The non-thermal effects of pulsed ultrasound irradiation on the development of disuse muscle atrophy in rat gastrocnemius muscle. *Ultrasound in medicine & biology.* 2014;40(7):1578-1586.

S62. Guitart M, Lloreta J, Mañas‐Garcia L, Barreiro E. Muscle regeneration potential and satellite cell activation profile during recovery following hindlimb immobilization in mice. *Journal of cellular physiology.* 2018;233(5):4360-4372.

S63. Usuki F, Fujimura M, Nakamura A, Nakano J, Okita M, Higuchi I. Local vibration stimuli induce mechanical stress-induced factors and facilitate recovery from immobilization-induced oxidative myofiber atrophy in rats. *Frontiers in physiology.* 2019;10:759.

S64. Suzuki H, Yoshikawa Y, Tsujimoto H, Kitaura T, Muraoka I. Clenbuterol accelerates recovery after immobilization-induced atrophy of rat hindlimb muscle. *Acta histochemica.* 2020;122(1):151453.

S65. Zazula MF, Wutzke MLS, da Costa JRG, et al. Morphological effects of whole‐body vibration on remobilization of the tibialis anterior muscle of Wistar rats. *The Anatomical Record.* 2020;303(11):2857-2864.

S66. Honda Y, Tanaka N, Kajiwara Y, et al. Effect of belt electrode-skeletal muscle electrical stimulation on immobilization-induced muscle fibrosis. *PloS one.* 2021;16(5):e0244120.

S67. Allen D, Yasui W, Tanaka T, et al. Myonuclear number and myosin heavy chain expression in rat soleus single muscle fibers after spaceflight. *Journal of applied physiology.* 1996;81(1):145-151.

S68. Hikida RS, van Nostran S, Murray JD, Staron RS, Gordon SE, Kraemer WJ. Myonuclear loss in atrophied soleus muscle fibers. *The Anatomical Record: An Official Publication of the American Association of Anatomists.* 1997;247(3):350-354.

S69. Kasper CE, Xun L. Cytoplasm-to-myonucleus ratios following microgravity. *Journal of Muscle Research & Cell Motility.* 1996;17(5):595-602.

S70. McClung JM, Kavazis AN, DeRuisseau KC, et al. Caspase-3 regulation of diaphragm myonuclear domain during mechanical ventilation–induced atrophy. *American journal of respiratory and critical care medicine.* 2007;175(2):150-159.

S71. Dearth CL, Goh Q, Marino JS, et al. Skeletal muscle cells express ICAM-1 after muscle overload and ICAM-1 contributes to the ensuing hypertrophic response. *PLoS One.* 2013;8(3):e58486.

S72. Egner IM, Bruusgaard JC, Gundersen K. Satellite cell depletion prevents fiber hypertrophy in skeletal muscle. *Development.* 2016;143(16):2898-2906.

S73. Huey KA, Smith SA, Sulaeman A, Breen EC. Skeletal myofiber VEGF is necessary for myogenic and contractile adaptations to functional overload of the plantaris in adult mice. *Journal of Applied Physiology.* 2016;120(2):188-195.

S74. Pérez-Schindler J, Summermatter S, Santos G, Zorzato F, Handschin C. The transcriptional coactivator PGC-1α is dispensable for chronic overload-induced skeletal muscle hypertrophy and metabolic remodeling. *Proceedings of the National Academy of Sciences.* 2013;110(50):20314-20319.

S75. Zempo H, Suzuki J-i, Ogawa M, Watanabe R, Isobe M. A different role of angiotensin II type 1a receptor in the development and hypertrophy of plantaris muscle in mice. *Journal of applied genetics.* 2016;57(1):91-97.

S76. Leenders M, Verdijk LB, van der Hoeven L, Van Kranenburg J, Nilwik R, van Loon LJ. Elderly men and women benefit equally from prolonged resistance-type exercise training. *Journals of Gerontology Series A: Biomedical Sciences and Medical Sciences.* 2013;68(7):769-779.

S77. Snijders T, Res PT, Smeets JS, et al. Protein ingestion before sleep increases muscle mass and strength gains during prolonged resistance-type exercise training in healthy young men. *The Journal of nutrition.* 2015;145(6):1178-1184.

S78. Snijders T, Smeets JS, Van Kranenburg J, Kies A, van Loon L, Verdijk LB. Changes in myonuclear domain size do not precede muscle hypertrophy during prolonged resistance‐type exercise training. *Acta Physiologica.* 2016;216(2):231-239.

S79. Verdijk LB, Jonkers RA, Gleeson BG, et al. Protein supplementation before and after exercise does not further augment skeletal muscle hypertrophy after resistance training in elderly men. *The American journal of clinical nutrition.* 2009;89(2):608-616.

S80. Ferguson VL, Ayers RA, Bateman TA, Simske SJ. Bone development and age-related bone loss in male C57BL/6J mice. *Bone.* 2003;33(3):387-398.
